# Catalysis of Native Chemical Ligation and Expressed Protein Ligation by Alkylselenols

**DOI:** 10.1021/jacsau.5c00793

**Published:** 2025-12-02

**Authors:** Iván Sánchez-Campillo, Esther Gratacòs-Batlle, Selene Pérez-García, Hong S. Nguyen, Gemma Triola, Henning D. Mootz, Juan B. Blanco-Canosa

**Affiliations:** † Institute for Advanced Chemistry of Catalonia (IQAC), Spanish National Research Council (CSIC), 08034 Barcelona, Spain; Department of Inorganic and Organic Chemistry, Section of Organic Chemistry, University of Barcelona, 08028 Barcelona, Spain; ‡ Fundamental and Clinical Nursing Department, Faculty of Nursing, Institute of Neurosciences, University of Barcelona, 08907 ĹHospitalet de Llobregat, Spain; § Institute for Advanced Chemistry of Catalonia (IQAC), Spanish National Research Council (CSIC), 08034 Barcelona, Spain; ∥ Laboratoire de Chimie et Biochimie Pharmacologiques et Toxicologiques, UMR 8601 CNRS, Université Paris Cité, 75006 Paris, France; ⊥ Institute of Biochemistry, University of Münster, 48419 Münster, Germany

**Keywords:** Native chemical ligation, expressed protein ligation, selenoesters, SeESNa, peptides

## Abstract

The reaction between C-terminal α-thioester and N-terminal cysteinyl peptides is known as native chemical ligation (NCL). Alkyl α-thioesters are traditionally prepared in NCL due to their higher thermodynamic stability, which endows resistance to hydrolysis and easier peptide handling. However, the ligation kinetics of these species are slow, and the reaction times exceed the practical limits for chemical protein synthesis. Therefore, conversion to a more reactive phenyl α-thioesters through thiol-thioester exchange is usually employed to enhance the NCL reaction rate. In addition, phenyl thiols can reverse the formation of the less reactive branched thioesters, i.e., thioesters formed with internal Cys residues, and thiolactones. Interestingly, the fastest NCL rates are achieved with phenyl α-selenoesters, though phenylselenol is a poor catalyst for the selenol-(α-alkyl thioester) exchange, particularly with β-branched residues. Hence, it is usually necessary to preform the phenyl α-selenoester and protect internal cysteine residues to preserve the kinetic advantage. Moreover, an ∼2–5-fold excess of the N-terminal cysteine acceptor peptide is typically required to prevent the competition from the cysteine of the ligation product for the phenyl α-selenoester donor that leads to the branched thioester formation. Based on these precedents, we have designed sodium 2-selenoethanesulfonate (SeESNa) as a new selenol catalyst that can overcome these limitations. SeESNa reacts with alkyl α-thioester, *N*-acyl benzotriazole, and *N*-acylurea peptides, giving α-SeESNa species. We have determined the rate constants for the ligation with preformed α-SeESNa peptides and show that it is a superior catalyst compared to the known 4-mercaptophenylacetic and 4-mercaptobenzoic acids. The utility of SeESNA was proved through the synthesis of the cardiotoxin A5, a snake venom peptide that contains eight cysteines, without orthogonal cysteine protection. Importantly, it has enabled expressed protein ligation under folding conditions with extraordinary speed, as shown with the Sonic Hedgehog and SUMO2 proteins. Thus, SeESNa is envisaged to have broad applicability in synthetic and semisynthetic protein chemistry.

## Graphical abstract

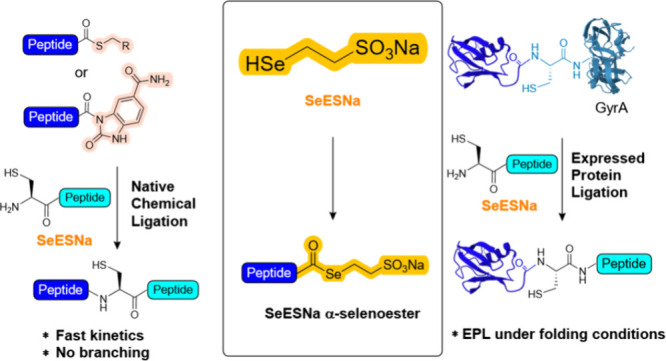

## Introduction

Native chemical ligation (NCL) is an acyl transfer reaction primarily used for the condensation of unprotected peptides in aqueous solution.^1,2^ NCL and solid-phase peptide synthesis (SPPS) have enabled the assembly of medium-sized proteins with full atomic control over the sequence, allowing the design and introduction of chemical modifications and topologies that are difficult to obtain even by recombinant expression methodologies. It is conventionally carried out at room temperature and neutral pH. The ligation mechanism describes a two-step pathway between a peptide presenting a C-terminal α-thioester and a second bearing an N-terminal cysteine. The Cys thiolate attacks the α-thioester, forming a transient thioester in the first phase. This transthioesterification is followed by an intramolecular *S-to-N* acyl migration through a 5-membered ring transition state, to yield the final amide bond (Scheme 1).^2–4^

**Scheme 1:**
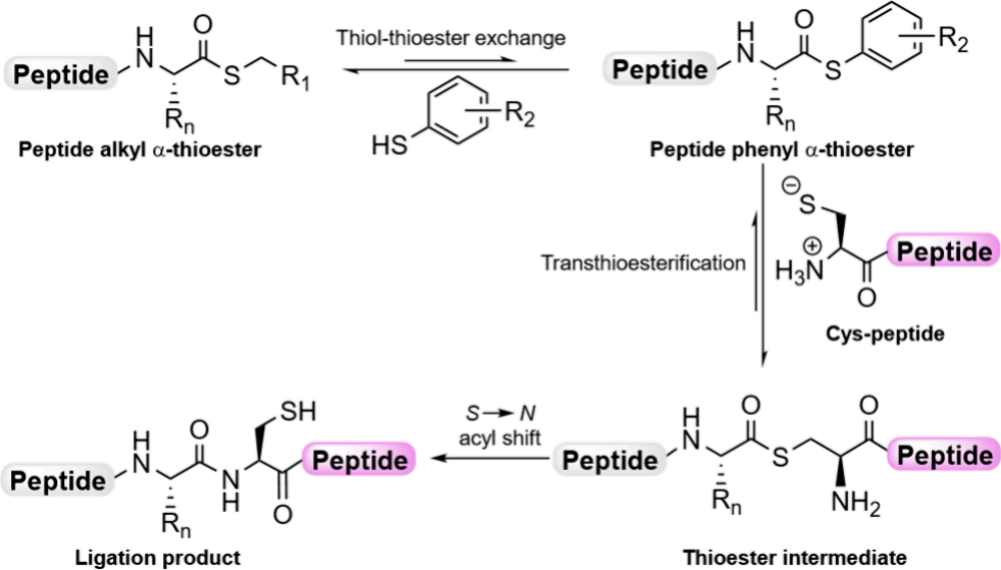
Mechanism of NCL Catalyzed by Phenyl Thiols

The nature of the C-terminal α-thioester residue plays a key role in the NCL rate.^1^ This comprises two parts: the side-chain steric impediment of the amino acid and the thiol leaving group. Thus, bulky side chains negatively affect the reaction rate, and residues like Val and Ile have some of the slowest kinetics.

The p*K*_a_ of the thiol leaving group is the other rate-determining factor: lower p*K*_a_ values are correlated with better leaving groups and are directly proportional to faster acylation velocities. This means that phenyl thiols (p*K*_a_ *∼* 5.5–7) are ∼1–2 orders of magnitude more reactive than alkyl thiols (p*K*_a_ ∼ 8.5–9.5). However, the higher thermodynamic stability of alkyl α-thioesters has prioritized their synthesis through traditional Boc-SPPS.^1,5,6^ Despite this reduced reactivity, the ligation is accelerated by incubation with a large excess of phenyl thiols (100–200 mM) that catalyze the thiol-thioester exchange, forming highly reactive phenyl α-thioesters. These phenyl thiols also dissociate other thermodynamically stable and less reactive intermediates, such as branched thioesters or thiolactones that are formed when an internal Cys is present in the thioester fragment (Figure 1a).^3,7^ Contemporary methods for the preparation of α-thioester peptides use Fmoc-SPPS and rely on different precursors. Those include the *N*-acylurea (Nbz),^8–11^ *N*-acyl benzotriazole (Bt),^12^ *N*-acyl azide,^13^ *N*-acyl pyrazole,^14^ bis­(2-sulfanylethyl)­amido (SEA),^15^ and others.^16–18^ These surrogates undergo thioesterification in the presence of thiols. Importantly, the acyl-thiol capture is virtually irreversible in the majority of these approaches, and quick exchanges (<30 min) are achieved at neutral or slightly acidic pH.

**Figure 1:**
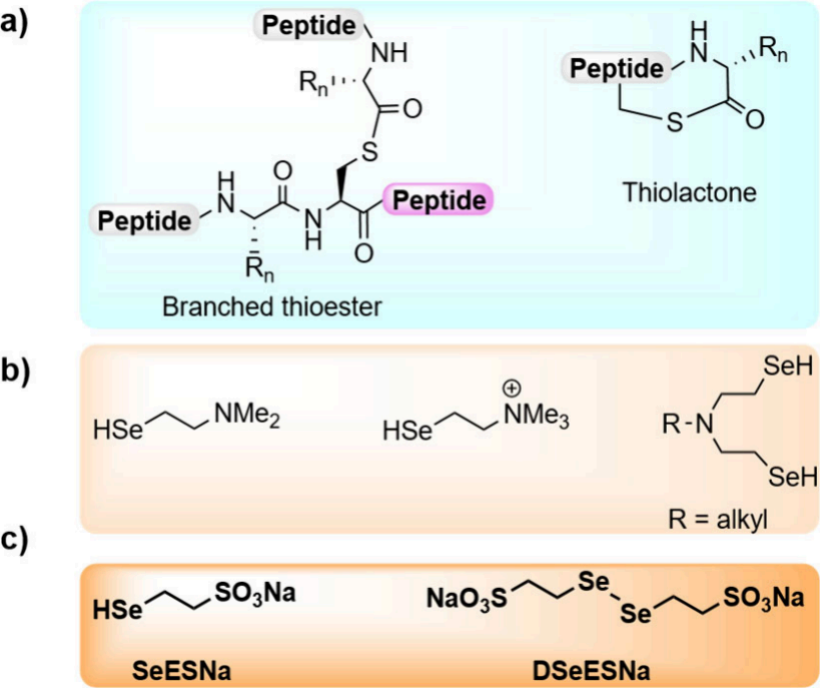
(A) Representation of branched thioesters and thiolactones. (B) Selenols used in SEA catalysis. (C) SeESNa and DSeESNa alkyl selenols developed in this study.

Modern NCL strategies have also incorporated phenyl α-selenoesters as acyl donors. These energy-rich compounds have significantly improved the ligation kinetics, achieving rate enhancements of over 1 order of magnitude compared to phenyl α-thioesters.^19,4^ Remarkably, this high reactivity has been leveraged to develop a new diselenide-selenoester ligation (DSL) where a C-terminal phenylselenoester peptide is ligated to an N-terminal diselenide fragment with or without prior diselenide reduction.^20–22^ The increased phenyl α-selenoester acylation power is mainly credited to the lower p*K*_a_ of phenylselenol: ∼ 4.6 versus 5.5–6.8 of phenyl thiols such as 4-mercaptobenzoic acid (4-MBA), 4-mercaptophenylacetic acid (4-MPAA), 4-mercaptophenol, and thiophenol. However, this enhanced acylating strength is counteracted by a relatively slow phenylselenol-thioester exchange rate with alkyl-type α-thioesters, such as branched thioesters and thiolactones, due to the large p*K*_a_ difference with the thiol group of internal Cys residues (p*K*_a_ ∼ 8.5, about 4 orders of magnitude). As a result, this can lead to the accumulation of these less reactive species. Therefore, it is recommended to add an excess of the Cys peptide (∼ 2–5 equiv, depending on the C-terminal selenoester residue) and protect internal Cys residues to avoid their appearance.

The search for efficient catalysts has been a constant effort in NCL. They should have improved H_2_O solubility, be less malodorous, and be compatible with *in situ* postligation desulfurization. This led to the discovery of 4-MPAA and others.^7,16^ Some studies in this direction using SEA peptides have shown that several alkylselenols and alkyldiselenols can catalyze the thiol-thioester exchange and increase the ligation rate (Figure 1b).^23,24^ Nonetheless, these studies were conducted using the SEA-mediated ligation, carried out mainly at acidic pH ∼ 4, and only to form phenyl α-thioesters for NCL, but not to transfer the acyl donor to the acceptor Cys peptide. Moreover, there is also a lack of understanding of how a selenol catalyst could perform under more standard NCL conditions, i.e., pH 7, and with peptides containing multiple Cys residues in which thiols such as 4-MPAA are particularly efficient. It is also unknown if selenols could attack and cleave intein thioesters to catalyze expressed protein ligation (EPL).^25–27^ Consequently, NCL catalysis by selenols is underexplored, and a broader study of selenols and α-selenoesters and their use in NCL will definitely shed light on the search for new selenol-based NCL catalysts. These catalysts could overcome the limitations of phenylselenol to cleave branched thioesters and increase the final yield of the ligation product.

Based on these premises, herein we describe sodium 2-selenoethanesulfonate (**SeESNa**, Figure 1c), which can be obtained through *in situ* reduction of the diselenide di­(sodium 2-selenoethanesulfonate) (**DSeESNa**). SeESNa is a better acyl donor than 4-MBA and 4-MPAA α-thioesters, and it catalyzes the selenol-thioester exchange with branched thioesters at neutral pH faster than phenyl thiols performing the equivalent thiol-thioester reaction. Moreover, peptide *N*-acylureas and *N*-acyl benzotriazoles can undergo acyl transfer in the presence of SeESNa, giving α-selenoester species. In addition, we show that SeESNa intercepts intein-generated recombinant protein thioesters to catalyze expressed protein ligation (EPL).

## Results and Discussion

### Di­(sodium 2-selenoethanesulfonate) (DSeESNa)

The equilibrium between phenylselenol and phenyl or alkyl α-thioesters is shifted toward the thioester form by >50% due to the large difference in p*K*_a_ (∼2 and 5 orders of magnitude, respectively). We anticipated that a selenol with a higher p*K*_a_ could have a reduced acylating strength but stronger nucleophilic character. Consequently, the α-selenoester form would be more populated in the equilibrium with α-thioesters. Therefore, we took sodium 2-mercaptoethanesulfonate (MESNa, p*K*_a_ ∼ 9.08) as a reference,^28^ and predicted that the selenium congener should have a p*K*_a_ 2–3 orders of magnitude lower. To this end, DSeESNa was initially prepared from the commercially available sodium 2-bromoethanesulfonate and elemental selenium (Scheme 2a). Reduction of selenium powder with sodium borohydride to generate Na_2_Se_2_,^29^ followed by *in situ* reaction with 2-bromoethanesulfonate, provided the expected diselenide DSeESNa (35% yield at gram scale after workup and purification). The characterization of this product by ^1^H, ^13^C, and ^11^B NMR, elemental, and thermogravimetric analysis provided a likely composition corresponding to a molecular formula = C_4_H_8_O_6_S_2_Se_2_Na_2_·(H_2_O)_2_·(H_3_BO_3_). SeESNa can be generated during NCL through the *in situ* DSeESNa reduction with TCEP and ascorbate. Gratifyingly, DSeESNa and SeESNa are both highly soluble in H_2_O (DSeESNa saturation >0.8 M), and no unpleasant smell is released from DSeESNa nor from SeESNa. The compound is also air-stable and can be stored for months at room temperature in a closed vial protected from light. The p*K*_a_ of the SeESNA selenol was determined by potentiometric titration, resulting in 6.05 ± 0.04. This value represents ∼ 1.5 orders of magnitude larger than phenylselenol and 3 orders smaller than MESNa, and is in the range between 4-MBA (p*K*_a_ = 5.5) and 4-MPAA (p*K*_a_ = 6.6). Therefore, both are good thiols to compare the relative reactivity with SeESNa in NCL.

**Scheme 2:**
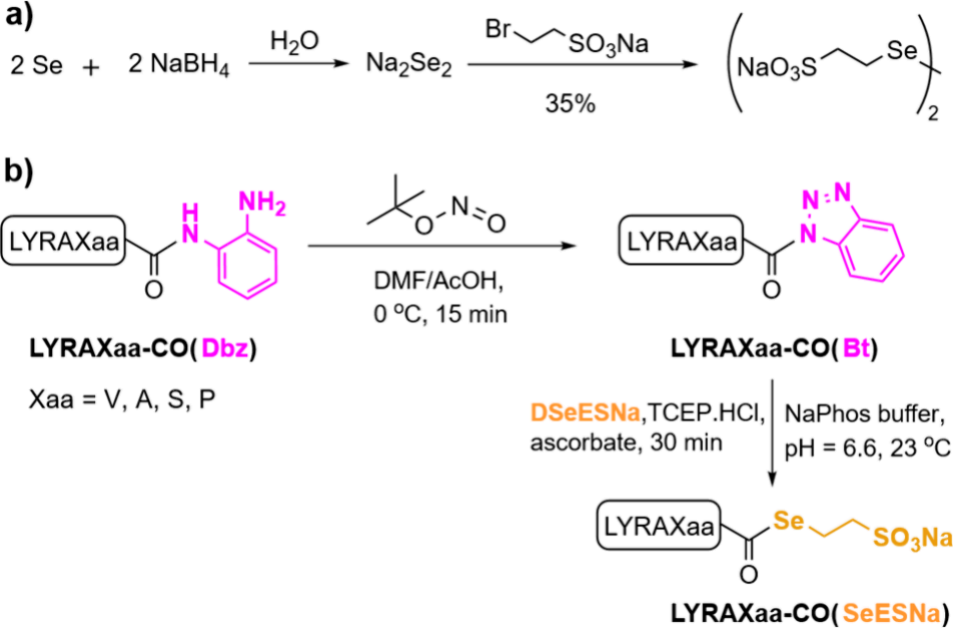
(A) Synthesis of DSeESNa; (B) Preparation of SeESNa α-Selenoester Peptides

### Synthesis of α-SeESNa Peptides

We initially focused our study on peptides containing a C-terminal Val to compare the reactivity of α-thioesters and α-selenoesters. This residue is a frequent ligation junction due to its high occurrence in proteins (∼6.3%), and the kinetics displayed by C-terminal Val α-thioesters are quite similar to those observed with other β-branched residues (Ile, Thr). In addition, the ligation of LYRAV-CO­(S­(CH_2_)_2_CONH)-Leu catalyzed with thiophenol has a slow rate (>48 h at 1–3 mM concentrations of reactants).^1^ This result is the combination of two factors: the side chain steric hindrance and the sluggish phenyl thiol-thioester substitution rate, likely due to the limited solubility of thiophenol in H_2_O.^1,3^ Remarkably, the phenyl α-thioester formation rate significantly increases in the presence of more soluble phenyl thiols, such as 4-MPAA and 4-mercaptophenol, leading to an important NCL acceleration.^7,9^ Hence, Val is a challenging ligation site and a suitable residue to compare the relative kinetics of different C-terminal α-selenoesters and α-thioesters. Thus, we prepared different model peptides: LYRAV-CO­(XR) (X = Se, S; R = sodium ethanesulfonate, phenylacetic acid). We also include in the evaluation the results previously obtained with the phenyl α-selenoester and 4-MBA α-thioester.^4^ In addition, other LYRAXaa-CO­(SeESNa) (Xaa = A, S, P) substrates, presenting distinct C-terminal residues, were synthesized with the intention to cover the rate profile of the 20 standard amino acids. The synthesis of SeESNa α-selenoester peptides was straightforwardly accomplished by reaction of Bt peptides with SeESNa (Scheme 2b). First, oxidation of LYRAXaa-CO­(Dbz) (Dbz = *o*-aminoanilide) with *tert*-Butyl nitrite (^*t*^BuONO) in a DMF:AcOH (9:1) solution at 0 °C gave the corresponding LYRAXaa-CO­(Bt) peptides. After precipitation of the crude residue in cold Et_2_O, the resulting LYRAXaa-CO­(Bt) was dissolved in aqueous buffer (pH = 6.6) containing SeESNa, obtained through reduction of DSeESNa with TCEP and ascorbate. The expected LYRAXaa-CO­(SeESNa) products were recovered in good overall yields after HPLC purification (>50%) following the acyl transfer. The presence of ascorbate prevented the deselenization of SeESNa by TCEP through a mechanism similar to that reported for selenocysteine.^30,31^

### NCL at C-Terminal Val-CO­(SeESNa)

The NCL between LYRAV-CO­(SeESNa) (**1**, 1.9 mM) and the N-terminal Cys peptide CTAFS (**2**, 2.7 mM, 1.4 equiv) was carried out under denaturing ligation conditions in phosphate buffer containing guanidine hydrochloride (Gdm.HCl, 5.0 M), sodium phosphate (NaPhos, 0.17 M), DSeESNa (50 mM), TCEP (100 mM), ascorbic acid (100 mM), H-Tyr-OH (2.0 mM), at pH = 7.0 (Figure 2a). The complete reduction of DSeESNa to SeESNa was achieved upon mixing with TCEP. The reaction was monitored by RP-HPLC with simultaneous detection at 220 and 280 nm (Figure 2b), and the concentration of all Tyr-containing products was determined at 280 nm using H-Tyr-OH as an internal reference. The ligation achieved ∼95% conversion in 3 h and, gratifyingly, no significant signal of the branched peptide **4** was detected at the end of the ligation. These results are in contrast with those achieved when the reaction was performed with phenylselenol, where 16% of the branched product was obtained using a 1.5-fold excess of the Cys peptide.^4^ However, in our study, the maximum concentration of **4** observed during the ligation was 0.076 mM, which represented less than 5% of the total product formation (Figure 2c). This kinetic profile reflects a fast equilibrium where SeESNa rapidly dissociates **4** to regenerate **1** and **3**. Importantly, it implies that the NCL using SeESNa does not require previous Cys protection or a large excess of the Cys peptide.

**Figure 2:**
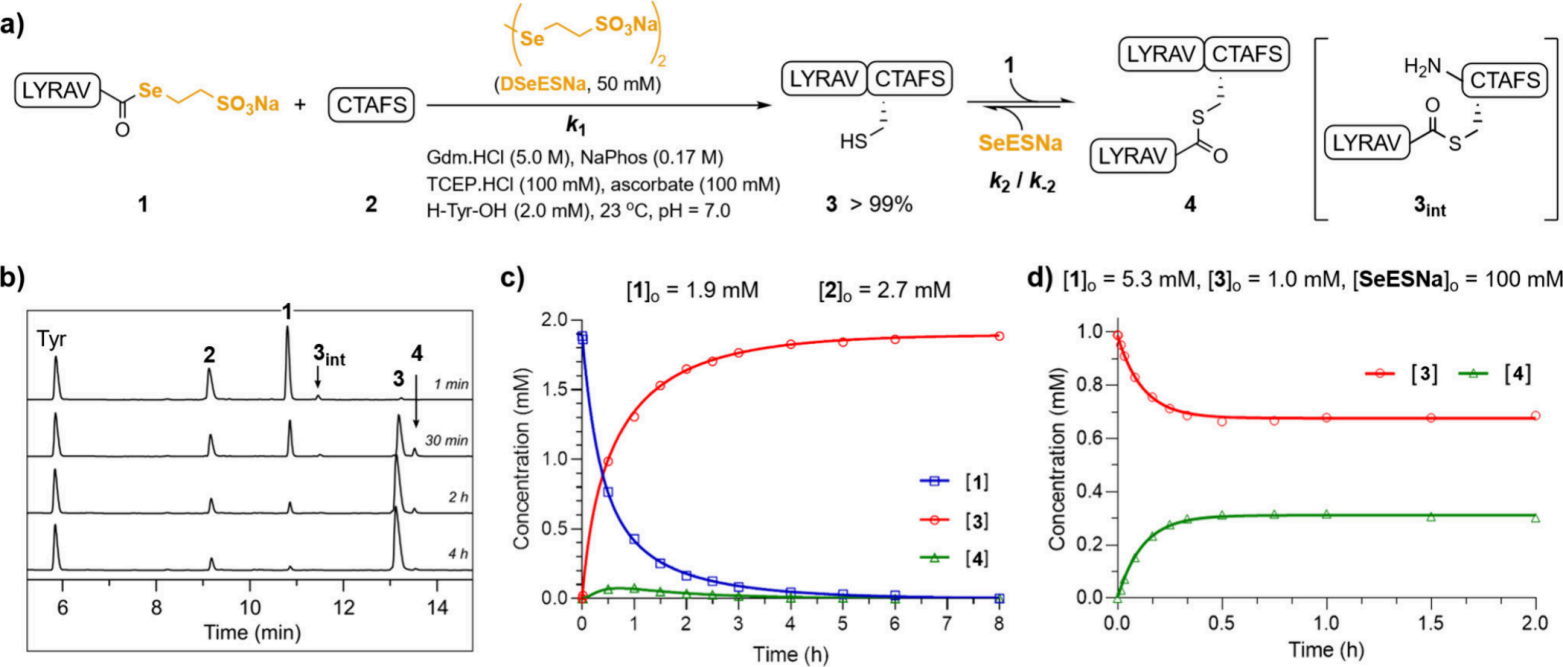
(A) NCL between peptides **1** and **2** in the presence of SeESNa. (B) Representative HPLC traces (λ = 220 nm) of the ligation at the indicated times. (C) Curve fitting of the experimental data (dots) according to the model: **1** + **2** → **3** + **1** ⇌ **4** + **SeESNa**. (D) Curve fitting of the experimental data (dots) using the model: **3** + **1** ⇌ **4** + **SeESNa**. Gdm.HCl = guanidine hydrochloride, NaPhos = sodium phosphate. **3_int_** = intermediate thioester **3**.

Interestingly, the transient *S*-acyl intermediate thioester (**3**_**int**_) was detected during the first minute of the ligation. However, its concentration was only 0.053 mM, 7-fold lower than the one observed with the ligation of the phenyl α-selenoester under similar conditions of peptide concentration and pH.^4^ This low accumulation infers a simplified kinetic model where the intermediate **3**_**int**_ is eliminated from the general model (eq 1), providing a one-step bimolecular reaction between the selenoester **1** and the Cys-peptide **2** to yield the product **3**. The two-step model (eq 2) includes the equilibrium among **3**, **1**, **4**, and **SeESNa**:

1 $$1+2\overset{k_{1}}{\to }3_{\mathrm{int}}\overset{k_{\mathrm{int}}}{\to }3+1\begin{array}{l} k_{2}\\ ⇌\\ k_{-2} \end{array}4+\mathrm{SeESNa}$$

2 $$1+2\overset{k_{1}}{\to }3+1\begin{array}{l} k_{2}\\ ⇌\\ k_{-2} \end{array}4+\mathrm{SeESNa}$$

We define *k*_1_ as the apparent rate constant for the product formation (**3**). *k*_2_, *k*_–2_ represent the rate constants for the formation and dissociation of **4**, respectively, in the equilibrium. Thus, the reaction profiles of **1**, **3**, and **4** were fitted to eq 2 using simulations of elementary bimolecular reaction steps.^32^ The results, averaged from independent replicates, appear summarized in Table 1 (entry 1), and gave a *k*_1_ = (0.26 ± 0.04) M^–1^s^–1^, *k*_2_ = (0.14 ± 0.02) M^–1^s^–1^, *k*_–2_ = (0.014 ± 0.003) M^–1^s^–1^, *k*_eq_ = 10 ± 2. The forward branching reaction (*k*_2_) is 10-fold faster than the backward reaction (*k*_–2_), and the equilibrium is leaned toward the product **4**. However, the relatively high reverse rate and the [SeESNa] in solution (100 mM) push kinetically this equilibrium to the left side of the equation, affording a higher concentration of reactants **3** and **1**, and thus determining the low accumulation of **4** during the ligation. To corroborate these results, the incubation of **3** (1.0 mM), **1** (5.3 mM), and SeESNa (DSeESNa 50 mM + TCEP 100 mM): **3** + **1** ⇌ **4** + **SeESNa**, reaches the equilibrium after 30 min, giving a [**3**] = 0.69 mM, and [**4**] = 0.31 mM (Figure 2d). The calculated *k*_2_ and *k*_–2_ numbers were in the same order as those computed using eq 2 (averaged together in entry 1, Figures S30, S31, and Table S5).

**Table 1:** Kinetic Constants for the NCL of Model C-Terminal Val Peptides with CTAFS^a^

Entry	LYRAV-CO(XR)	p*K*_a_ RXH^e^	*k*_1_ (M^–1^ s^–1^)	*k*_1_ ratio	*k*_2_ (M^–1^ s^–1^)	*k*_–2_ (M^–1^ s^–1^)
1^b^	SeESNa (**1**)	6.05	0.26 ± 0.04	1	0.14 ± 0.02	0.014 ± 0.003
2^c^	4-MPAA	6.6	0.15 ± 0.01	0.6	0.065 ± 0.003	0.0046 ± 0.0003
3^d^	4-MBA	5.5	0.23 ± 0.01	0.9	0.11 ± 0.02	0.0010 ± 0.0003
4^d^	SePh	4.6	3.2 ± 0.1	12	1.9 ± 0.1	n.d.

a Buffer composition: [Gdm] = 5.0 M, [NaPhos] = 0.17 M, [H-Tyr-OH] = 2.0 mM, pH = 6.9 – 7.0, 23 °C.

b Reaction conditions: [DSeESNa] = 50 mM, [TCEP] = 100 mM, [ascorbate] = 100 mM.

c Reaction conditions: [MPAA] = 100 mM, [TCEP] = 50 mM.

d Data from ref 4.

e p*K*_a_ values for 4-MPAA and 4-MBA were obtained from references 4, 28 ; n.d. = not determined.

Under these ligation conditions (1.4 equiv of peptide **2**), the transient thioester (**3**_**int**_) is only detected at very low concentrations. Simulations using the kinetic model depicted in eq 1 with a *k*_1_ = 0.26 M^–1^s^–1^, the rate constant for the **3**_**int**_ → **3** rearrangement calculated from the phenyl α-selenoester ligation (*k*_int_ = 0.016 s^–1^),^4^ and a 5-fold excess of **2**, predict a highest [**3**_**int**_] of 10% considering the total final product. Confirming this hypothesis, the ligation of **1** (1.8 mM) and **2** (8.3 mM) delivered an equivalent *k*_1_ = (0.31 ± 0.03) M^–1^s^–1^, and the same *k*_int_ = (0.016 ± 0.003) s^–1^, displaying a maximum [**3**_**int**_] = 0.17 mM at 2 min (Figures S33, S34, and Table S6).

### Comparison of the NCL with Different LYRAV-CO­(XR) Acyl Donors

The acyl donor and nucleophilic characteristics of SeESNa were compared to those from LYRAV-CO­(SePh), LYRAV-CO­(4-MPAA), and LYRAV-CO­(4-MBA) under similar ligation conditions: Gdm.HCl (5.0 M), NaPhos (0.17 M), TCEP (50 mM or 100 mM), to have a better understanding of its properties (Figure 3, Table 1). The ligation buffer included the corresponding thiol or selenol: 4-MPAA (100 mM), diphenyl diselenide (DPDS 50 mM + TCEP 100 mM),^4^ or 4-MBA (100 mM).^4^ As expected, the obtained *k*_1_ and *k*_2_ for SeESNa are 12 and 14-fold smaller than **LYRAV-CO­(SePh)** (Table 1, entry 4), a dissimilarity attributed to their distinct p*K*_a_. However, a striking difference exists in *k*_–2_, a value that we estimate at least 1 order of magnitude larger for SeESNa based on simulations of the ligation LYRAV-CO­(SePh) + CTAFS and the kinetic model described in eq 1. Compared to **LYRAV-CO­(4-MPAA)** (Table 1, entry 2), the SeESNa constants are 1.8, 2.2, and 3.0 times bigger (*k*_1_, *k*_2_, *k*_–2_, respectively).

**Figure 3:**
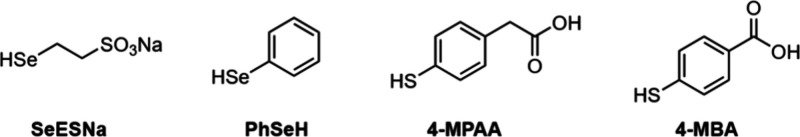
Structure of the thiol and selenol leaving groups compared in this study.

The variations in *k*_1_ and *k*_2_ are explained based on their different p*K*_a_ (6.05 versus 6.6 for 4-MPAA). Interestingly, the *k*_–2_ value reflects a superior nucleophilic strength of SeESNa, although the p*K*_a_ of 4-MPAA is higher. When compared to **LYRAV-CO­(4-MBA)** (p*K*_a_ = 5.5) (Table 1, entry 3), the rate constants *k*_1_ and *k*_2_ are more similar according to their p*K*_a_ values, although still better for **1** by 1.1 and 1.3-fold, respectively. However, the most notable difference is in *k*_–2_, 1 order of magnitude (14-fold) larger for SeESNa. This implies that, despite their similar acylation kinetics, SeESNa is better at cleaving branched thioesters than 4-MBA. These results demonstrate a clear tendency and point to SeESNa as a better NCL catalyst compared to 4-MPAA, 4-MBA, and phenylselenol, even being an inferior acyl donor compared to phenyl α-selenoesters.

To improve the reaction rate, the trans-selenoesterification between **1** and phenylselenol (DPDS 50 mM + TCEP 100 mM) yielded a conversion >99% in 2 h. Based on the product formation, a *k*_obs_ = (0.71 ± 0.05) M^–1^s^–1^ was calculated (Table S8), which comprises both the LYRAV-CO­(SeESNa) and the LYRAV-CO­(SePh) ligations. However, despite this rate acceleration, it is important to note that the branched peptide **4** (13%) is also obtained after consumption of **1** due to the mentioned slow phenylselenol-thioester exchange rate (Figures S38, S39, and S40). On the other hand, the reaction between **4** and **2** to give the product **3** in the presence of phenylselenol (DPDS 50 mM + TCEP 100 mM) required >9 h to fully cleave the branched thioester **4**, indicating the reduced nucleophilicity of phenylselenol compared to SeESNa (Figure S47).

### NCL at C-Terminal Val-CO­(MESNa)

C-terminal MESNa α-thioesters are important intermediates in NCL. MESNa is a versatile thiol used to capture thioester surrogates such as acyl-CO­(Nbz/Bt/azide/pyrazole) derivatives, forming thermodynamically stable alkyl α-thioesters. This stability could be leveraged in kinetic-controlled ligations due to their lower reactivity compared to phenyl α-thioesters.^33^ It is also highly valuable in the transthioesterification of recombinant protein thioesters generated through inteins for EPL, hiding other thiols that have better performance in NCL. This overwhelming use in EPL is likely due to its higher water solubility and the need to perform the intein-mediated thioester generation under native protein conditions.

Therefore, we investigated the ligation between LYRAV-CO­(MESNa) (**5**, 2.1 mM) and CTAFS (**2**, 2.8 mM) catalyzed by SeESNa ([DSeESNa] = 50 mM, [TCEP] = 100 mM, [ascorbate] = 100 mM), at pH = 7.0 under denaturing conditions to compare the kinetics with the acyl donors described in the Table 1 (Figure 4a). The reaction achieved completion in ∼ 6 h (98% conversion, Figure 4b), nearly at the same time as when LYRAV-CO­(MESNa) was consumed, thus illustrating the viability of SeESNa in NCL with MESNa α-thioesters. The kinetic profile shows similar concentrations of **1** and the product **3** at 30 min (∼ 0.5 mM). After 1 h, the [**5**] and [**1**] are similar and decrease at a parallel rate. The [**3**]_t_ and [**4**]_t_ were fitted to a multistep model (**5** + **SeESNa** ⇌ **1** + **MESNa** + **2** → **3** + **1** ⇌ **4** + **SeESNa**, Supporting Information page S31) in where *k*_1_, *k*_2_, and *k*_–2_ were known (0.26 M^–1^s^–1^, 0.14 M^–1^s^–1^, and 0.014 M^–1^s^–1^, respectively) and fixed (Figures S42, S43 and Table S9). Despite the system complexity, a tentative constant for *MESNa-to-SeESNa* exchange was obtained, *k*_S→Se_ = (4.3 × 10^–3^ ± 5 × 10^–4^) M^–1^s^–1^. Remarkably, this value agrees very well with the results achieved for the equilibrium: **1** + **MESNa** ⇌ **5** + **SeESNa**, *k*_Se→S_ = (0.17 ± 0.01) M^–1^s^–1^, *k*_S→Se_ = (5.0 × 10^–3^ ± 2 × 10^–4^) M^–1^s^–1^, *k*_eq_ = 35 ± 3 (Figures 4c, S45, and S46 and Table S10). As expected, the exchange rate *k*_S→Se_ is smaller than the *k*_–2_ of the equilibrium with the branched product **4** (**3** + **1** ⇌ **4** + **SeESNa**, *k*_–2_ = 0.014 M^–1^s^–1^), which reflects the higher p*K*_a_ of MESNa (9.08) compared to the internal sulfhydryl group of a Cys residue (p*K*_a_ ∼ 8.3–8.5). Moreover, when the ligation was carried out in the presence of phenylselenol (DPDS 50 mM + TCEP 100 mM), the percentage of ligated product **3** after 44 h was 79%, representing a slower reaction than the same ligation catalyzed by phenyl thiols (Figure S48). Additionally, the branched peptide **4** was also formed (3%).

**Figure 4:**
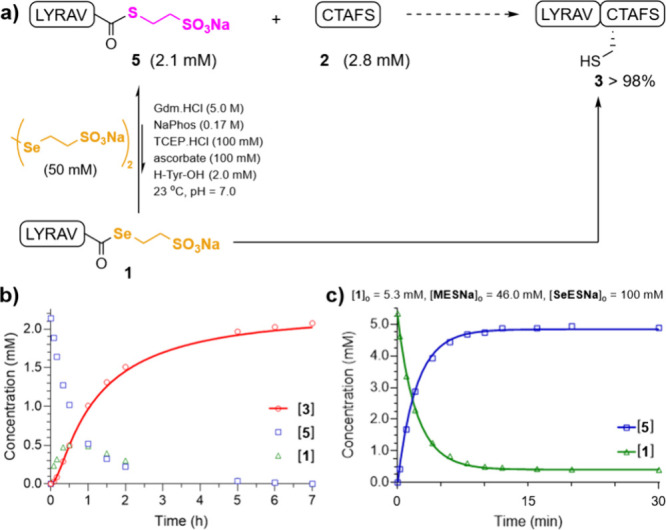
(A) NCL between peptides **5** and **2** in the presence of SeESNa. (B) Kinetic profile of the NCL between LYRAV-CO­(MESNa) and CTAFS catalyzed by SeESNa. [**3**]_t_ was fitted to a multistep model: **5** + **SeESNa** ⇌ **1** + **MESNa** + **2** → **3** + **1** ⇌ **4** + **SeESNa**. (C) Kinetic profile and curve fitting of the experimental data (dots) using the model: **1** + **MESNa** ⇌ **5** + **SeESNa**. Gdm.HCl = guanidine hydrochloride, NaPhos = sodium phosphate.

### NCL at C-Terminal Val-CO­(Nbz)-G

Once the ability of SeESNa to exchange with thioesters was established, we next investigated the ligation of Nbz peptides catalyzed by SeESNa, since C-terminal α-Nbz species are one of the most popular thioester precursors in synthetic protein chemistry.^34–36^ With this aim, LYRAV-CO­(Nbz)-G (**6**, 1.3 mM) and **2** (2.7 mM) were mixed in NaPhos buffer (0.17 M, pH = 7.0) containing Gdm.HCl (5.0 M), TCEP (100 mM), DSeESNa (50 mM), ascorbate (100 mM), and H-Tyr-OH (2.0 mM) (Figure 5a). Pleasantly, a conversion of 95% was achieved in 4 h, confirming the Nbz-SeESNa exchange under ligation conditions (Figure 5b). For comparison, the same ligation catalyzed with 4-mercaptophenol required >10 h to reach a conversion of 95%.^9^ The calculated LYRAV-CO­(Nbz)-G + SeESNa → LYRAV-CO­(SeESNa) exchange rate was *k*_Nbz→Se_ = (2.9 × 10^–3^ ± 1 × 10^–4^) M^–1^s^–1^ (Figures S51, S52, and Table S11). Interestingly, the ligation rate is similar to that observed for LYRAV-CO­(MESNa), although the *k*_Nbz→Se_ is smaller than the obtained *k*_S→Se_. That happens likely due to the absence of an Nbz–SeESNa equilibrium, which accelerates the consumption of the α-Nbz-G peptide (3 h versus 6 h for the MESNa α-thioester): LYRAV-CO­(Nbz)-G + SeESNa → LYRAV-CO­(SeESNa) + Nbz-G.

**Figure 5:**
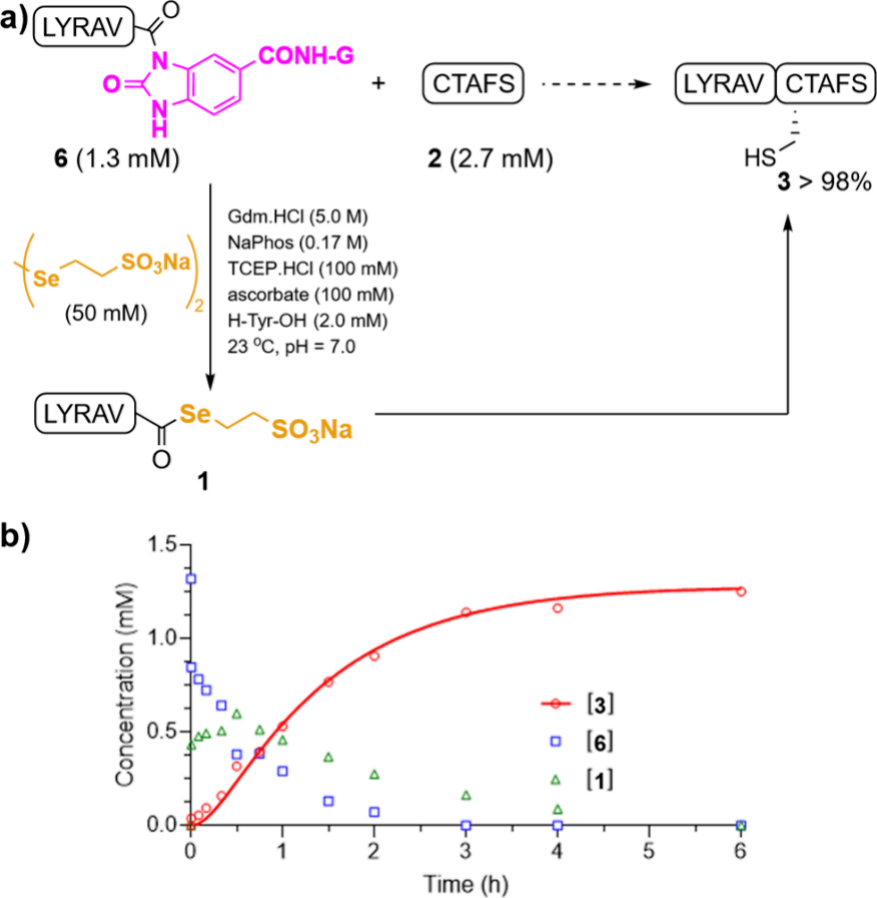
(A) NCL between **6** and **2** catalyzed by SeESNa. (B) Kinetic profile of the NCL between LYRAV-CO­(Nbz)-G and CTAFS catalyzed by SeESNa. [**3**]_t_ was fitted to a multistep model: **6** + **SeESNa** → **1** + **Nbz-G** + **2** → **3** + **1** ⇌ **4** + **SeESNa**. Gdm.HCl = guanidine hydrochloride, NaPhos = sodium phosphate.

### NCL at Ala, Ser, and Pro

Next, we ligated other representative C-terminal residues, including Ala, Ser, and Pro, to evaluate the scope of SeESNa. Both Ala and Ser are α-substituted and have shown fast kinetics in phenyl α-thioester peptides, with conversions >95% in 1 h at millimolar concentrations of reactants.^7,9^ The NCL with preformed α-SeESNa peptides achieved full conversion in ∼ 15 min (Ala) and around 20 min (Ser). That provided second-order rate constants *k*_1_ (Ala) = (4.5 ± 0.9) M^–1^s^–1^, and *k*_1_ (Ser) = (3.2 ± 0.2) M^–1^s^–1^ (Table 2, entries 3 and 4, respectively).

**Table 2:** Kinetic Constants for the NCL of Model SeESNa α-Selenoesters with CTAFS^a^

Entry	LYRAXaa-CO(SeESNa)	*k*_1_ (M^–1^s^–1^)	*k*_1_ ratio
1	V	0.26 ± 0.04	1
2	P	0.060 ± 0.009	0.2
3	A	4.5 ± 0.9	17
4	S	3.2 ± 0.2	12

a Buffer composition: [Gdm] = 5.0 M, [NaPhos] = 0.17 M, [H-Tyr-OH] = 2.0 mM, pH = 6.9 – 7.0, 23 °C. Reaction conditions: [DSeESNa] = 50 mM, [TCEP] = 100 mM, [ascorbate] = 100 mM.

The epimerization of the C-terminal amino acid is a problem with new NCL acyl donors, particularly at racemization-prone residues, like Ser.^37–39^ Gratifyingly, we found epimerization levels <0.1% for the NCL between LYRAS-CO­(SeESNa) and CTAFS (Figure S64).

The C-terminal Pro is a challenging residue displaying the slowest kinetics, as a consequence of an n → π* interaction between the carbonyl orbitals of Pro and the −1 amino acid. This interaction decreases the electrophilicity of the Pro carbonyl.^40,41^ Thus, the NCL with preformed Pro phenyl α-thioesters at mM concentrations requires ∼ 24 h to achieve conversions over 90%. Remarkably, with Pro-CO­(SePh), the ligation time is reduced to 2 h with an associated *k*_1_ = (0.72 ± 0.03) M^–1^s^–1^.^4,19^ Using Pro-CO­(SeESNa), a ∼ 90% conversion is achieved in 7 h, resulting in a *k*_1_ = (0.060 ± 0.009) M^–1^s^–1^ (Table 2, entry 2). To improve the ligation rate, the trans-selenoesterification between Pro-CO­(SeESNa) and phenylselenol (DPDS 50 mM + TCEP 100 mM) reduces the ligation time to 4 h (98% conversion, *k*_obs_ = (0.16 ± 0.01) M^–1^s^–1^), giving a rate increment of 2.7-fold (Figures S59, S60, and Table S14). Interestingly, no branched peptide is formed, indicating that in this case the branching constant with Pro-CO­(SePh) is, at least, 10-fold smaller than the NCL *k*_1_ constant (0.72 M^–1^s^–1^). This outcome also confirms the low branching product (4%) observed when the ligation is carried out with preformed Pro-CO­(SePh) and 1.7 equiv of Cys peptide.^4^

### Synthesis of the Cardiotoxin A5 from *Naja naja atra*

In view of these positive results, we carried out the chemical synthesis of the *Naja naja atra* cardiotoxin A5 (**CTX5**) to show the full catalytic potential of SeESNa. CTX5 is a P-type cytotoxin with low cytotoxicity and no hemolytic activity, but it induces vesicle aggregation and binds to the integrin α5β3. The mature protein, a 62-mer, adopts a three-finger tertiary structure comprised of 5 antiparallel β-sheets.^42,43^ This protein contains eight Cys residues and represents a synthetic challenge due to the possible formation of thiolactones and branched thioesters during the ligation between fragments. For the synthesis, we split the protein into three segments: **F1** (Leu^1^–Leu^21^), **F2** (Cys^22^–Gly^39^), and **F3** (Cys^40^–Asn^62^), and designed a sequential assembling strategy: first F1 + F2, and then F1–F2 + F3 (Scheme 3). **F1** was assembled on a 3,4-Diaminobenzamide-Rink resin (Dbz-CONH-resin) following reported protocols.^8^ After chain elongation, acylation of the Dbz with *p*-nitrophenylchloroformate in DCM, followed by DIEA-induced intramolecular cyclization, led to the F1-CO­(Nbz)-CONH-resin. Then, the concomitant cleavage and side chain deprotection under acidolytic conditions afforded **F1-CO­(Nbz)-CONH**_**2**_ (30% yield of the purified peptide). The middle segment **F2-CO­(Dbz)** was designed to incorporate a Dbz moiety at the C-terminus, allowing the ligation with F3. The synthesis of **F2-CO­(Dbz)** was carried out on a 4-(4-(Aminomethyl)-3,5-dimethoxyphenoxy)­butanamide-resin (PAL-resin) according to a recently described strategy: nucleophilic aromatic substitution of 2-fluoronitrobenzene on PAL, followed by the nitroarene reduction with sodium dithionite, provided the Dbz-PAL-resin.^44^ Following stepwise elongation, TFA-mediated cleavage afforded the desired **F2-CO­(Dbz)** fragment (39% yield, purified product). Although the linear synthesis did not generate special problems, the acidic cleavage conditions favored the benzimidazole formation, a side product with a retention time similar to the desired F2-CO­(Dbz) segment.^45^ Hence, HPLC purification with a smooth gradient (5 – > 40% CH_3_CN in 85 min) was necessary to isolate the Dbz peptide and minimize the ligation of this substrate with the F1-CO­(Nbz)-CONH_2_ fragment. The third sequence (**F3**) was assembled on a 4-(4-Hydroxymethyl-3-methoxyphenoxy)-butanamide-ChemMatrix resin (HMPB-ChemMatrix, 6% yield, purified product).

**Scheme 3:**
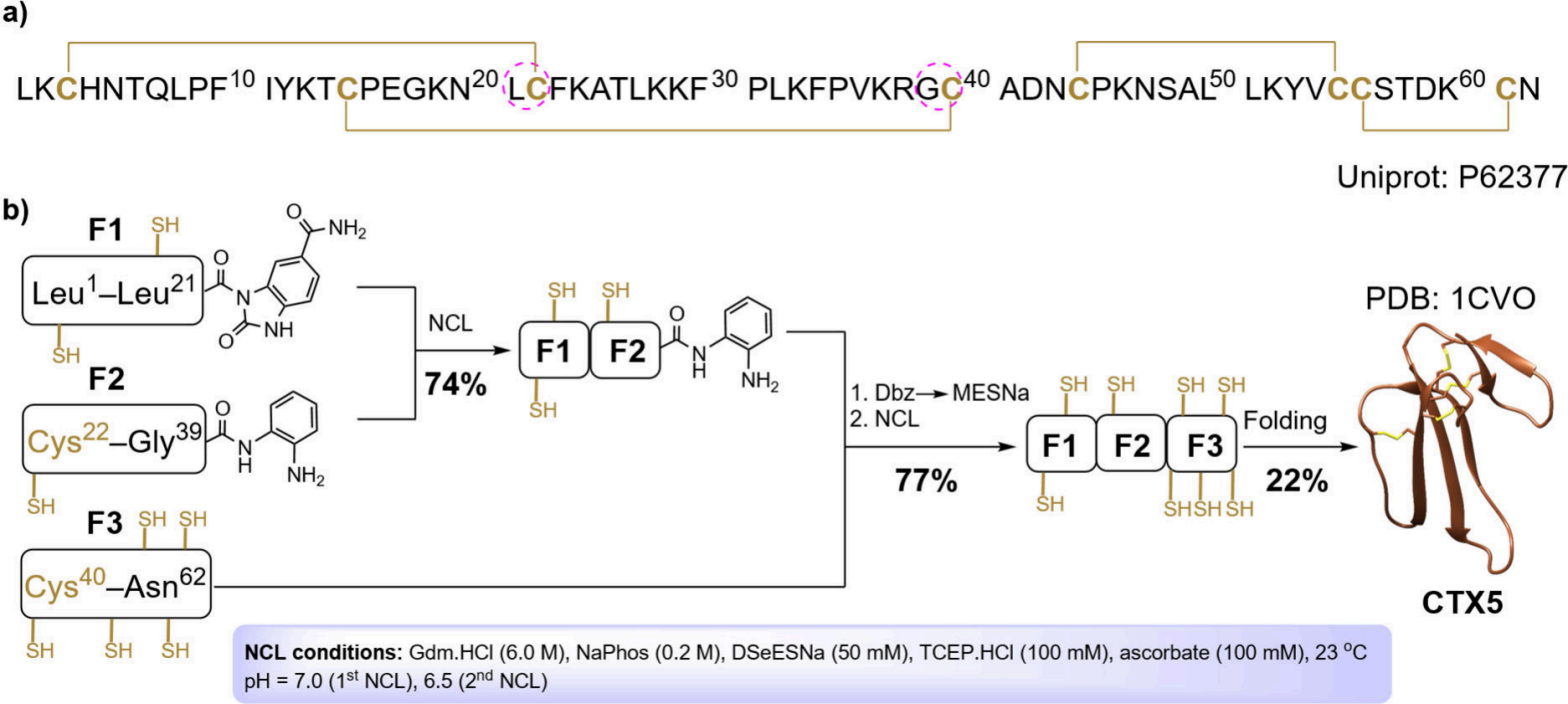
(A) Sequence and Disulfide Alignment of **CTX5** (Dashed Circles Indicate Ligation Points); (B) **CTX5** Synthetic Diagram

The NCL between **F1-CO­(Nbz)-CONH**_**2**_ (26.3 mg, 1.0 × 10^–2^ mmol) and **F2-CO­(Dbz)** (26.3 mg, 1.2 × 10^–2^ mmol) was performed under denaturing conditions (Gdm.HCl 6.0 M, NaPhos 0.2 M, pH = 7.0, 2.0 mL) containing TCEP (100 mM), DSeESNa (50 mM), and ascorbate (100 mM). The ligation achieved >95% completion in 5 h (Figure 6a and b), affording the ligated peptide **F1–F2-CO­(Dbz)** in good yield (34.2 mg, 74%). The short HPLC retention time is a substantial advantage of SeESNa (<5 min), and the good separation from peptides simplifies the purification step by RP-HPLC.

**Figure 6:**
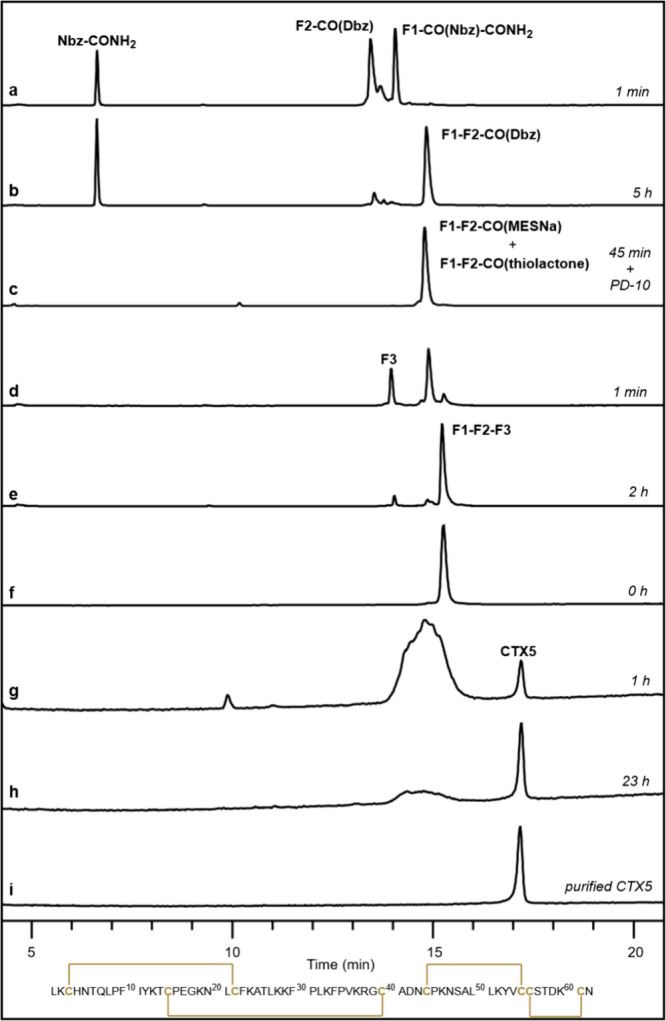
Representative HPLC chromatograms (λ = 220 nm) of the **CTX5** synthesis. (A,B) F1-CO­(Nbz)-CONH_2_ + F2-CO­(Dbz) → F1–F2-CO­(Dbz). (C) F1–F2-CO­(Dbz) → F1–F2-CO­(MESNa+thiolactone). (D,E) F1–F2-CO­(MESNa+thiolactone) + F3 → F1–F2–F3. (F–I) folding **CTX5**.

Preliminary experiments to conduct the second NCL in a one-pot reaction led to undesired high F1–F2 hydrolysis levels (∼ 20–30%). This elevated hydrolysis likely happens during the F1–F2-CO­(Bt) exchange with SeESNa, since incubation of the analogous F1–F2-CO­(MESNa) peptide with SeESNa at pH = 6.3 did not yield any hydrolysis product (Figure S70). Therefore, we reconsidered the ligation strategy between F1–F2-CO­(Dbz) and F3 in three stages: initial Dbz oxidation followed by *in situ* thioesterification with MESNa, then crude desalting and lyophilization, and finally NCL with F3. This approach would also have a single HPLC purification step of the final product. Thereby, **F1–F2-CO­(Dbz)** (26.0 mg, 5.6 × 10^–3^ mmol) underwent oxidation with NaNO_2(aq)_ (20 mM) at −10 °C in denaturing Gdm–NaPhos buffer (1.5 mL, pH = 3.5). The complete oxidation to F1–F2-CO­(Bt) was achieved in 20 min. Next, a solution of MESNa (61.5 mg, 0.37 mmol) in Gdm–NaPhos buffer (1.0 mL, pH = 6.7) was added, and the pH of the resulting solution was carefully adjusted to 6.4 with NaOH_(aq)_ (1 M). The mixture was incubated at room temperature for 45 min. Then, the reaction mixture was desalted (buffer exchanged with H_2_O/TFA 0.1%) using size exclusion (5 kDa, PD-10 column), and freeze-dried (Figure 6c). The mass analysis of the resulting product revealed the presence of a mixture containing **F1–F2-CO­(MESNa)** and a thiolactone, indicating the high propensity to form internal thioesters (Figure 7b).

**Figure 7:**
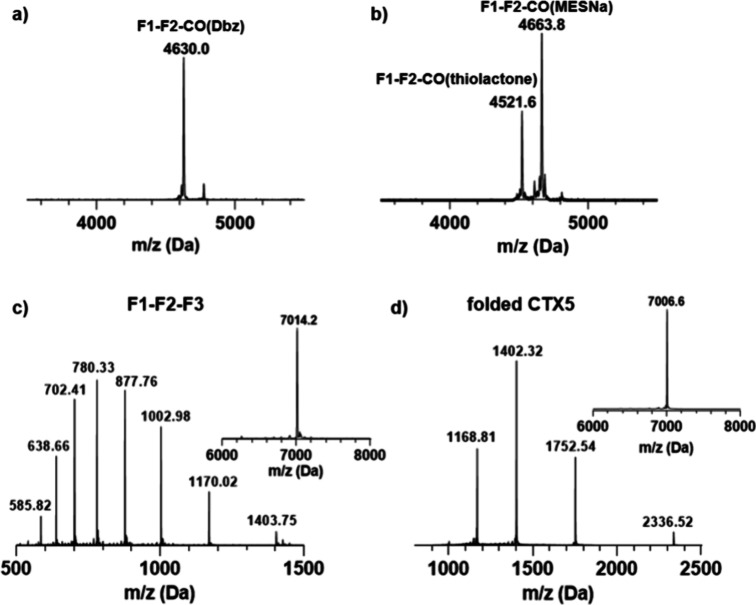
(A,B) MALDI-TOF mass spectra of the synthetic intermediates. (C,D) ESIMS-qTOF of reduced and folded **CTX5**. Inserted in (C) and (D) are the deconvoluted MS of F1–F2–F3 and **CTX5**, respectively.

**F1–F2-CO­(MESNa+thiolactone)** (20.0 mg, 4.3 × 10^–3^ mmol) was ligated to **F3** (12.2 mg, 4.9 × 10^–3^ mmol) at pH = 6.5 using catalysis with SeESNa (DSeESNa 50 mM + TCEP 100 mM) in analogy to the first ligation. The F1–F2 fragment was consumed in 2 h (Figure 6d and e). Remarkably, neither branched nor thiolactone products were detected at the end of the reaction, confirming that SeESNa is an efficient catalyst in selenol-thioester exchange reactions. Following the purification by HPLC, the linear **F1–F2–F3** product was obtained in good yield (23.4 mg, 77%).

The folding of the linear unprotected F1–F2–F3 sequence to form the correct disulfide connectivity required some troubleshooting due to the large number of possible disulfide combinations (764, counting the mixture of disulfide and free Cys).^46^ Our best protocol resulted in a protein concentration of 0.5 mg/mL diluted in a buffer system composed of Tris (0.1 M, pH = 8.0), NaCl (0.15 M), reduced glutathione (2 mM), and oxidized glutathione (0.5 mM). A single peak, more hydrophobic than the linear species, appeared in the HPLC chromatogram under these folding conditions. The folding reached equilibrium (∼50%) within 24 h, and no significant improvement was observed beyond this time (Figure 6f–i). The folding and purification were more efficient at a 5 mg scale (1.1 mg folded **CTX5**, 22%) in our hands. LC-MS confirmed the expected mass corresponding to the formation of four disulfide bonds (calculated [M]^+^: 7006.4, observed: 7006.6, Figure 7d).

The mono and bidimensional homonuclear NMR spectroscopy confirmed the presence of an ordered 3D structure, recognized by the sharp and dispersed signals in the H^N^ region (8.0 – 10.0 ppm). There is also a distinctive NOE signal pattern corresponding to the coupling between H^α^ (3.5 – 5.5 ppm) in the residue (i) and H^N^ in the residue (i+1), between H^N^ (i and i + 1), and NOEs between H^N^ and H^aliphatic^ (Figure 8).

**Figure 8:**
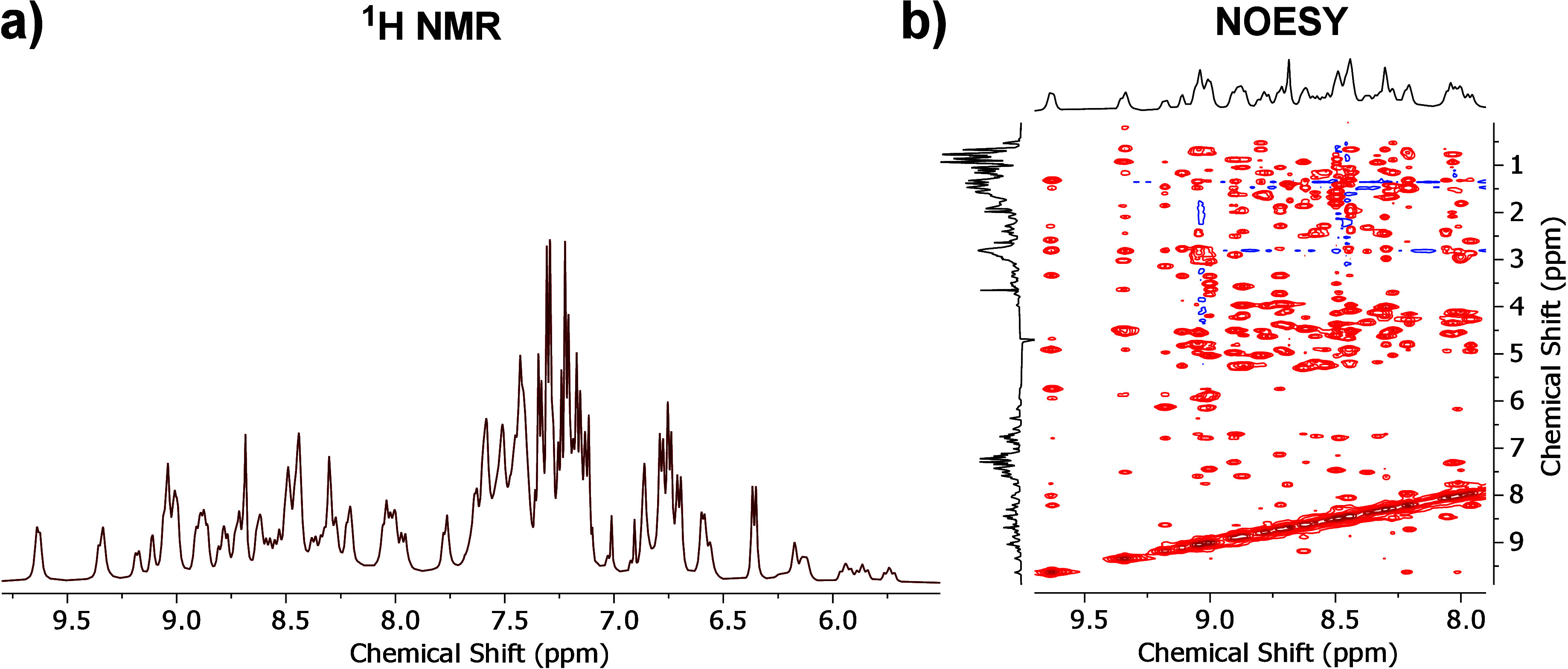
(A) **CTX5** ^1^H NMR corresponding to the −CONH- (8.0 – 10.0 ppm) and aromatic regions. (B) NOESY experiment showcasing the NOE couplings between H^α^ (residue i, 3.5 – 5.5 ppm) and H^N^ (residue i + 1), between H^N^ (i and i + 1), and between H^N^ and H^aliphatic^.

### Expressed Protein Ligation with His_6_–SUMO–Shh^1–159^–GyrA

EPL is an important extension of NCL.^25–27^ This method generates a fusion between a target protein and the N-terminal Cys of a mutated intein, typically GyrA from *Mycobacterium xenopi*.^47,48^ The engineered construct is recombinantly expressed and, under native conditions, undergoes an *N-to-S* acyl shift in the junction site. This thioester, in equilibrium with the amide bond, is trapped in the presence of an exogenous thiol (usually MESNa), resulting in an expressed protein α-thioester. The resulting protein α-thioester is ligated to another protein or peptide, either in the presence of the same thiol (MESNa) or with catalysis mediated by phenyl thiols. Nevertheless, alkyl α-thioesters exhibit poor ligation kinetics, and to date, catalysis with phenyl thiols has been performed mainly under denaturing conditions, which entails an additional folding step and a subsequent yield loss. The use of protein α-selenoesters is not extended because phenylselenol has not been shown to attack intein thioesters, likely due to a lack of nucleophilicity. Alternatively, they are prepared through intein hydrazinolysis followed by phenyl α-selenoesterification via acyl-pyrazole under denaturing conditions, in a two-step reaction.^49^ Consequently, it would be interesting to have a straightforward methodology available that combines intein-mediated selenoesterification and ligation under native conditions, with kinetics similar to those of phenyl α-thioesters.

We first conducted preliminary experiments using a **His**_**6**_ **–SUMO2­(ΔG)–GyrA–CBD** construct to figure out if SeESNa could promote EPL in a single step. SUMO2­(ΔG) is a small protein (92 residues) labeled with a His_6_ tag at the N-terminal side and its C-terminal Gly fused to GyrA–CBD (CBD = chitin binding domain).^50^ Pleasantly, when the **His**_**6**_ **–SUMO2­(ΔG)–GyrA–CBD** construct (10 μM) was incubated with **CTAFS** (**2**, 0.8 mM) under native buffer conditions (pH = 7.8) in the presence of DSeESNa (50 mM), TCEP (100 mM) and ascorbate (100 mM), the ligation product was formed under 1 h with a conversion >95% at room temperature, as estimated by the intensity of the gel bands (Figure S75). This clearly contrasted with the same ligation in the presence of MESNa (100 mM), which showed a much lower ligated fraction (<50%) at the same time (Figure S75).

Encouraged by these results, we next turned our interest to a more challenging protein: Sonic Hedgehog. The N-terminal domain of Sonic Hedgehog (Shh) is a signaling molecule involved in the spatiotemporal control of organogenesis during embryonic development. In adult individuals, Shh signaling misregulation often promotes the onset and growth of tumors such as glioblastoma, basal cell carcinoma, and pancreatic adenocarcinoma.^51,52^ Despite its significance in cancer and the pharmaceutical interest, there are no Shh-targeting drugs clinically available. Therefore, it would be interesting to have a fast and efficient synthetic access to milligram amounts of the protein site-specifically modified with different tags for biophysical and drug discovery studies.

From a structural point of view, Shh is comprised of 174 residues and functionalized at the N-terminal and C-terminal regions by the covalent attachment of palmitic and cholesterol lipids, respectively.^53,54^ We leveraged the internal His^159^-Cys^160^ junction for our semisynthetic approach, and designed a construct with the intein fused at that residue: **His**_**6**_ **–SUMO–Shh** ^**1–159**^ **–GyrA**. A His_6_–SUMO tag was attached to the N-terminal side to increase the expression levels and facilitate its purification.

The inducible plasmid was expressed in *E. coli* Rossetta (DE3) cells. After optimization of the expression conditions (37 °C, 2 h), good expression levels were obtained (29.4 mg/L of protein stock quantified by UV after purification by immobilized metal affinity chromatography – IMAC), but a side product corresponding to the hydrolyzed protein (His_6_–SUMO–Shh^1–159^–COOH) was still detected (Figure S78). This indicates the high lability of the His-Cys bond and the challenge to overcome the competing undesired hydrolysis versus ligation. Next, we synthesized two sequences corresponding to the Shh C-terminal part, incorporating two different tags: **Cys** ^**160**^ **–Gly** ^**174**^ **–PEG–His**_**6**_ and **Cys** ^**160**^ **–Gly** ^**174**^ **–K­(PEG)–Biotin** (Figures S23 and S24). The two peptides were ligated under folding conditions: Tris (10 mM, pH = 6.5), NaCl (100 mM), DSeESNa (25 mM), TCEP (50 mM), ascorbate (100 mM), [His_6_–SUMO–Shh^1–159^–GyrA] = 50 μM, [peptide] = 1.3 mM, 23 °C (Figure S81). The EPL reactions were monitored by SDS-PAGE, and showed >90% conversion at 4 h (34% yield following IMAC purification, calculated based on UV quantification and accounting also for the residual His_6_–SUMO–Shh^1–159^–COOH, Figure 9b). The high-resolution mass spectrometry confirmed the identity of the ligated products (Figure 9c and Figure S82). Attempts to purify the desired protein by FPLC provided low recovery yields of the pure product due to the similar molecular weight of the hydrolyzed protein, which resulted in poorly resolved peaks. Nevertheless, the C-terminal biotin tag would allow the capture and immobilization of the protein for subsequent studies, thus removing the hydrolysis product.

**Figure 9:**
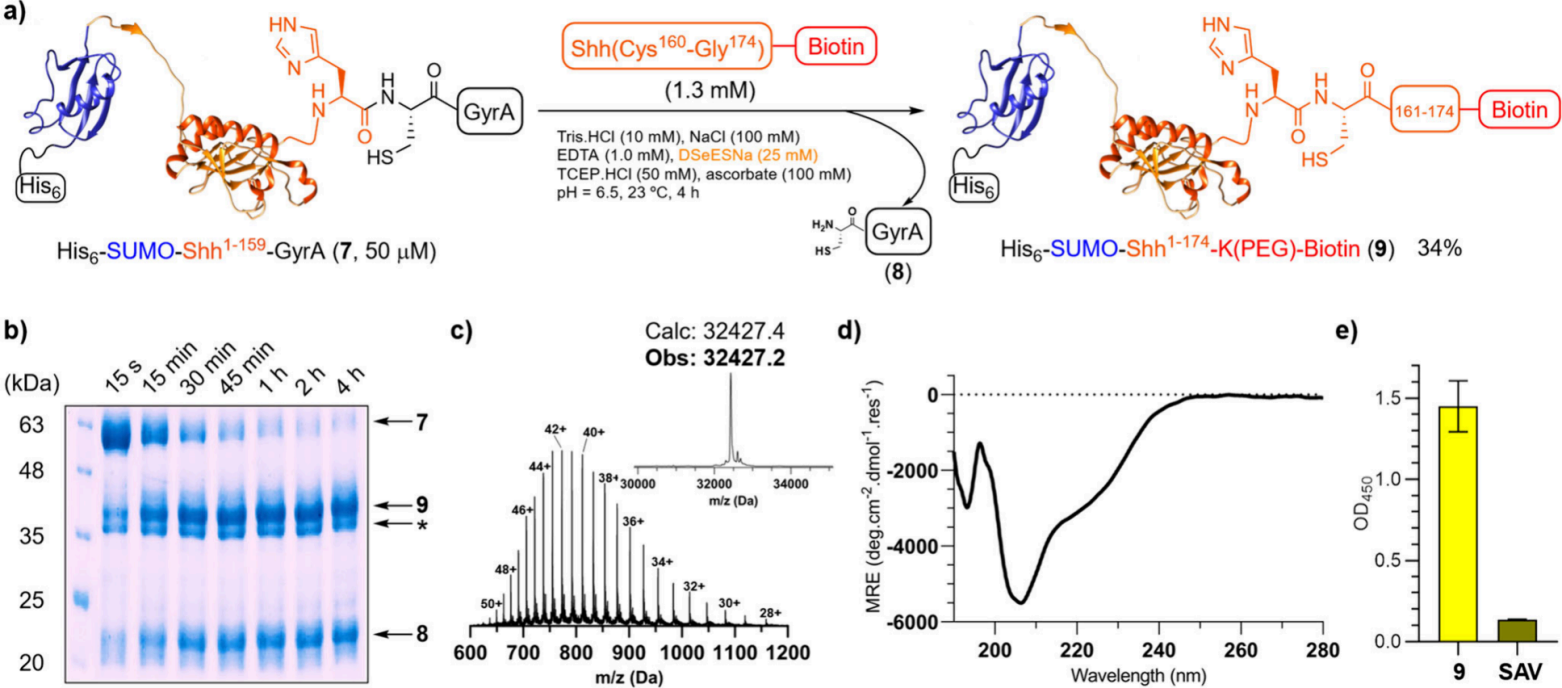
(A) Synthetic scheme of the EPL between **His**_**6**_ **–SUMO–** ^**1–159**^ **Shh–GyrA** and **Cys** ^**160**^ **–Gly** ^**174**^ **–K­(PEG)–Biotin** catalyzed by SeESNa. (B) SDS-PAGE of the EPL at different reaction times. (C) ESIMS of **His**_**6**_ **–SUMO–Shh** ^**1–174**^ **–K­(PEG)–Biotin**. (D) Circular dichroism of **His**_**6**_ **–SUMO–Shh** ^**1–174**^ **–K­(PEG)–Biotin**. (E) ELISA of **His**_**6**_ **–SUMO–Shh** ^**1–174**^ **–K­(PEG)–Biotin** with monoclonal antibody 5E1. SAV = streptavidin. * = His_6_–SUMO–Shh^1–159^–COOH.

Next, circular dichroism (CD) and ELISA assays were carried out with **His**_**6**_ **–SUMO–Shh** ^**1–174**^ **–K­(PEG)–Biotin** to show that the proteins preserved the correct 3D conformation and were fully functional. The CD displayed a negative band at 208 nm, indicative of a helical conformation (Figure 9d), and in agreement with the CD previously reported.^55^ In the ELISA experiments, the plate wells were first coated with streptavidin (SAV, 5 μg/mL). Then, a fraction of the plates was incubated with His_6_–SUMO–Shh^1–174^–K­(PEG)–Biotin (100 nM). After washing away the unbound protein, the monoclonal antibody 5E1 (50 nM), which specifically recognizes the folded state of Sonic,^56,57^ was added to all the wells. Following the addition of the secondary antibody, the signal intensity was measured at 450 nm. The wells containing His_6_–SUMO–Shh^1–174^–K­(PEG)–Biotin gave a signal 11-fold larger than the streptavidin control, meaning that SUMO does not interfere with Shh recognition by 5E1, likely due to the flexibility of the N-terminal region (Figure 9e). Overall, the CD and ELISA results indicate a well-folded structure and, importantly, the ligation conditions in the presence of SeESNa do not affect the protein structure and its recognition by the 5E1 antibody.

Finally, we also carried out a parallel ligation under the same experimental conditions to compare the relative intein cleavage and ligation rates of SeESNa and two representative thiols: MESNa and 4-MPAA. The experiment confirmed all previous results and demonstrated that at 4 h, the relative intein cleavage and ligation rates are higher for SeESNa compared to MESNa and 4-MPAA (Figure S87).

## Conclusions

In summary, here we report SeESNa as a new alkylselenol catalyst for NCL. Its performance is superior to standard phenyl thiols, such as 4-MPAA and 4-MBA. It offers specific advantages in comparison with the established phenylselenol despite its lower acylation strength. Specifically, SeESNa is a better catalyst for NCL due to its higher nucleophilicity. Thereby, it also avoids the accumulation of less reactive thioesters, such as branched thioesters, and circumvents the necessity to add an excess of the Cys peptide. The use of SeESNa in NCL, followed by oxidative protein folding under nondenaturing conditions, is anticipated because it has been shown that other alkyl selenols can increase the rate of disulfide formation compared to the analogous thiol compounds. In the long term, this could accelerate the chemical synthesis of proteins.^58–61^

The results included in this work represent a significant advance in the field of protein semisynthesis. Thus, the use of SeESNa enables EPL under native conditions, resulting in higher yields and shorter reaction times. The superior behavior of this reagent relies on its high-water solubility, its properties as a good leaving group, and its ability to promote seleno-thioester exchange reactions faster than standard phenyl thiols, such as 4-MPAA. We also anticipate that other alkyl selenols with a p*K*_a_ ∼ 6–7 could be used to catalyze EPL upon selenol-thioester exchange. All in all, SeESNa is a valuable contribution to the currently existing toolbox for protein ligation. We are convinced that with its high reactivity, it holds great promise to become a powerful asset for the synthesis and semisynthesis of proteins.

## Experimental Section

### RP-HPLC Analysis

Peptide ligations were monitored by analytical HPLC using an Agilent 1100 system furnished with a quaternary pump and hyphenated to a photodiode array detector. HPLC traces were registered at 220 and 280 nm. Samples were injected into different C18 columns (see Supporting Information for complete column and gradient details). The chromatograms were analyzed using Agilent OpenLab software and processed with GraphPad Prism (8.0).

### RP-HPLC Purification

The semipreparative HPLC was performed in a Waters system furnished with a 1525 binary pump and a Waters 2489 UV/Visible detector (UV monitoring at 220 and 280 nm). The separations were carried out with the following columns:

- XBridge Prep BEH130 C18 OBD (5 μm, 19 × 50 mm). Flow rate 10 mL/min, and a linear gradient of CH_3_CN (with 0.05% TFA) over H_2_O (with 0.1% TFA): 5% to 70% in 85 min.
- Kromasil 100 C18 (5 μm, 10 × 250 mm). Flow rate 4 mL/min, and a linear gradient of CH_3_CN (with 0.05% TFA) over H_2_O (with 0.1% TFA): 5% to 40% in 85 min. This gradient was used to purify the **F2-CO­(Dbz)** fragment of **CTX5**.

### Elemental Analysis

The C, H, S analysis was performed in an Organic Elemental Analyzer (Flash 2000) from Thermo Scientific. Na determination was carried out by ICP-OES in a PerkinElmer Optima 8300 instrument.

### Mass Spectrometry

The MALDI-TOF peptide mass analysis was performed in a Bruker Daltonics Autoflex III Smartbeam. The Spectra were registered in the reflector positive or negative ion mode: 1 μL of α-cyano-4-hydroxycinnamic acid (10 mg/mL dissolved in H_2_O/CH_3_CN with 0.05% TFA) and 1 μL of the peptide sample (dissolved in the same solution as the matrix) were mixed in the plate and dried. The spectra were analyzed using Bruker flexAnalysis (3.4) and processed with GraphPad Prism (8.0).

The LC-MS intact protein mass analysis was carried out in the following instruments:

- UltimateTM 3000 RS UPLC (Thermo Fisher Scientific GmbH) hyphenated to a maXis II-qTOF (Bruker Daltonik GmbH). Acquisition parameters: capillary voltage of 4500 V, end plate offset of 500 V, nebulizer at 3.5 bar, dry heater at 200 °C, and dry gas at 480 L/h. The data were analyzed with DataAnalysis (5.3, Bruker) and deconvoluted using the MaxEnt algorithm implemented in the software.
- UPLC Acquity Premier (Waters) hyphenated to an Acquity Premier PDA detector (Waters), and a Select Series Cyclic IMS-qTOF mass spectrometer (Waters) with an ESI source. The samples were loaded on an Acquity UPLC BEH300 C18 column (1.7 μm, 2.1 mm × 100 mm, Waters) at a flow rate of 0.3 mL/min, and eluted with a linear gradient of CH_3_CN (with 0.1% formic acid) over H_2_O (with 0.1% formic acid): 10% to 100% in 8 min. MS operating parameters were: capillary voltage = 3000 V, nebulizer = 6.0 bar, desolvation gas = 800 L/h, desolvation temperature = 250 °C, mass range = 500 – 4000 Da. The MS spectra were analyzed with Masslynx (4.2) and deconvoluted using UniDec (7.0.2).^62^

### ^1^H NMR Quantification of Compound Content

The net peptide and DSeESNa content were determined by ^1^H NMR in a Bruker Ascend (9.4 T, 400 MHz) furnished with a room temperature iProbe. Sodium acetate (98.5%) was used as an internal reference. The samples were dissolved in D_2_O (0.7 mL) and the experiments registered using the following parameters: d_1_ = 30 s, spectral window = 15 – (−5) ppm, scans = 16, acquisition time = 12.5 min. The spectra were analyzed with Bruker TopSpin (4.4.0). The net compound content was calculated by applying the following expression:^63^

$$\mathrm{Net compd}.\mathrm{content}\left(\%\right)=\mathrm{Std}.\mathrm{purity}\left(\%\right)\times \frac{m_{\mathrm{std}}}{m_{\mathrm{sample}}}\times \frac{{\mathrm{MW}}_{\mathrm{sample}}}{{\mathrm{MW}}_{\mathrm{std}}}\times \frac{{\mathrm{nH}}_{\mathrm{std}}}{{\mathrm{nH}}_{\mathrm{sample}}}\times \frac{{\mathrm{Area}}_{\mathrm{sample}}}{{\mathrm{Area}}_{\mathrm{std}}}$$

where:

- **Std.**_**purity**_ is the standard purity (sodium acetate).
- **m**_**std**_ and **m**_**sample**_ are the weights of the standard and the sample, respectively.
- **MW**_**sample**_ and **MW**_**std**_ are the molecular weights of the sample and the standard, respectively.
- **nH**_**std**_ and **nH**_**sample**_ are the number of H nuclei in a given signal of the standard (3H) and the sample, respectively.
- **Area**_**sample**_ and **Area**_**std**_ are the integrals of the H nuclei belonging to the selected signals of the sample and the standard, respectively.

The calculated net compound content was: **LYRAV-CO­(SeESNa)** (78%), **LYRAV-CO­(4-MPAA)** (67%), **LYRAV-CO­(MESNa)** (73%), **LYRAV-CO­(Nbz)-G** (54%), **LYRAA-CO­(SeESNa)** (74%), **LYRAP-CO­(SeESNa)** (77%), **LYRAS-CO­(SeESNa)** (77%), **CTAFS** (82.5%), **LYRAVCTAFS** (73.0%), **DSeESNa** (C_4_H_8_S_2_O_6_Se_2_Na_2_, 75%).

### NMR of Folded CTX5

The ^1^H NMR and NOESY experiments were performed in a Bruker AVANCEIIIHD (11.7 T, 500 MHz) furnished with a Cryoprobe. The experiments were registered at 300 K in a Shigemi NMR microtube with H_2_O suppression. The oxidized **CTX5** (4.5 mg, 6.4 × 10^–4^ mmol) was dissolved in H_2_O/D_2_O (9:1, 1.0 mL, pH (not corrected) = 3.0). The solution was spun out, and 0.5 mL was loaded into the Shigemi microtube. Acquisition parameters for ^1^H NMR: number of scans = 512, spectral width = 16.0 ppm, acquisition time = 2.045 s/scan. Acquisition parameters for NOESY: number of scans = 48, spectral width (f2, f1) = 16.0 ppm, acquisition time (f2) = 0.13 s/scan, acquisition time (f1) = 0.050 s/scan.

### Circular Dichroism

The CD analysis of **His**_**6**_ **–SUMO–ShhN** ^**1–174**^ **–K­(PEG)–Biotin** was performed in a Jasco J-1500 CD spectropolarimeter (Jasco Inc.) in a 1 mm path length quartz cuvette. [**protein**] = 12.5 μM, dissolved in a STE*:glycerol (9:1) buffer (pH = 6.5), temperature = 20 °C. Acquisition settings were: range = 320 – 190 nm, data pitch = 0.1 nm, integration time = 4 s, scanning speed = 100 nm/min, bandwidth = 1.0 nm, accumulations = 4. The data were acquired in mdeg with Jasco Spectra Manager (2.0), and smoothed with the binomial function implemented in the package. Then, it was converted to mean residue ellipticity (MRE) using the following expression:

$${\left[\theta \right]}_{R}{=m}^{o}\times \frac{\mathrm{MRW}}{10\times C\times l}$$

where:

- **[θ]**_**R**_ is MRE in deg·cm^2^·dmol^–1^·residue^–1^
- **m°** is the CD measurement in mdeg.
- **MRW** is the mean residue weight, obtained by dividing the protein molecular weight by the number of residues.
- **C** is the protein concentration (g/L).
- **l** is the path length (cm).

*STE buffer: Tris (10 mM), NaCl (100 mM), EDTA (1.0 mM), pH = 6.5.

### pH Measurements and Considerations

The pH was measured with a sensiON pH31 (±0.01) furnished with a Hach 52 09 probe (calibrated with standard Hach buffers: 7.00, 4.01, 9.21), and a Crison 506 (±0.01) with a Hach 52 08 probe (calibrated with standard Hach buffers: 7.00, 4.01). The pH measurements reported here, as in previous work (reference 4), are uncorrected: they reflect the ‘pH readings (pH_r_)’, but not the ‘true pH (pH*)’. A correction factor should be applied that accounts for the ionic strength. If the pH_r_ is carried out using a glass diaphragm, this value is ∼ + 0.5 units when the [Gdm.HCl] = 5.0 M (in H_2_O).^64^

$${\mathrm{pH}}^{*}={\mathrm{pH}}_{r}+{\delta \mathrm{pH}}^{*}$$

where:

$${\delta \mathrm{pH}}^{*}=-0.182{\left[\mathrm{Gdm.HCl}\right]}^{1/2}+0.161\left[\mathrm{Gdm.HCl}\right]+0.0055{\left[\mathrm{Gdm.HCl}\right]}^{2}$$

The Hach 52 09 and Hach 52 08 have both ceramic diaphragms. Although there are no reported correction factors for these types of electrodes in phosphate buffer, a comparison of glass and ceramic using the same guanidine solutions (5.0 M Gdm.HCl, 0.17 M NaPhos) displays similar pH readings. Therefore, we estimate that the ‘true pH’ is around +0.5 units higher than the measured pH_r_, and the pH range of the kinetic experiments (5.0 M Gdm.HCl, 0.17 M NaPhos) is ∼7.4–7.5.

### Synthesis of DSeESNa


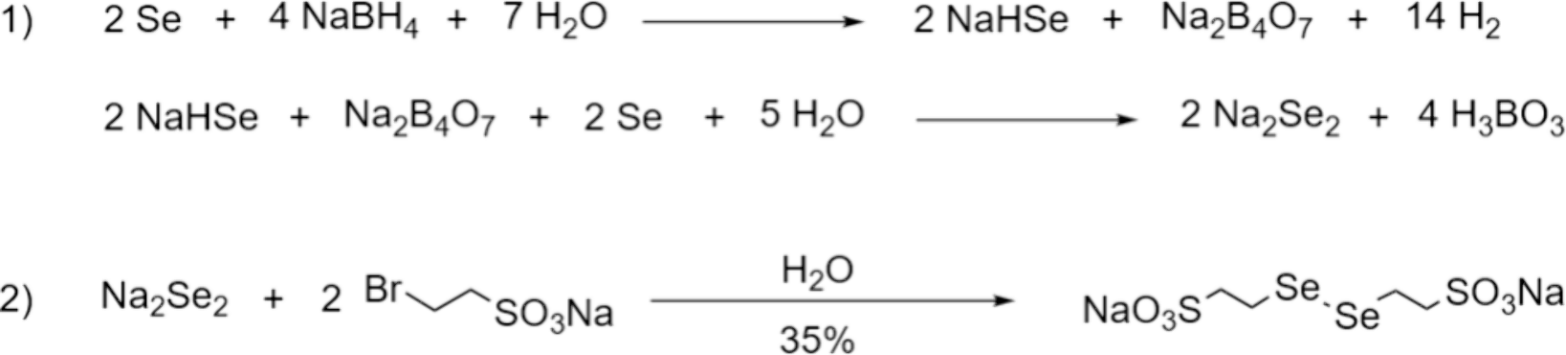


In a three-neck round-bottom flask flushed with N_2_ and equipped with a dropping funnel and a Teflon magnetic stirring bar, Se (5.36 g, 0.068 mol) was mixed with degassed H_2_O (25 mL). Then, a solution of NaBH_4_ (5.15 g, 0.136 mol) in degassed H_2_O (20 mL) was added dropwise at room temperature under constant stirring (30 min). After the mixture became homogeneous, additional Se (5.36 g) was added in small portions (30 min).^29^

To the resulting red solution, sodium 2-bromoethanesulfonate (10 g, 0.047 mol) dissolved in degassed H_2_O (20 mL) was added dropwise at room temperature (30 min). The mixture was stirred at room temperature for 4 h. Next, the excess of Na_2_Se_2_ was removed by quenching the reaction with HCl_(aq)_ (18%, 50 mL, dropwise addition), and the evolved H_2_Se was neutralized in a bleach trap connected to the flask. The solution was filtered over Celite and freeze-dried. The crude was purified by low-pressure flash reverse phase chromatography (Biotage Isolera One system, RediSepRf Gold 50 g HP C18 cartridge, 20 mL/min) using an isocratic gradient (10 min with 100% H_2_O, then 5 min with 100% CH_3_CN). The fractions containing the pure compound (analyzed by HPLC) were pooled together and lyophilized. The product was obtained as a fluffy yellow powder (4.60 g, 35%).

^**1**^H NMR (400 MHz, D_2_O) δ (ppm): 3.24 (m, 4H), 3.35 (m, 4H). ^**13**^C NMR (400 MHz, DMSO-d_6_) δ (ppm): 24.3, 53.2. **IR** (film) ν (cm^–1^): 559.3 (w, ν C–Se), 582.4 (w), 651.9 (w), 721.3 (m, ν Se–Se), 790.7 (w, ν C–SO_3_), 1047.2 (s, ν_s_ SO_3_), 1178.4 (s, ν_as_ SO_3_), 1188.0 (s, ν_as_ SO_3_). **Melting point** (°C): 280–282 (decomposition). Calculated **elemental analysis** for C_4_H_8_S_2_Se_2_O_6_Na_2_·(H_2_O)_2_·(H_3_BO_3_): C (9.27%), H (2.92%), O (33.97%), S (12.38%), Se (30.49%), Na (8.88%), B (2.09%). Found: C (9.3%), H (2.6%), S (12.2%), Na (8.5%).

### Synthesis of LYRAXaa-CO­(SeESNa), LYRAV-CO­(MESNa), and LYRAV-CO­(4-MPAA) Peptides

The synthesis of these model peptides was accomplished from LYRAXaa-CO­(Dbz) precursors that were prepared following reported protocols.^44^ These are described in detail for the synthesis of CTX5 F2-CO­(Dbz) fragment (see below). The following experimental procedure illustrates the synthesis of LYRAV-CO­(SeESNa), which is similar for other LYRAXaa-CO­(SeESNa) peptides.

#### LYRAV-CO­(SeESNa)

LYRAV-CO­(Dbz) (70.0 mg, 9.8 × 10^–2^ mmol) was dissolved in DMF (1 mL) and cooled in an ice bath. Then, AcOH (0.1 mL) and ^*t*^BuONO (0.1 mL, 0.76 mmol) were added, and the solution was incubated for 30 min on ice. Following the oxidation, the peptide was precipitated over cold Et_2_O (40 mL) and collected by centrifugation. The resulting LYRAV-CO­(Bt) pellet was dissolved in Gdm.HCl (6.0 M)–NaPhos (0.2 M) buffer (2 mL, pH = 7.0) containing: DSeESNa (56.0 mg, 0.10 mmol), TCEP.HCl (56.0 mg, 0.20 mmol), ascorbic acid (35.2 mg, 0.20 mmol), and incubated for 30 min at room temperature (reaction pH = 6.6). Following the reaction, the desired product was directly purified by semipreparative HPLC to yield **LYRAV-CO­(SeESNa)** (44.2 mg after lyophilization, 55%). Mass (MALDI-TOF): calculated [M – H]^−^ 790.8, found 791.0.

#### LYRAV-CO­(MESNa)

Oxidation of LYRAV-CO­(Dbz) (20.0 mg, 2.8 × 10^–2^ mmol) to afford LYRAV-CO­(Bt) was carried out as described above. Then, the LYRAV-CO­(Bt) precipitate was dissolved in Gdm.HCl (6.0 M)–NaPhos (0.2 M) buffer (2 mL, pH = 7.0) containing MESNa (41.0 mg, 0.25 mmol), and incubated for 30 min at room temperature. The desired product was purified by semipreparative HPLC (19.0 mg, 90%). Mass (MALDI-TOF): calculated [M + H]^+^ 745.9, found 745.4.

#### LYRAV-CO­(4-MPAA)

LYRAV-CO­(Dbz) (100.0 mg, 0.14 mmol) was dissolved in DMF (2 mL) and cooled in an ice bath. Then, AcOH (0.2 mL) and ^*t*^BuONO (0.2 mL, 1.52 mmol) were added, and the solution was incubated for 30 min on ice. Following the oxidation, the peptide was precipitated over cold Et_2_O (40 mL) and collected by centrifugation. Next, the LYRAV-CO­(Bt) pellet was dissolved in Gdm.HCl (6.0M)–NaPhos (0.2 M) buffer (5 mL, pH = 7.0) containing 4-MPAA (100 mg, 0.59 mmol), TCEP.HCl (55.0 mg, 0.19 mmol), and incubated for 40 min at room temperature. After the thioesterification, the reaction was acidified with TFA until pH = 2.0, and the excess of 4-MPAA was extracted with Et_2_O (3 × 20 mL). The aqueous phase was purified by semipreparative HPLC to give the desired product after the lyophilization (40.0 mg, 37%). Mass (MALDI-TOF): calculated [M + H]^+^ 772.0, found 771.5.

#### Synthesis of LYRAV-CO­(Nbz)-G

It was carried out on a Fmoc-Rink-amide resin (0.74 mmol/g, 0.3 mmol) following reported protocols.^8,65^ Briefly, following the coupling of Gly and Dbz-COOH using standard Fmoc-SPPS, Fmoc-Val-OH (1.0 mmol) was preactivated with HATU (1.0 mmol) and DIEA (1.5 mmol) for 30 s, and then added to the resin. After 30 min of reaction, the resin was filtered and washed away. Then, the stepwise elongation of the sequence afforded Boc-LYRAV-CO­(Dbz)-G-Rink resin. The addition of *p*-nitrophenylchloroformate (1.0 mmol) in CH_2_Cl_2_ (2 mL) and incubation for 30 min, followed by DIEA treatment (0.5 M in DMF, 15 min), induced the Nbz formation and gave the Boc-LYRAV-CO­(Nbz)-G-Rink resin. The TFA-mediated cleavage, workup, and RP-HPLC purification yielded the desired **LYRAV-CO­(Nbz)-G** peptide (86.0 mg, 34%). Mass (MALDI-TOF): calculated [M + H]^+^ 838.0, found 837.7.

### NCL with LYRAXaa-CO­(SeESNa), LYRAV-CO­(4-MPAA), LYRAV-CO­(MESNa), LYRAV-CO­(Nbz)-G, and CTAFS

#### General Procedures

Peptides (quantities between 0.900 mg and 3.200 mg) for the kinetic experiments were weighed in a Mettler Toledo XPR2U microbalance (±0.001 mg). The initial peptide concentrations were calculated according to the weight and the net peptide content measured by ^1^H NMR. Other reagents were weighed in a Sartorius CP225D scale (±0.01 mg). For DSeESNa, a net mass content (C_4_H_8_S_2_Se_2_O_6_Na_2_) of 75% was used to prepare the solutions.

The reactions were carried out at 23 °C under N_2_ atmosphere using degassed solutions. Stock solutions of H-Tyr-OH (20.0 mM in H_2_O) and NaPhos (0.2 M, pH = 7.3) containing Gdm.HCl (6.0 M) were sparged with N_2(g)_ for 15 min.

#### Reactions in the Presence of SeESNa

DSeESNa (17.0 mg, 3.0 × 10^–2^ mmol), TCEP.HCl (17.2 mg, 6.0 × 10^–2^ mmol) and ascorbic acid (10.6 mg, 6.0 × 10^–2^ mmol) were dissolved in the denaturing NaPhos buffer (Gdm.HCL 6.0 M, NaPhos 0.2 M, 0.50 mL). H-Tyr-OH (0.060 mL) was added, and the pH was adjusted to 7.1 using solutions of NaOH_(aq)_ (10 and 1 M). The volume was brought to 0.60 mL with H_2_O to constitute the ligation buffer.

All the ligations were performed following the same protocol that we described here for the reaction shown in Figure 2: **CTAFS** (1.052 mg, 1.64 × 10^–3^ mmol, 2.7 mM) was dissolved in the ligation buffer, and then added to **LYRAV-CO­(SeESNa)** (1.130 mg, 1.08 × 10^–3^ mmol, 1.8 mM). At specific times, aliquots of the ligation (20 μL) were withdrawn and directly injected (5 μL) into RP-HPLC with simultaneous detection at 220 and 280 nm. The reaction vial was flushed with N_2(g)_ after the sample removal. The pH was checked at the end of the reaction (7.0). For other replicates, aliquots (20 μL) were withdrawn and quenched with the same volume of HCl (0.5 M, 8:2 H_2_O/CH_3_CN), and analyzed (5 or 10 μL) by HPLC on the same day.

#### Reactions in the Presence of 4-MPAA

4-MPAA (10.1 mg, 6 × 10^–2^ mmol) and TCEP.HCl (8.60 mg, 3 × 10^–2^ mmol) were dissolved in denaturing NaPhos buffer (0.50 mL). H-Tyr-OH (0.060 mL) was added, and the pH was adjusted to 7.1 using solutions of NaOH_(aq)_ (10 and 1 M). The volume was brought to 0.60 mL with H_2_O to constitute the ligation buffer. Then, **CTAFS** (3.6 – 5.3 mM) was dissolved in the ligation buffer and added to **LYRAV-CO­(4-MPAA)** (1.3 – 2.0 mM). At specific times, aliquots of the ligation (20 μL) were withdrawn and quenched with the same volume of HCl (0.5 M, 8:2 H_2_O/CH_3_CN). Samples were spun out to eliminate any precipitate and analyzed on the same day by RP-HPLC with simultaneous detection at 220 and 280 nm. The reaction vial was flushed with N_2(g)_ after the sample removal. The pH was checked at the end of the reaction (6.9 – 7.0).

### Determination of the Peptide Concentration

The compounds corresponding to the different HPLC chromatographic peaks were collected and analyzed by MALDI-TOF mass spectrometry. The product (LYRAXaaCTAFS, **P**) and the branched thioester (LYRAXaaC­(LYRAXaa­[COS-])­TAFS, **PA**) were quantified (at a given time, t) by integration of the corresponding peak areas at 280 nm, and referenced to the internal signal of H-Tyr-OH ([H-Tyr-OH] = 2.0 mM).

$$\begin{array}{l} {\left[P,\mathrm{PA}\right]}_{t}=\frac{{\mathrm{Area}}_{280\mathrm{nm P},\mathrm{PA}}\times 2.0\mathrm{mM}}{{\mathrm{nTyr}\times \mathrm{Area}}_{280\mathrm{nm Tyr}}}\\ \mathrm{nTyr}=1\left(P\right),2\left(\mathrm{PA}\right) \end{array}$$

The **LYRAXaa-CO­(SeESNa)** and **LYRAV-CO­(MESNa)** concentrations (acyl peptides designated as **A**) were calculated by applying a correction factor (0.52 and 0.97, respectively). These correction factors were estimated from the ratio of the areas (280 nm) of the LYRAXaa-CO­(SeESNa) and LYRAV-CO­(MESNa) peptides at the initial time, and the product of the ligation with cysteamine (0.2 M, pH = 7.0) at time = 2 h (LYRAV-CONH-(CH_2_)_2_SH).

The **LYRAV-CO­(4-MPAA)** and **LYRAV-CO­(Nbz)-G** concentrations were determined through a mass balance:

$${\left[A\right]}_{t}={\left[A\right]}_{o}-\left({\left[P\right]}_{t}+2{\left[\mathrm{PA}\right]}_{t}\right)$$

### Kinetic Simulations and Rate Constant Determination

The rate constant parameters are given as average ± standard deviation of independent replicates. They were calculated by simulating the reaction profiles of the different species (**A**, **P**, **PA**). The simulations were performed with COPASI (4.37 and 4.42) using the kinetic equations corresponding to elementary reaction steps.^32^

The NCL kinetic model includes the following expressions:

Ligation of **A** and **B** (**B** corresponds to peptide **2** in all the model reactions) leads to the formation of the intermediate thioester (**AB**), followed by the *S* → *N* rearrangement.

3 $$A+B\begin{array}{l} k_{1}\\ ⇌\\ k_{-1} \end{array}\mathrm{AB}+\mathrm{RXH}\overset{k_{\mathrm{int}}}{\to }P,\left(R=\mathrm{alkyl},\;\mathrm{aryl};\;X=S,\mathrm{Se}\right)$$

Under the ligation conditions, i.e., < 3 equiv of peptide **B**, the accumulation of the transient thioester **AB** is much smaller than **P** (the estimated highest concentration <7% for the ligation between **1** and **2**, 3 equiv of **2**). Thus, its contribution can be ignored and the kinetic model approximated to

4 $$A+B\overset{k_{obs}}{\to }P+\mathrm{RXH}$$

where *k*_obs_ is the apparent rate constant for the product (**P**) formation. For simplicity, we refer to this constant as *k*_1_.

The equilibrium between **A**, **P**, **PA**, and **RXH**.

5 $$P+A\begin{array}{l} k_{2}\\ ⇌\\ k_{-2} \end{array}PA+\mathrm{RXH}$$

Therefore, the kinetic model is described by combining (4) and (5) to give the following equation 6:

6 $$A+B\overset{k_{1}}{\to }P+A\begin{array}{l} k_{2}\\ ⇌\\ k_{-2} \end{array}\mathrm{PA}+\mathrm{RXH}$$

The experimental values of [**A**]_t_, [**P**]_t_, [**PA**]_t_, [**B**]_o,_ and [**RXH**]_o_ were loaded in the software to perform the simulations. Then, the corresponding rate constants were estimated by applying a Differential Evolution algorithm (Scheme 4).^66^ During the simulation, the [**A**]_o_ (limiting reagent) was variable to adjust it to the mass balance of all the reaction products. This means that, in some cases, the [**A**]_o_ experimental and the simulated can slightly diverge (<5%). We keep in the experimental procedure the [**A**]_o_ empirically calculated, but the [**A**]_o_ shown in the schemes represents the value obtained after the simulation, which exactly corresponds to the total product formation (**P** + **PA**) found in the reaction (**PA** is obtained in the ligations catalyzed by phenylselenol).

**Scheme 4:**
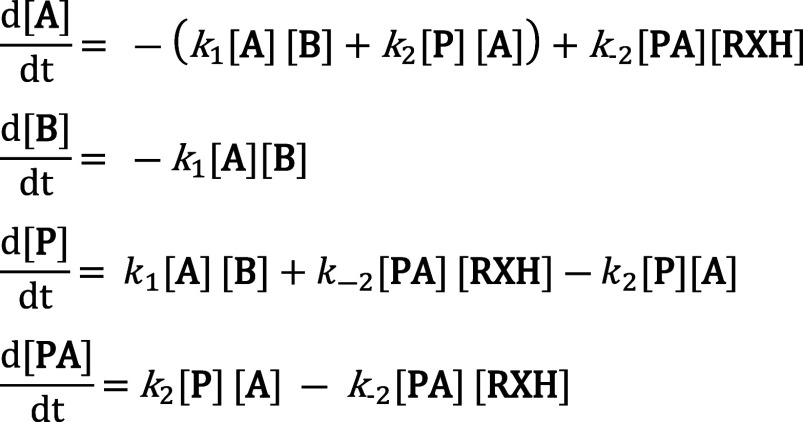
Rate equations describing the kinetic model (6) used in the simulations

Every system was iterated until the obtained parameters remained significantly unchanged and the fitted values corresponding to **A**, **P**, and **PA** had a root-mean-square ≤ 5 × 10^–6^. Each replicate was simulated separately. The experimental and fitting data were plotted using GraphPad Prism (8.0).

### Synthesis of CTX5

#### General Procedure for Fmoc-SPPS

The solid phase peptide synthesis was carried out following standard Fmoc-SPPS protocols: I) coupling in DMF with Fmoc-Xaa–OH (5 equiv with regard to the synthesis scale, ∼ 0.3 – 0.4 M in DMF), HBTU (5 equiv), and DIEA (7.5 equiv). The Fmoc-amino acid was preactivated for 30 s, and then added to the resin. The coupling times were 45 min, and the coupling efficiency was qualitatively monitored through the Kaiser test. II) Fmoc removal with piperidine (20% in DMF) for 5 min.

#### General Procedure for TFA Cleavage and Workup

The resin cleavage was carried out with TFA/H_2_O/TIS/thioanisole (88:2:5:5, 40 mL/1 g peptidyl-resin, 1 h 30 min). The TFA solution containing the fragment was separated from the resin by filtration, using additional TFA washings (3 × 5 mL). The TFA was evaporated by means of N_2(g)_ sparging, the crude residue precipitated over cold Et_2_O (40 mL), and centrifuged (4400 rcf x 5 min). Once the supernatant was discarded, the pellet was redissolved in H_2_O/CH_3_CN (20 mL) and freeze-dried. The resulting residue was purified by semipreparative HPLC.

#### Synthesis of the CTX5 F1-CO­(Nbz)-CONH_2_ Fragment

The synthesis was carried out in a Fmoc-Dbz-CONH-Rink polystyrene resin (0.49 mmol/g, 1.0 mmol). The C-terminal Fmoc-Leu-OH (1.06 g, 3.0 mmol) was coupled for 30 min with HATU (1.14 g, 3.0 mmol) and DIEA (0.78 mL, 4.5 mmol) activation. After the Leu incorporation, the sequence was elongated using standard Fmoc-SPPS protocols. The last residue was introduced as Boc-Leu-OH. Following chain assembly, 1.1 g of resin was swollen in DCM. Then, *p*-nitrophenylchloroformate (0.50 g, 2.50 mmol) in DCM (4 mL) was added, and the mixture was incubated for 30 min. Next, the resin was filtered and washed with DMF. The addition of DIEA (0.5 M in DMF, 10 mL) induced on resin cyclization (15 min). The resulting F1-CO­(Nbz)-CONH-Rink resin was washed with DCM, dried under vacuum, and cleaved following the general protocol. The resulting residue was purified by semipreparative HPLC, affording the desired **F1-CO­(Nbz)-CONH**_**2**_ fragment (140 mg, 30%). Mass (MALDI-TOF): calculated [M + H]^+^ (average isotopes) 2608.1, found 2608.3.

#### Synthesis of the CTX5 F2-CO­(Dbz) Fragment

It was undertaken on a NovaSyn PEG resin (0.1 mmol/g, 0.2 mmol). First, the Fmoc-PAL linker was introduced manually. After removal of the Fmoc, 2-fluoronitrobenzene (1.0 mL, 9.5 mmol) in DMF (4 mL) and DIEA (0.5 mL, 2.9 mmol) were mixed with the resin for 5 h. Next, the resin was filtered, washed with DMF, and a mixture of Na_2_S_2_O_4_/K_2_CO_3_ (1:1, 2.0 × 10^–2^ mol) in H_2_O (40 mL) was added overnight to reduce the nitro group, affording the *o*-aminoaniline-PAL resin. Chain elongation was performed following the Fmoc-SPPS protocol. N-terminal Cys was introduced as Boc-Cys­(Trt)–OH. Following the cleavage and workup, the product was purified by semipreparative HPLC (Kromasil 100 C18, 5 μm, 10 × 250 mm), yielding the desired **F2-CO­(Dbz)** (from 1.9 g of resin, 175 mg of purified peptide, 39%). Mass (MALDI-TOF): calculated [M + H]^+^ (average isotopes) 2199.8, found 2199.9.

#### Synthesis of the CTX5 F3 Fragment

The **F3** synthesis was accomplished on a HMPB-ChemMatrix resin (HMPB = 4-Hydroxymethyl-3-methoxyphenoxybutyric acid, 0.6 mmol/g, 1.0 mmol). The C-terminal Fmoc-Asn­(Trt)–OH (1.49 g, 2.5 mmol) was coupled with DIC/HOAt activation (1:1, 0.39 mL, 0.34 g, 2.5 mmol) and catalytic DMAP (30 mg, 0.25 mmol) in DCM (5 mL) for 2 h. The coupling was repeated with a fresh amino acid solution. The sequence was stepwise elongated similarly to the other two fragments. The cleavage (3.9 g peptidyl-resin) and subsequent workup afforded the crude residue (0.845 g). HPLC purification and lyophilization of the pure fractions yielded the **F3** fragment (95.2 mg, 6%). Mass (MALDI-TOF): calculated [M + H]^+^ (average isotopes) 2494.9, found 2494.9.

### NCL between F1-CO­(Nbz)-CONH_2_ and F2-CO­(Dbz)

**F2-CO­(Dbz)** (26.3 mg, 1.2 × 10^–2^ mmol) was dissolved in Gdm.HCl (6.0 M)–NaPhos buffer (0.2 M, 2.0 mL, pH = 7.0) containing: DSeESNa (50 mM), TCEP.HCl (100 mM) and ascorbic acid (100 mM). The resulting mixture was added to **F1-CO­(Nbz)** (26.3 mg, 1.0 × 10^–2^ mmol), and the reaction was left at room temperature (23 °C) for 5 h. Next, the ligation was directly purified by semipreparative HPLC, affording the desired **F1**–**F2-CO­(Dbz)** fragment after lyophilization (34.2 mg, 74%). Mass (MALDI-TOF): calculated [M + H]^+^ (average isotopes) 4629.7, found 4630.0.

### Thioesterification of F1–F2-CO­(Dbz): F1–F2-CO­(MESNa)

**F1**–**F2-CO­(Dbz)** (26.0 mg, 5.6 × 10^–3^ mmol) was dissolved in Gdm.HCl (6.0 M)–NaPhos buffer (0.2 M, 1.5 mL, pH = 3.5). The solution was cooled in an ice-salt bath (−10 °C), and NaNO_2_ (30 μL of a 1 M stock solution in H_2_O) was added. The mixture was incubated at −10 °C for 20 min. Then, a solution of MESNa (61.5 mg, 0.37 mmol) in Gdm.HCl (6.0 M)–NaPhos buffer (0.2 M, 1.0 mL, pH = 6.7) was cooled in ice and added to the peptide mixture. The pH of the resulting solution (5.4) was carefully adjusted with NaOH_(aq)_ (1 M) to 6.4, and the reaction was incubated at room temperature for 45 min. Then, solid TCEP.HCl (10 mg) was dissolved in the reaction to reduce any possible scrambled disulfides. Next, the reaction was desalted with H_2_0_(aq)_ (0.1% TFA) using size exclusion (5 kDa, PD-10 column). The different fractions (1 mL) were analyzed by analytical HPLC, and those containing the product were pooled and freeze-dried, yielding **F1–F2-CO­(MESNa+thiolactone)** (26.0 mg, 100%) that was used for the next step without further purification. Mass (MALDI-TOF): calculated F1–F2-CO­(MESNa) [M + H]^+^ (average isotopes) 4663.7, found 4663.8. Calculated F1–F2-CO­(thiolactone) [M + H]^+^ (average isotopes) 4521.6, found 4521.6.

### NCL between F1–F2-CO­(MESNa+Thiolactone) and F3

**F3** (12.2 mg, 4.9 × 10^–3^ mmol) was dissolved in Gdm.HCl (6.0 M)–NaPhos buffer (0.2 M, 1.5 mL, pH = 6.6) containing: DSeESNa (50 mM), TCEP.HCl (100 mM) and ascorbic acid (100 mM). The mixture was added to the crude **F1–F2-CO­(MESNa+thiolactone)** peptide (20.0 mg, 4.3 × 10^–3^ mmol), and incubated at room temperature for 2 h (final ligation pH = 6.5). The reaction was directly purified by semipreparative HPLC, yielding the linear **CTX5** sequence (23.4 mg, 77%). Mass (ESI-qTOF): calculated F1–F2–F3 [M]^+^ (average isotopes) 7014.4, found 7014.2.

### Folding of Linear CTX5

The purified linear **CTX5** (5.0 mg, 7.1 × 10^–4^ mmol) was dissolved in degassed Tris buffer (0.1 M, 5 mL, pH = 8.0) containing NaCl (0.15 M). To this mixture, a degassed buffered solution (Tris 0.1 M, NaCl 0.15 M, 5 mL, pH = 8.0) of l-Glutathione reduced (4.0 mM) and l-Glutathione oxidized (1.0 mM) was added to the peptide. The resulting mixture was rotary stirred for 24 h at room temperature under N_2(g)_. The folding was monitored by analytical HPLC and mass spectrometry. The crude reaction was directly purified by HPLC, yielding the oxidized **CTX5** (1.1 mg, 22%). Mass (ESI-qTOF): calculated CTX5 [M]^+^ (average isotopes) 7006.4, found 7006.6.

### Recombinant Expression of His_6_–SUMO2­(ΔG)–GyrA–CBD

*E. coli* BL21­(DE3) cells were transformed with the plasmid pSS01.^50^ The cells were grown in LB medium supplemented with ampicillin (100 μg/mL) at 37 °C and 180 rpm. A preinoculum of 3 mL was grown overnight and then transferred to 0.6 L of medium. When the OD_600_ reached values between 0.6 and 0.8, IPTG (0.1 mM final concentration) was added to induce the expression. The expression was carried out at 20 °C with shaking at 180 rpm. After 18 h, the cells were harvested by centrifugation (16800 rcf, 4 °C, 30 min). The pellet was resuspended in Ni-NTA buffer (NaPhos 50 mM, NaCl 0.3 M, pH = 8.0, 5 mL) and lysed on ice using ultrasonication (60% amplitude, on/off pulses of 15 s, 15 min total time). The lysate was cleared up by centrifugation (6500 rcf, 4 °C, 15 min), and the supernatant was purified by IMAC (1 mL column bed volume). The protein stocks were made by adding 100 μL of glycerol to 1 mL of the protein eluate. The protein concentration was quantified by UV at 280 nm (ε = 36900 M^–1^ cm^–1^).

### EPL between His_6_–SUMO2­(ΔG)–GyrA–CBD and CTAFS

A solution containing the ligation buffer (2x, 1 mL) and **CTAFS** was prepared in a polypropylene tube: DSeESNa (56.0 mg, 0.1 mmol), TCEP·HCl (57.4 mg, 0.2 mmol), ascorbic acid (35.2 mg, 0.2 mmol), and the peptide **2** (1.05 mg, 1.6 × 10^–3^ mmol) were weighed and dissolved in Ni-NTA buffer (0.9 mL). The pH was adjusted to 8.0 with NaOH_(aq)_ (10 M) and Ni-NTA buffer. The ligation solution (0.300 mL) was diluted with the Ni-NTA buffer (0.254 mL, pH = 8.0), and the resulting mixture was incubated with **His**_**6**_ **–SUMO2­(ΔG)–GyrA–CBD** (0.046 mL, 6.0 × 10^–3^ μmol, 130 μM stock). The reaction was flushed with N_2(g)_ and conducted overnight. Aliquots of the ligation were withdrawn at specific times and stored at −20 °C for SDS-PAGE analysis. The ligation pH, checked at the beginning and at the end of the reaction, was 7.8. Mass (ESI-qTOF): calculated [M + H]^+^ (main isotopes) for C_516_H_810_N_159_O_165_S_5_: 12033.8483, observed deconvoluted mass: 12033.8367.

### Recombinant Expression of His_6_–SUMO–Shh^1–159^–GyrA

For expression, *E. coli* Rossetta (DE3) cells were transformed with the corresponding plasmid (Supporting Information page S57). Then, a preinoculum of 3 mL was grown overnight and then transferred to 1 L of medium. Cells were grown in LB medium supplemented with kanamycin (50 μg/mL) and chloramphenicol (34 μg/mL) for 2 h at 37 °C, shaking at 180 rpm. When the OD_600_ reached values between 0.6 and 0.8, IPTG (0.1 mM final concentration) was added to induce the expression. After 2 h, the cells were harvested by centrifugation (30000 rcf, 4 °C, 30 min). The pellet was resuspended in the Ni-NTA buffer (pH = 7.0, 5 mL) and lysed in an ice bath by ultrasonication (60% amplitude, on/off pulses of 15 s, 15 min total time). The lysate was cleared up by centrifugation (3800 rcf, 4 °C, 15 min), and the supernatant was purified by IMAC (Cytiva HisTrapTM HP, 5 mL column bed volume). The eluate was concentrated to 2.5 mL (Amicon Ultra - Centrifugal Filters -3K, 6500 rcf, 4 °C). Finally, the buffer was exchanged through PD-10 columns to STE (Tris 10 mM, NaCl 100 mM, EDTA 1.0 mM, pH = 6.5)/glycerol (10%). The expression yield (29.4 mg/L) was calculated by UV quantification at 280 nm (ε = 42860 M^–1^ cm^–1^).

SDS-PAGE analysis of the stock protein also revealed the presence of the hydrolyzed His_6_-SUMO-Shh^1–159^-COOH in the mixture, retained from the IMAC purification (Figures S78, S79, and S80).

### EPL between His_6_–SUMO–Shh^1–159^–GyrA and Cys^160^–Gly^174^–K­(PEG)–Biotin

**Cys** ^**160**^ **–Gly** ^**174**^ **–K­(PEG)–Biotin** (2.05 mg, 1.0 × 10^–3^ mmol) was dissolved in STE buffer (0.266 mL). This solution was diluted in the ligation STE buffer (2x, 0.350 mL) containing: DSeESNa (50 mM), TCEP.HCl (100 mM) and ascorbic acid (200 mM). Finally, **His**_**6**_ **–SUMO–Shh** ^**1–159**^ **–GyrA** (0.133 mL of a 262 μM stock in STE buffer, 3.5 × 10^–2^ μmol) was added, and the resulting mixture was incubated at 23 °C with occasional stirring. The pH, checked at the beginning and at the end of the reaction, was 6.5. After 4 h, a small amount of TCEP.HCl (∼ 5 mg) was added to the mixture. Next, the buffer was exchanged through size exclusion to Ni-NTA (NaPhos 50 mM, NaCl 300 mM, pH = 8.0), and the reaction product was purified by IMAC (Cytiva HisTrapTM HP 1 mL column bed volume). Following the elution, the imidazole was removed by buffer exchange (PD-10) with STE/glycerol (10%), and the sample was concentrated (Amicon Ultra - Centrifugal Filters -3K, 6500 rcf, 4 °C). UV quantification at 280 nm (ε = 28420 M^–1^ cm^–1^) of the resulting stock (1.0 mL, 12.5 μM) resulted in a 34% yield (combined with the remaining His_6_–SUMO–Shh^1–159^–COOH). Mass (ESI-qTOF): calculated [M]^+^ (average isotopes) for C_1413_H_2225_N_419_O_439_S_10_: 32427.4370, observed deconvoluted mass: 32427.1875.

## Supplementary Material



## Notes

Detailed experimental procedures for the ligation reactions; MALDI-TOF mass spectrometry; LC-MS analysis; SDS-PAGE of the protein ligations; NMR of the model peptides and folded CTX5; HPLC traces of the reactions; curve fitting of the experimental data and calculation of the rate constants (PDF)

## #

BCN Peptides SA, 08777 Sant Quintí de Mediona, Spain
