# Supplementary material for: Catalysis of Native Chemical Ligation and Expressed Protein Ligation by Alkylselenols
Source: JACS Au. 2025 Dec 2;5(12):5948–64. doi: 10.1021/jacsau.5c00793 (PMC12728643; doi:10.1021/jacsau.5c00793)
Supplement: Supplementary file 1 [file au5c00793_si_001.pdf]

Supporting Information for Publication

Catalysis of Native Chemical Ligation and Expressed Protein Ligation by Alkylselenols.

Iván Sánchez-Campillo,[1,6] Esther Gratacòs-Batlle,[2] Selene Pérez-García,[3] Hong S. Nguyen,[4] Gemma Triola,[3] Henning D. Mootz,[5] Juan B. Blanco-Canosa.[3,*]

[1]Institute for Advanced Chemistry of Catalonia (IQAC), Spanish National Research Council (CSIC), 08034 Barcelona, Spain; Department of Inorganic and Organic Chemistry, Section of Organic Chemistry, University of Barcelona, 08028 Barcelona, Spain.

[2]Fundamental and Clinical Nursing Department, Faculty of Nursing, Institute of Neurosciences, University of Barcelona, 08907 L´Hospitalet de Llobregat, Spain.

[3]Institute for Advanced Chemistry of Catalonia (IQAC), Spanish National Research Council (CSIC), 08034 Barcelona, Spain.

[4]Laboratoire de Chimie et Biochimie Pharmacologiques et Toxicologiques, UMR 8601 CNRS, Université Paris Cité, 75006 Paris, France.

[5]Institute of Biochemistry, University of Münster, 48419 Münster, Germany.

[6]Current address: BCN Peptides SA, 08777 Sant Quintí de Mediona, Spain.

*Email: juanbautista.blanco@iqac.csic.es

Contents

1. Chemicals S-2
2. Buffers S-3
3. Instrumentation and methods S-4
4. Characterization of DSeESNa S-6
5. Model peptide synthesis S-9
   - 5.1. LYRAV-CO(SeESNa) (**1**) S-9
   - 5.2. LYRAV-CO(4-MPAA) S-10
   - 5.3. LYRAV-CO(MESNa) (**5**) S-11
   - 5.4. LYRAV-CO(Nbz)-G (**6**) S-12
   - 5.5. LYRAA-CO(SeESNa) S-13
   - 5.6. LYRAP-CO(SeESNa) S-14
   - 5.7. LYRAS-CO(SeESNa) S-15
   - 5.8. LYRAsCTAFS S-16
   - 5.9. Shh Cys^{160}-Gly^{174}-K(PEG)-Biotin S-17
   - 5.10. Shh Cys^{160}-Gly^{174}-PEG-His_6 S-18
6. Model peptide ligations S-19
   - 6.1. MS of the ligation products S-19
   - 6.2. LYRAV-CO(SeESNa) (**1**), determination of k_1 S-20
   - 6.3. LYRAV-CO(SeESNa) (**1**), determination of k_2 and k_{-2} S-22
   - 6.4. LYRAV-CO(SeESNa) (**1**), determination of k_{int} S-24
   - 6.5. LYRAV-CO(4-MPAA) S-26
   - 6.6. LYRAV-CO(SeESNa) (**1**), catalyzed by PhSeH S-28
   - 6.7. LYRAV-CO(MESNa) (**5**), catalyzed by SeESNa S-30
   - 6.8. LYRAV-CO(SeESNa) (**1**) equilibrium with MESNa, determination of $k_{Se\rightarrow S}$ and $k_{S\rightarrow Se}$ S-32
   - 6.9. LYRAVC(LYRAV[COS-])TAFS (**4**), catalyzed by PhSeH S-34
   - 6.10. LYRAV-CO(MESNa) (**5**), catalyzed by PhSeH S-35
   - 6.11. LYRAV-CO(Nbz)-G (**6**), catalyzed by SeESNa S-36
   - 6.12. LYRAA-CO(SeESNa) S-39
   - 6.13. LYRAP-CO(SeESNa) S-41
   - 6.14. LYRAP-CO(SeESNa), catalyzed by PhSeH S-43
   - 6.15. LYRAS-CO(SeESNa) S-45
7. Chemical synthesis of Cardiotoxin A5 (CTX5) S-48
   - 7.1. F1-CO(Nbz)-$CONH_2$ S-48
   - 7.2. F2-CO(Dbz) S-48
   - 7.3. F3 S-49
   - 7.4. F1-F2-CO(Dbz) S-49
   - 7.5. F1-F2-CO(MESNa) S-50
   - 7.6 F1-F2-CO(MESNa) + SeESNa, stability test S-51
   - 7.7. F1-F2-F3 S-52
   - 7.8. Oxidation and folding: CTX5 S-53
   - 7.9. NMR of CTX5 S-54
8. Protein expression and SeESNa-catalyzed Expressed Protein Ligation S-55
   - 8.1. Expression of His_6-SUMO2(ΔG)-GyrA-CBD S-55
   - 8.2. Synthesis of His_6-SUMO2(ΔG)-CTAFS by SeESNa-catalyzed EPL S-55
   - 8.3. Cloning and expression of His_6-SUMO-Shh^{1-159}-GyrA S-57
   - 8.4. Synthesis of His_6-SUMO-Shh^{1-174}-PEG-His_6 and His_6-SUMO-Shh^{1-174}-K(PEG)-Biotin by SeESNa-catalyzed EPL S-61
   - 8.5. Analysis of the EPL between His_6-SUMO-Shh^{1-159}-GyrA and Cys^{160}-Gly^{174}-K(PEG)-Biotin catalyzed by MESNa, 4-MPAA, and SeESNa S-66
9. References S-67

1. Chemicals

Supplementary table 1. Reagents and suppliers.

Reagent	Vendor
Guanidine hydrochloride (Gdm.HCl), kanamycin sulphate, (R,R)-chloramphenicol, streptavidin, Phusion polymerase system, GeneJet kit, Page Ruler Unstained Protein Ladder, Sodium 2-bromoethanesulfonate, HisPur^{TM} Ni-NTA resin	ThermoFisher Scientific
BlueEye Prestained Protein Ladder	Jena Bioscience
Tris(2-carboxyethyl)phosphine hydrochloride (TCEP.HCl)	Biosynth
DCM, Et_2O, piperidine	Carlo Erba
Acetonitrile (ACN, HPLC grade)	Fisher Chemical
Tris(3-hydroxypropyl)phosphine (80%)	Strem
Sodium acetate anhydrous (98.5%)	Fluka
Diphenyl diselenide	TCI
Acetic acid glacial	Panreac
Trifluoroacetic acid (TFA, peptide grade), 2-(1H-7-azabenzotriazol-1-yl)-1,1,3,3-tetramethyluronium hexafluorophosphate (HATU), Sodium 2-mercaptoethanosulphonate (MESNa, 91%), 1-Fluoro-2-nitrobenzene	Fluorochem
Fmoc-8-amino-3,6-dioxaoctanoic acid	PolyPeptide
2-(1H-Benzotriazol-1-yl)-1,1,3,3-tetramethyluronium hexafluorophosphate (HBTU) Fmoc-amino acids, Boc-L-Cys(Trt)-OH, Fmoc-O2Oc-OH, DMF (peptide synthesis grade) Resins: Aminomethyl-ChemMatrix (0.56 mmol/g), Fmoc-Rink Amide AM (0.74 mmol/g)	Iris Biotech
Fmoc-Dbz-CONH-Rink-polystyrene resin (0.49 mmol/g), NovaSyn TGT resin (0.1 mmol/g)	Novabiochem
HMPB-ChemMatrix resin (0.6 mmol/g)	Matrix Innovation
Boc-L-Leu-OH	Neosystem
N,N-diisopropylethylamine (DIEA), disodium hydrogen phosphate (NaPhos), triisopropylsilane (TIS), ethylenediaminetetracetic acid (EDTA), selenium, sodium borohydride ($NaBH_4$), ascorbic acid, 4-mercaptophenylacetic acid (4-MPAA), reduced Glutathione (GSH), oxidized Glutathione (GSSG), L-Tyr (>98%), *tert*-Butyl nitrite (^{t}BuONO), glycerol, bovine serum albumin (BSA), phosphate buffered saline (PBS), Tween-20, NaOH, $HCl_{(aq)}$ (37%), NaCl (>99%), EtOH (analytical grade), *p*-nitrophenyl chloroformate, sodium dodecyl sulphate (SDS), *N,N,N',N'*-tetramethylethylendiamine (TMED), ammonium persulphate, Tris(hydroxymethyl)aminomethane (Tris), ampicillin trihydrate, α-cyano-4-hydroxycinnamic acid	Sigma-Aldrich
30% Acrylamide/Bis solution (37.5:1), Laemmli sample buffer 4x, Tris/Glycine/SDS 10x, Coomassie Brilliant Blue R-250 protein stain powder	BioRad
Antimouse-HRP antibody	Invitrogen
KLD enzyme Mix (ref. number: M0554S)	New England Biolabs
3,3′,5,5′-Tetramethylbenzidine (TMB)	BioLegend
Yeast extract	Oxoid
Peptone	Pronadisa
D_2O, DMSO-d_6	Eurisotop

Fmoc-PAL-OH (Fmoc-4-(aminomethyl)-3,5-dimethoxyphenoxy) butanoic acid) was prepared following reported protocols.[1,2]

All experiments were carried out using H_2O purified with a Millipore system (resistivity 18.2 MΩ.cm).

2. Buffers

Supplementary table 2. Buffers and their compositions.

Buffer	Composition
HPLC buffer A (analytical)	H_2O (0.045% TFA)
HPLC buffer B (analytical)	ACN (0.036% TFA)
HPLC buffer A (semipreparative)	H_2O (0.1% TFA)
HPLC buffer B (semipreparative)	ACN (0.05% TFA)
Guanidinium.HCl / phosphate buffer	Gdm·HCl (6 M), NaPhos (0.2 M), pH = 7.3
Ni-NTA buffer	NaPhos (50 mM), NaCl (300 mM), pH = 7.0 – 8.0
STE buffer	Tris (10 mM), NaCl (100 mM), EDTA (1.0 mM), pH = 6.5
LB medium	Yeast extract (5 g/L), Peptone (10 g/L), NaCl (10 g/L)
PBS	NaCl (137) mM, KCl (2.7 mM), Na_2HPO_4 (10 mM), KH_2PO_4 (1.8 mM), pH = 7.4
ELISA blocking buffer	BSA (2 µg/mL in PBS)
ELISA PT buffer	Tween-20 (0.5% in PBS)
ELISA PBT buffer	BSA (2 µg/mL) and Tween-20 (0.5%) in PBS

3. Instrumentation and methods

Analytical RP-HPLC.

Chromatographic separations were carried out in a binary buffer system using lineal gradients and the following columns:

- Column A: Phenomenex Aeris PEPTIDE XB-C18 (3.6 µm, 150 mm x 4.60 mm)
- Column B: Waters XBridge C18 (5 µm, 150 mm x 4.60 mm)
- Column C: Waters Symmetry300 C4 (5 µm, 150 mm x 4.60 mm)

- Gradient 1: 0 to 46% of buffer B in buffer A, t = 20 min. Then, 100% ACN, t = 3 min.
- Gradient 2: 0 to 40% of buffer B in buffer A, t = 40 min. Then, 100% ACN, t = 3 min.
- Gradient 3: 0 to 30% of buffer B in buffer A, t = 20 min. Then, 100% ACN, t = 3 min.

MALDI-TOF MS.

The Bruker Peptide Calibration Standard II (ref. 8222570) was used as calibration standard: Angiotensin II (m/z: 1046.5418 $[M+H]^+$), Angiotensin I (m/z: 1296.6848 $[M+H]^+$), Substance P (m/z: 1347.7354 $[M+H]^+$), bombesin (m/z: 1619.8223 $[M+H]^+$), Renin substrate (m/z: 1758.9326 $[M+H]^+$), ATCH clip 1-17 (m/z: 2093.0826 $[M+H]^+$), ATCH clip 18-39 (m/z: 2465.1983 $[M+H]^+$), and Somatostatin (m/z: 3147.4710 $[M+H]^+$).

IR (film).

IR spectrum was registered in a Nicolet Avatar 360 FT-IR spectrometer with EZ OMNIC E.S.P. 5.2, and plotted using GraphPad Prism 8.

PCR.

An Eppendorf 5332 Mastercycler personal thermalcycler was used for PCR procedures. DNA was amplified with the Phusion polymerase system (ThermoFisher F-534S) using a [dTNP] = 0.2 µM. The amplified sequences were purified with GeneJet kits (ThermoFisher K0702) according to manufacturer's instructions. The nucleotide sequences obtained from PCR products were validated by enzyme restriction and DNA sequencing.

His-tag protein purification.

His tagged proteins were purified in Cytiva HisTrap™ HP columns. In a typical purification, the column was first equilibrated with the Ni-NTA buffer. Next, the sample was loaded with 2 column volumes (CV) of Ni-NTA buffer, and then washed with 2 CV of imidazole (20 mM in Ni-NTA buffer), followed by other 2 CV of imidazole (40 mM in Ni-NTA buffer). After washing, the protein was eluted with 2 CV of imidazole (250 mM in Ni-NTA buffer).

Buffer exchange.

Protein buffer exchange was performed in Cytiva PD-10 desalting columns (size exclusion ~ 5000 Da). According to manufacturer's instructions, a typical procedure for buffer exchange was run as follows: the column was first equilibrated with the desired exchange buffer (25 mL). Then, the protein sample was loaded in the column in a fixed volume (2.5 mL). Next, it was eluted with the exchange buffer solution (3.5 mL).

SDS-PAGE.

Gels were cast with polyacrylamide (15 - 18% for separating and 4 % for stacking) and SDS (0.1 % w/v), and run at 230 V for 40 min. BlueEye Prestained Protein Ladder (10 - 245 kDa, Jena Bioscience PS-104) and PageRuler Unstained Protein Ladder (10 - 200 kDa, ThermoFisher 26614) were run as protein molecular weight markers. Gels were stained with Coomassie Brilliant Blue R-250 (0.1% w/v) in $MeOH/AcOH/H_2O$ solution (40:10:50). **Sample loading**: 10 µL of a given sample, TCEP (5 µL, 0.1 mg/mL, pH = 7.0) and Laemmli sample buffer 4x (5 µL) were mixed in a polypropylene tube. 15 µL of this mixture were loaded in the gel without any further treatment.

Potentiometric titrator.

pK_a of SeESNa was determined by potentiometric titration in a Metrohm 904 Titrando.

Protein UV quantification.

Protein concentration was determined by measuring the UV absorption at 280 nm in a ThermoFisher Scientific NanoDrop 8000. Molar extinction coefficients (ε) were obtained using the ProtParam tool from Expasy (https://web.expasy.org/protparam/).

4. Characterization of DSeESNa

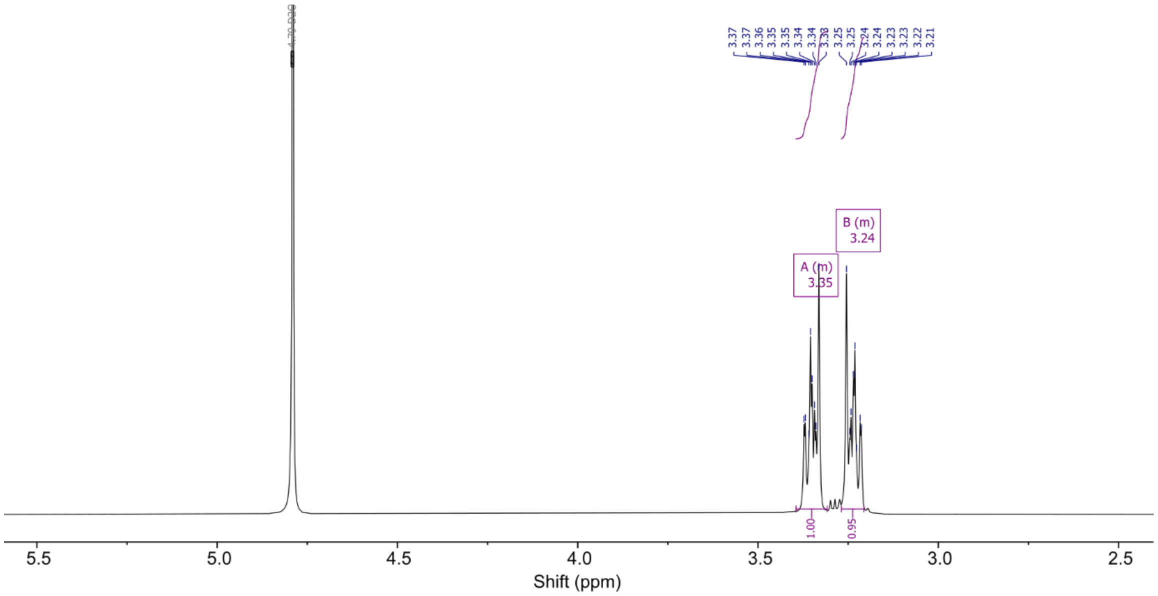

Supplementary figure 1. ^{1}H NMR spectrum of **DSeESNa** (D_2O). δ (ppm): 3.35 (m, 4H), 3.24 (m, 4H).

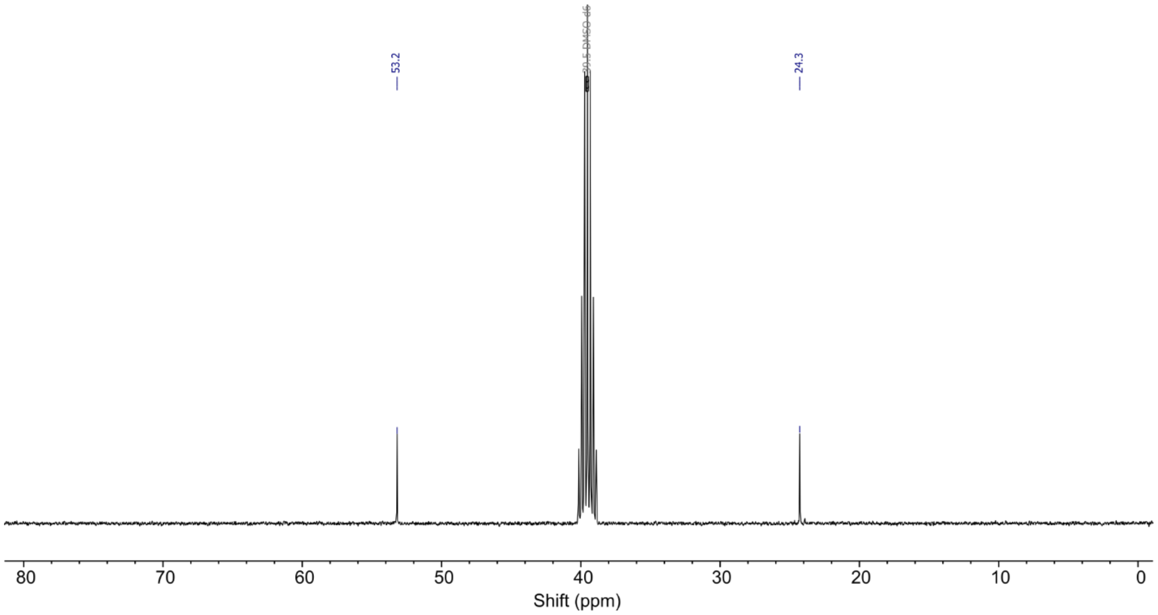

Supplementary figure 2. ^{13}C NMR spectrum of **DSeESNa** (DMSO-d_6). δ (ppm): 53.2, 24.3.

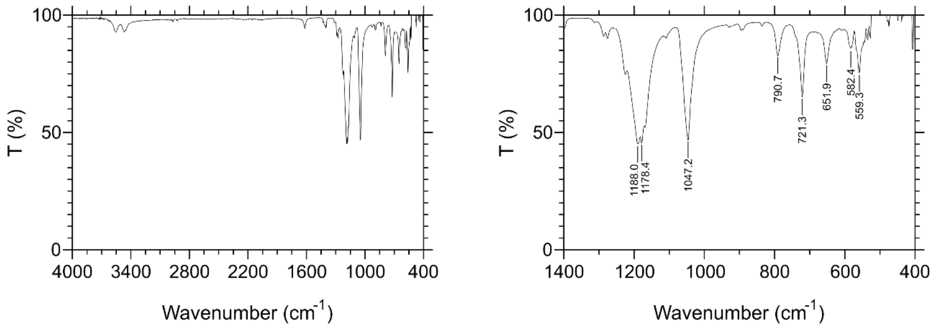

Supplementary figure 3. IR spectrum of **DSeESNa**. ν (cm^{-1}): 1188.0 (s, ν_{as} SO_3), 1178.4 (s, ν_{as} SO_3), 1047.2 (s, ν_s SO_3), 790.7 (w, ν C-SO_3), 721.3 (m, ν Se-Se), 651.9 (w), 582.4 (w), 559.3 (w, ν C-Se).

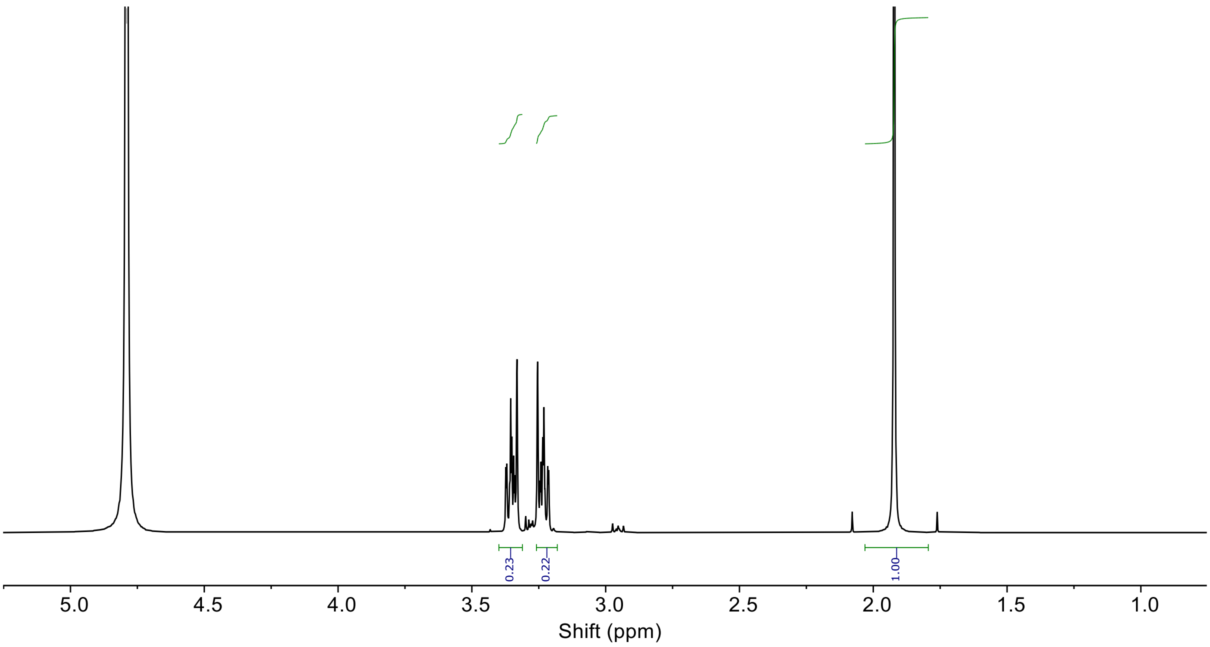

Supplementary figure 4. Quantitative ^{1}H NMR spectrum of **DSeESNa** (D_2O). DSeESNa ($C_4H_8S_2Se_2O_6Na_2$) content: 75%

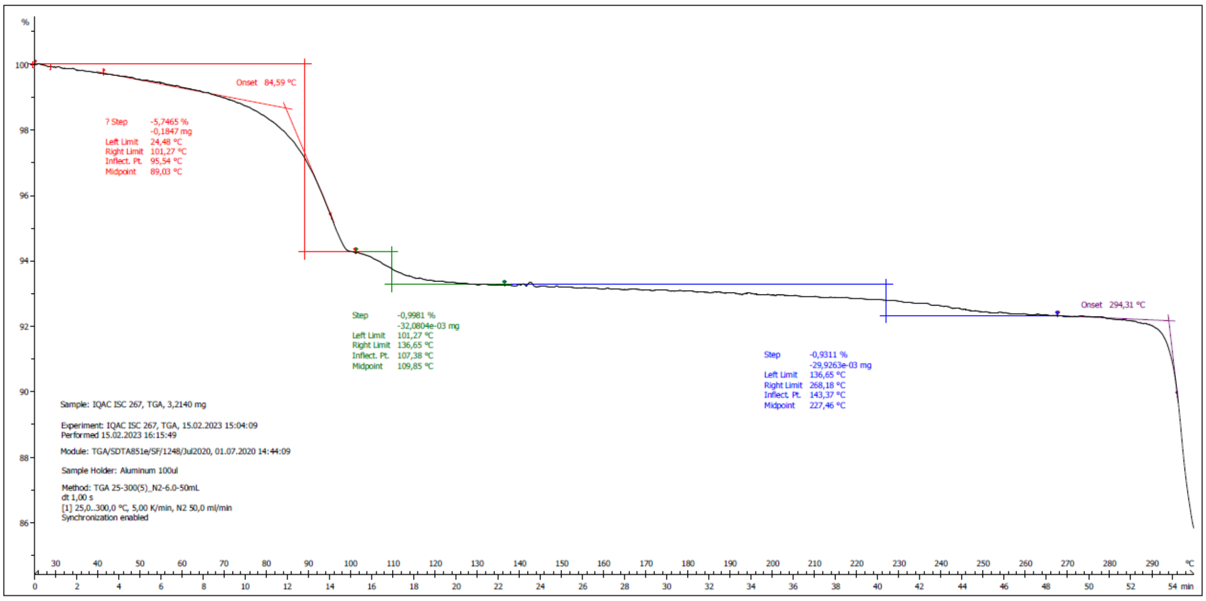

Supplementary figure 5. Thermogravimetric analysis of **DSeESNa**. H_2O content: 7%.

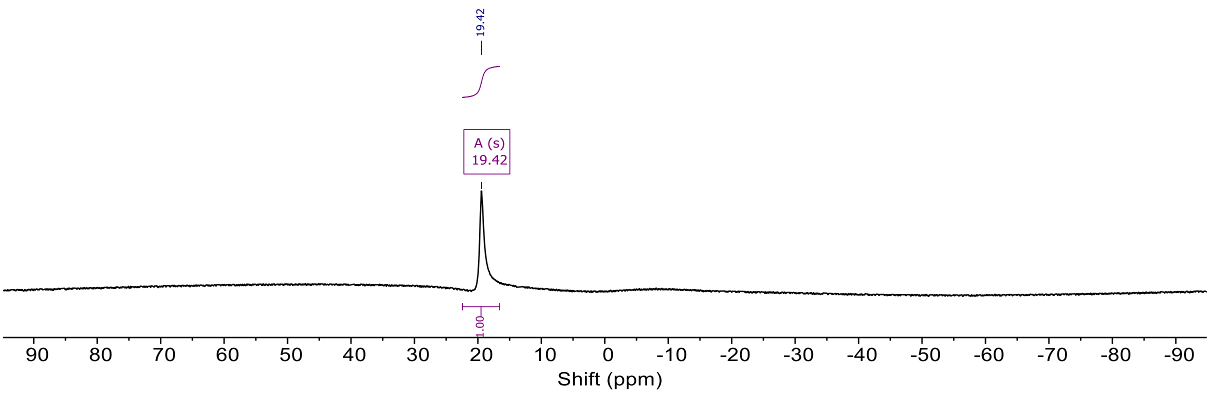

Supplementary figure 6. ^{11}B NMR spectrum of **DSeESNa** (D_2O). δ (ppm): 19.4 (H_3BO_3, expected: 19.6).[3]

pK_a determination.

A total of six replicates were made, and the result is expressed as the average ± standard deviation of the experiments (x ± σ). The pK_a value was determined graphically as the half point in the titration, where [RSeH] = [RSe$^-$], and thus pH = pK_a.[4]

In a typical experiment, a solution of **DSeESNa** in H_2O (3.5 x 10^{-5} mol, 7 mL, 5 x 10^{-3} M) containing NaCl (0.1 M) was flushed with N_2, and acidified with $HCl_{(aq)}$ (6 M) until pH = 2 - 3. Tris(3-hydroxypropyl)phosphine (0.350 mL, 3.5 x 10^{-5} mol, 0.1 M) was added, and the resulting solution was titrated with $NaOH_{(aq)}$ (0.01 M). Two equivalence points are observed: the first corresponds to HCl, and the second to SeESH. The half point between these two equivalence points represents the pK_a value.

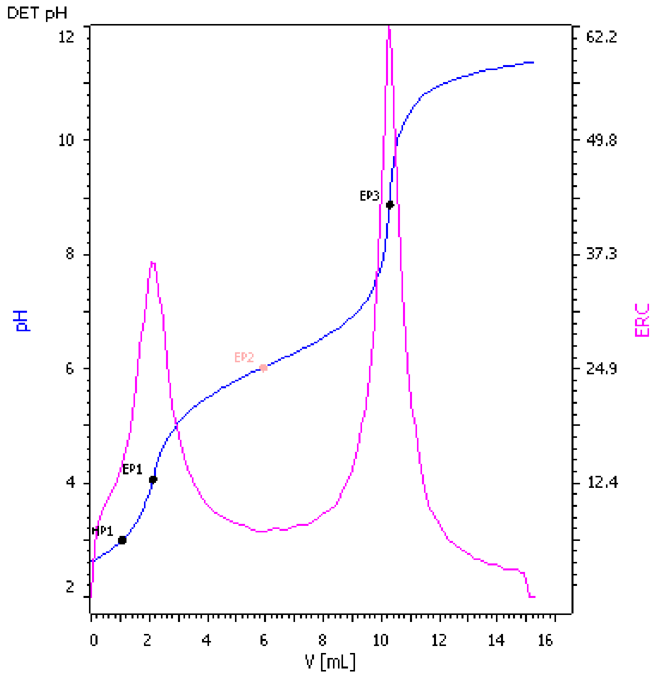

Supplementary figure 7. Titration of **SeESNa** (exp 1). ERC (Equivalence point Recognition Criterion, pink) is the first derivative of the pH curve (blue). In this experiment, pK_a = 6.02.

Supplementary table 3. Obtained values for the determination of the pKa of **SeESNa**.

Exp	pK_a
**1**	6.02
**2**	6.05
**3**	6.09
**4**	6.04
**5**	6.10
**6**	5.99
**Mean**	**6.05 ± 0.04**

5. Model peptide synthesis

5.1. LYRAV-CO(SeESNa) (1)

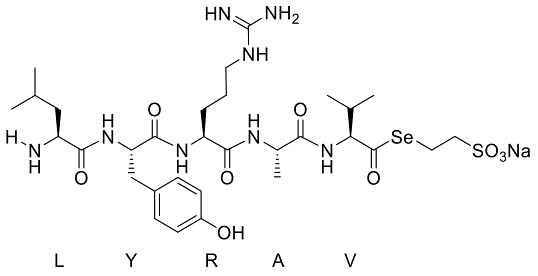

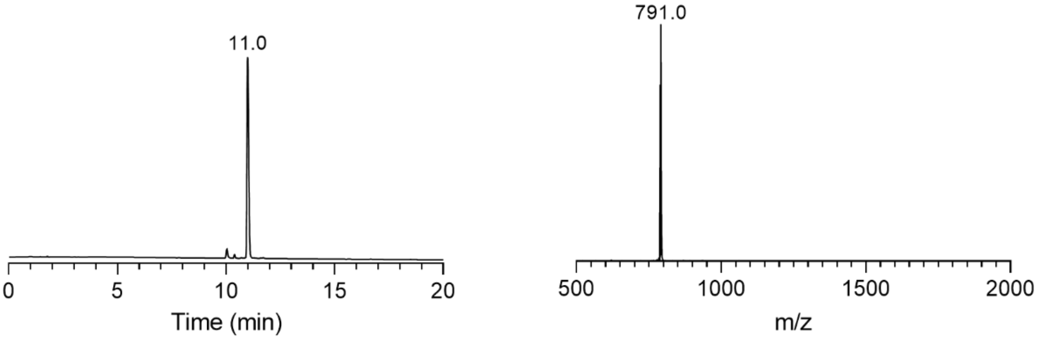

Supplementary figure 8. HPLC trace (220 nm) and MALDI-TOF MS of **LYRAV-CO(SeESNa)**. Column A, gradient 1. Rt = 11.0 min, m/z (**LYRAV-CO(SeESH)**): 791.0 $(M-H)^-$, expected: 790.8.

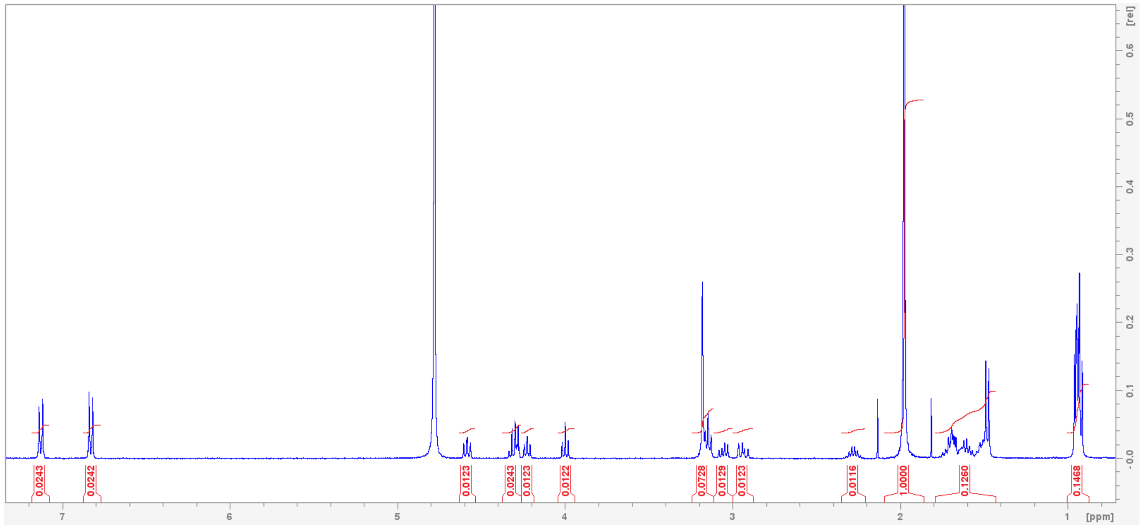

Supplementary figure 9. Quantitative ^{1}H NMR spectrum of **LYRAV-CO(SeESNa)** (D_2O). δ (ppm): 7.13 (d, J = 8.6 Hz, 2H), 6.83 (d, J = 8.6 Hz, 2H), 4.59 (dd, J = 9.0, 7.3 Hz, 1H), 4.35 – 4.26 (m, 2H), 4.23 (t, J = 7.0 Hz, 1H), 4.00 (t, J = 7.3 Hz, 1H), 3.22 – 3.11 (m, 6H), 3.06 (dd, J = 13.6, 7.3 Hz, 1H), 2.94 (dd, J = 13.6, 9.2 Hz, 1H), 2.28 (h, J = 6.7 Hz, 1H), 1.80 – 1.44 (m, 10H), 0.98 – 0.90 (m, 12H). Peptide content (LYRAV-CO(SeESNa)): 78%.

5.2. LYRAV-CO(4-MPAA)

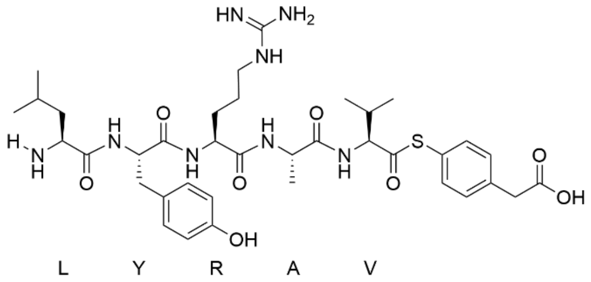

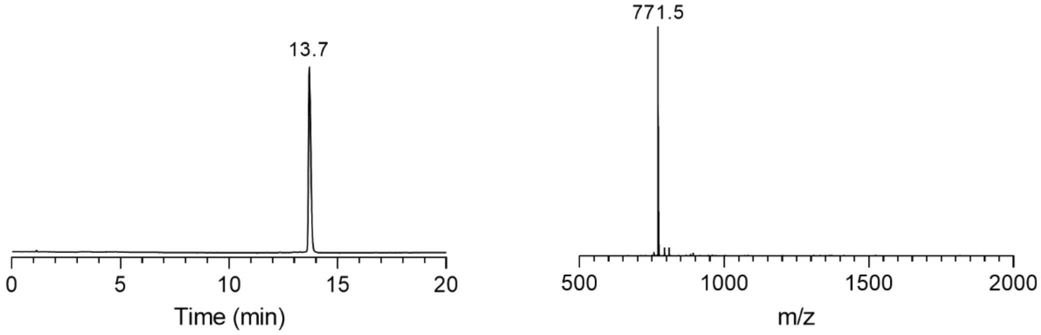

Supplementary figure 10. HPLC trace (220 nm) and MALDI-TOF MS of **LYRAV-CO(4-MPAA)**. Column A, gradient 1. Rt = 13.7 min, m/z: 771.5 $(M+H)^+$, expected: 772.0.

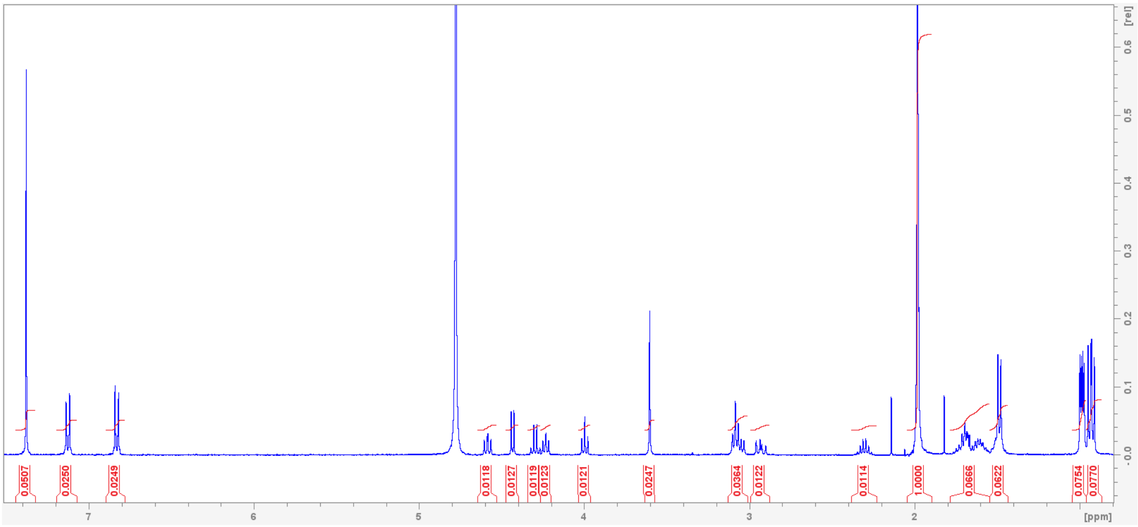

Supplementary figure 11. Quantitative ^{1}H NMR spectrum of **LYRAV-CO(4-MPAA)** (D_2O).δ (ppm): 7.38 (s, 4H), 7.13 (d, J = 8.6 Hz, 2H), 6.83 (d, J = 8.6 Hz, 2H), 4.59 (dd, J = 8.9, 7.3 Hz, 1H), 4.43 (d, J = 6.5 Hz, 1H), 4.29 (q, J = 7.2 Hz, 1H), 4.23 (dd, J = 7.3, 6.5 Hz, 1H), 4.00 (t, J = 7.1 Hz, 1H), 3.60 (s, 2H), 3.12 – 3.02 (m, 3H), 2.94 (dd, J = 13.6, 9.2 Hz, 1H), 2.30 (h, J = 6.7 Hz, 1H), 1.78 – 1.44 (m, 10H), 0.99 (dd, J = 6.8, 3.5 Hz, 6H), 0.93 (dd, J = 8.5, 6.6 Hz, 6H). Peptide content: 67%.

5.3. LYRAV-CO(MESNa) (5)

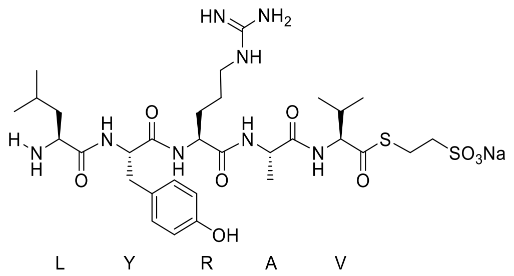

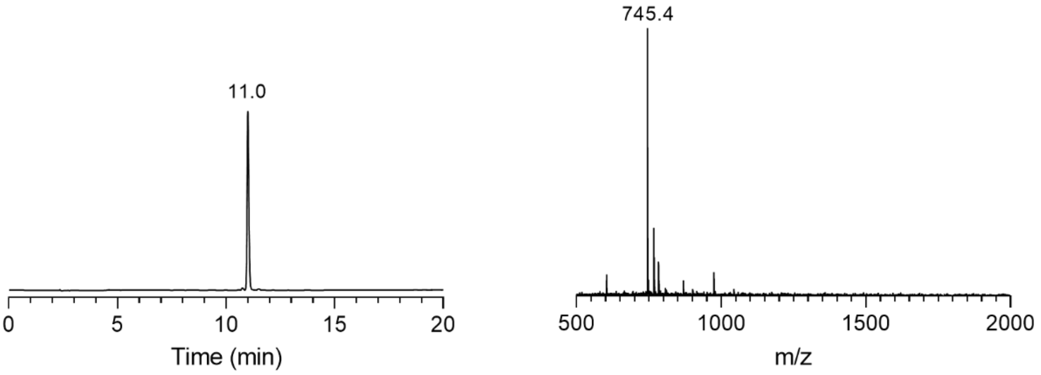

Supplementary figure 12. HPLC trace (220 nm) and MALDI-TOF MS of **LYRAV-CO(MESNa)**. Column B, gradient 1. Rt = 11.0 min, m/z (**LYRAV-CO(MESH)**): 745.4 $(M+H)^+$, expected: 745.9.

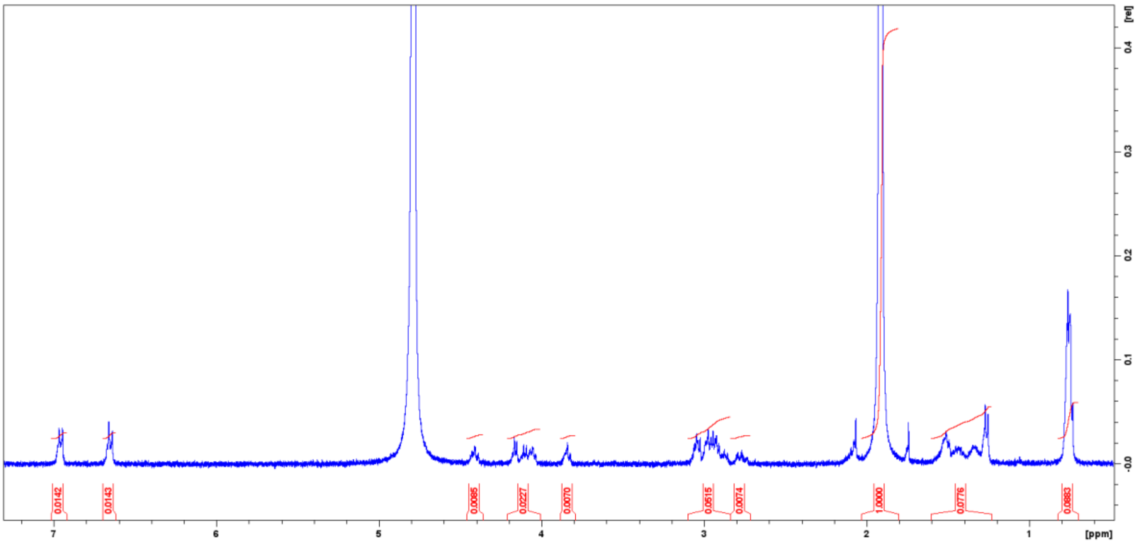

Supplementary figure 13. Quantitative ^1H NMR spectrum of **LYRAV-CO(MESNa)** (D_2O). δ (ppm): 6.96 (d, J = 8.6 Hz, 2H), 6.65 (d, J = 8.6 Hz, 2H), 4.41 (dd, J = 8.9, 7.3 Hz, 1H), 4.21 - 4.02 (m, 3H), 3.84 (m, 1H), 3.01 – 2.84 (m, 7H), 2.83 – 2.72 (m, 1H), 1.60 – 1.23 (m, 10H), 0.83 – 0.70 (m, 12H). Peptide content (LYRAV-CO(MESNa)): 73%.

5.4. LYRAV-CO(Nbz)-G (6)

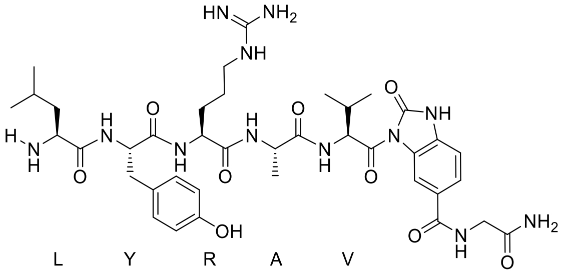

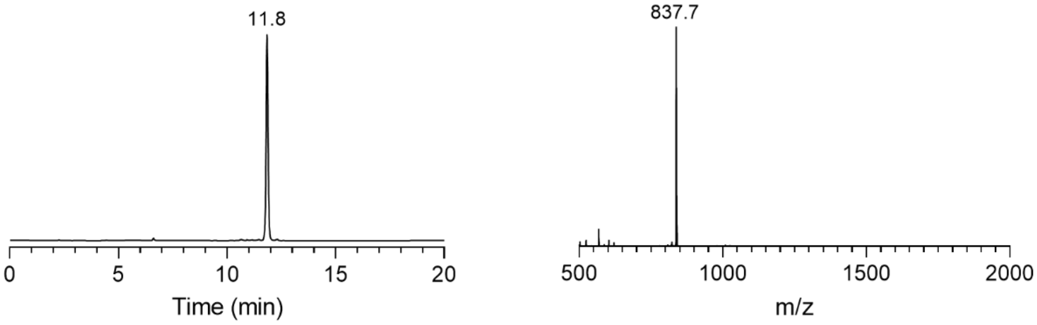

Supplementary figure 14. HPLC trace (220 nm) and MALDI-TOF MS of **LYRAV-CO(Nbz)-G**. Column B, gradient 1. Rt = 11.8 min, m/z: 837.7 $(M+H)^+$, expected: 838.0.

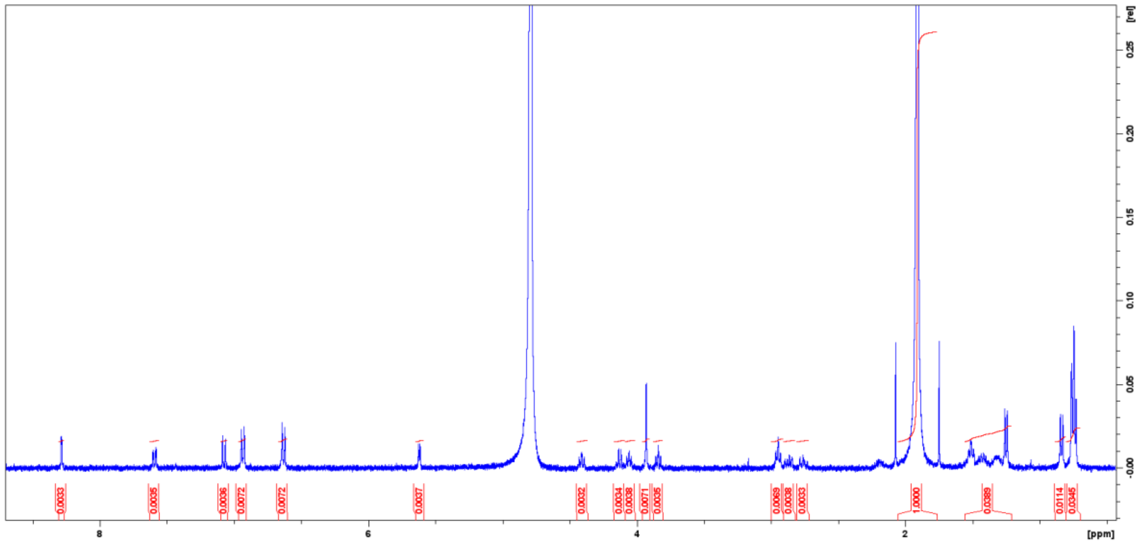

Supplementary figure 15. Quantitative ^{1}H NMR spectrum of **LYRAV-CO(Nbz)-G** (D_2O). δ (ppm): 8.28 (d, J = 1.5 Hz, 1H), 7.59 (dd, J = 8.6, 1.8 Hz, 1H), 7.08 (d, J = 8.6 Hz, 1H), 6.94 (d, J = 8.6 Hz, 2H), 6.63 (d, J = 8.6 Hz, 2H), 5.62 (d, J = 4.7 Hz, 1H), 4.41 (dd, J = 8.9, 7.3 Hz, 1H), 4.13 (q, J = 7.2 Hz, 1H), 4.06 (dd, J = 7.3, 6.5 Hz, 1H), 3.93 (s, 2H), 3.84 (t, J = 7.3 Hz, 1H), 2.95 (t, J = 7.2 Hz, 2H), 2.87 (dd, J = 13.6, 6.7 Hz, 1H), 2.76 (dd, J = 13.6, 8.9 Hz, 1H), 1.56 – 1.20 (m, 11H), 0.83 (d, J = 6.7 Hz, 3H), 0.74 (dd, J = 7.5, 6.5 Hz, 9H). Peptide content: 54%.

5.5. LYRAA-CO(SeESNa)

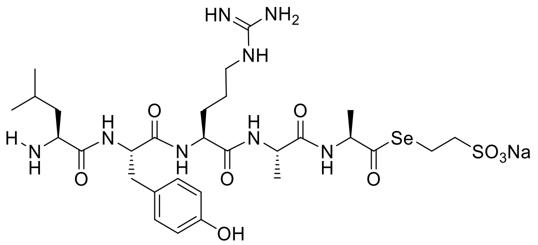

L Y R A A

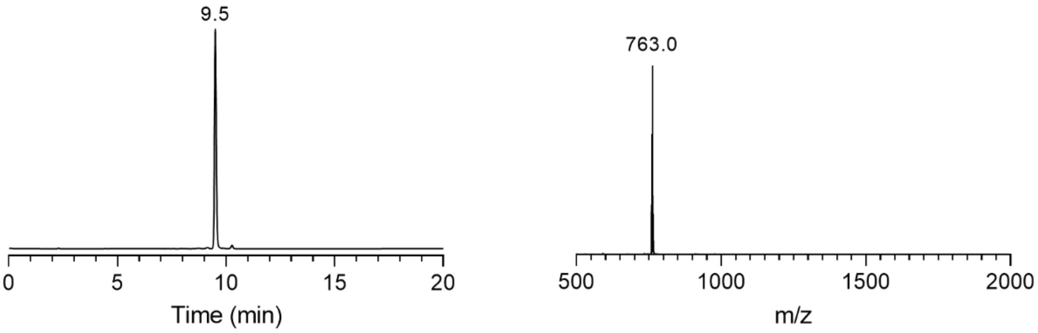

Supplementary figure 16. HPLC trace (220 nm) and MALDI-TOF MS of **LYRAA-CO(SeESNa)**. Column B, gradient 1. Rt = 9.5 min, m/z (**LYRAA-CO(SeESH)**): 763.0 (M-H)$^-$, expected: 762.8.

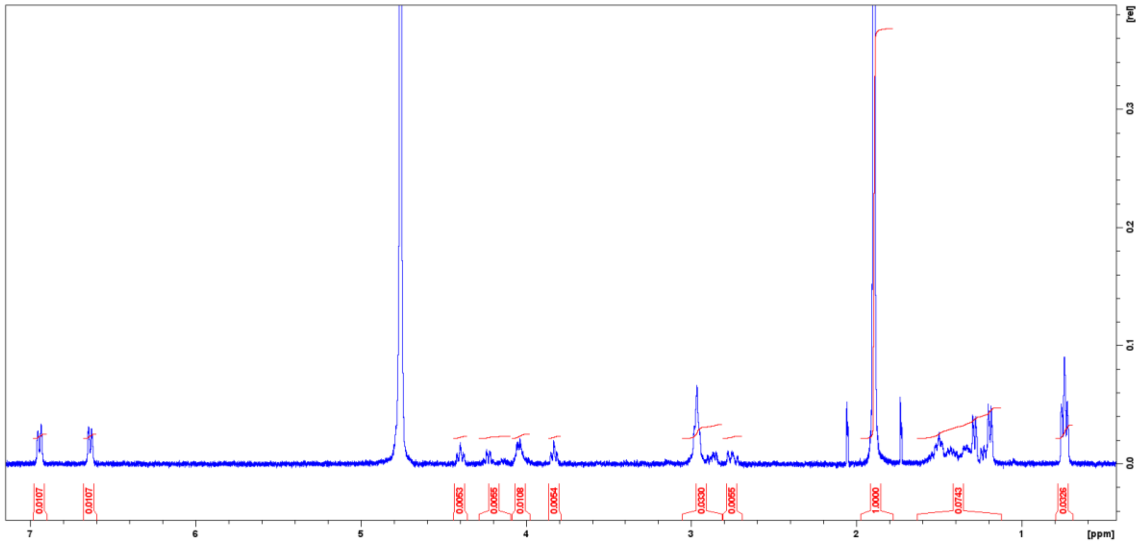

Supplementary figure 17. Quantitative ^{1}H NMR spectrum of **LYRAA-CO(SeESNa)** (D_2O). δ (ppm): 6.94 (d, J = 8.6 Hz, 2H), 6.63 (d, J = 8.6 Hz, 2H), 4.41 (dd, J = 8.9, 7.3 Hz, 1H), 4.28 – 4.10 (m, 1H), 4.09 – 3.98 (m, 2H), 3.82 (t, J = 6.9 Hz, 1H), 3.02 – 2.92 (m, 5H), 2.87 (dd, J = 13.6, 6.7 Hz, 1H), 2.76 (dd, J = 13.6, 8.9 Hz, 1H), 1.63 – 1.13 (m, 14H), 0.77 – 0.71 (m, 6H). Peptide content (LYRAA-CO(SeESNa)): 74%.

5.6. LYRAP-CO(SeESNa)

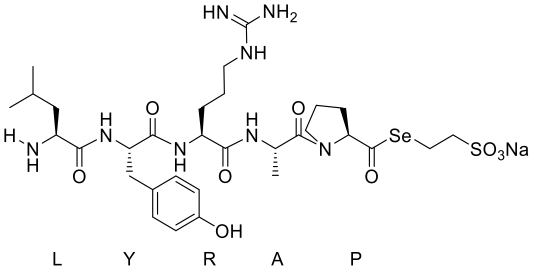

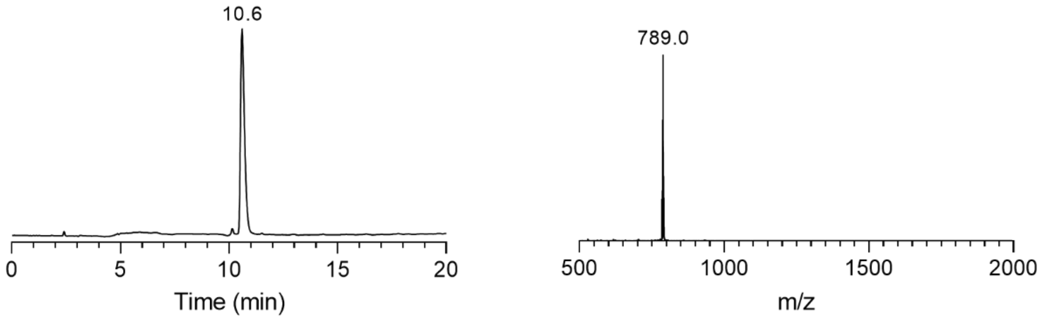

Supplementary figure 18. HPLC trace (220 nm) and MALDI-TOF MS of **LYRAP-CO(SeESNa)**. Column B, gradient 1. Rt = 10.6 min, m/z (**LYRAP-CO(SeESH)**): 789.0 $(M-H)^-$, expected: 788.8.

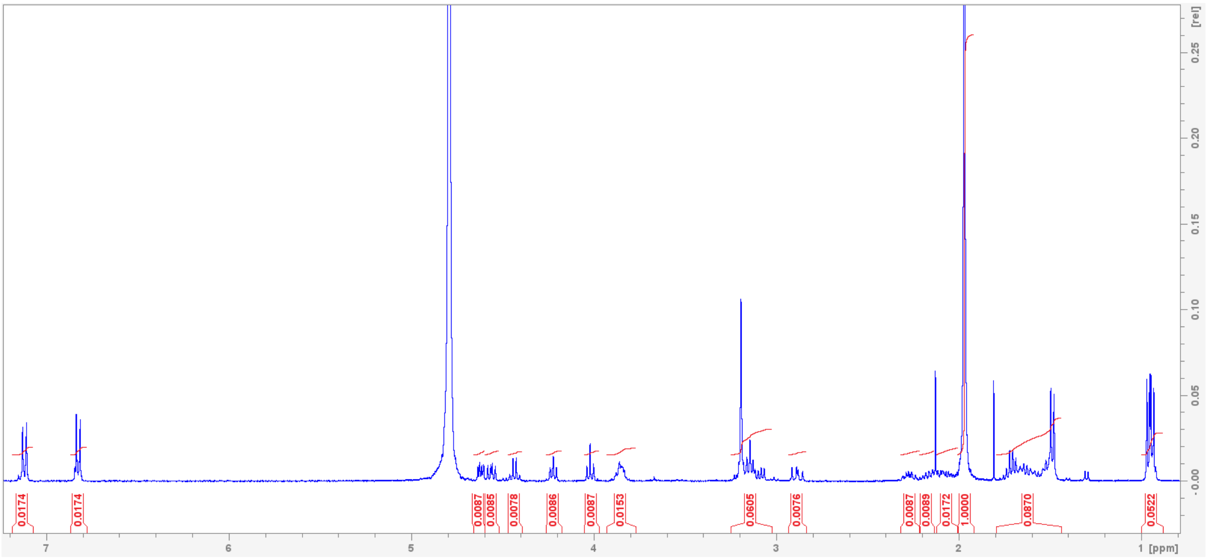

Supplementary figure 19. Quantitative ^1H NMR spectrum of **LYRAP-CO(SeESNa)** (D_2O). δ (ppm): 7.12 (d, J = 8.6 Hz, 2H), 6.82 (d, J = 8.4 Hz, 2H), 4.62 (dd, J = 8.7, 4.1 Hz, 1H), 4.56 (dd, J = 10.0, 6.8 Hz, 1H), 4.43 (q, J = 7.1 Hz, 1H), 4.22 (t, J = 7.0 Hz, 1H), 4.02 (t, J = 7.3 Hz, 1H), 3.90 – 3.80 (m, 2H), 3.24 – 3.04 (m, 7H), 2.88 (dd, J = 13.5, 8.9 Hz, 1H), 2.32 – 2.22 (m, 1H), 2.21 – 2.13 (m, 1H), 2.12 – 2.00 (m, 2H), 1.80 – 1.44 (m, 10H), 0.98 – 0.90 (m, 6H). Peptide content (LYRAP-CO(SeESNa)): 77%.

5.7. LYRAS-CO(SeESNa)

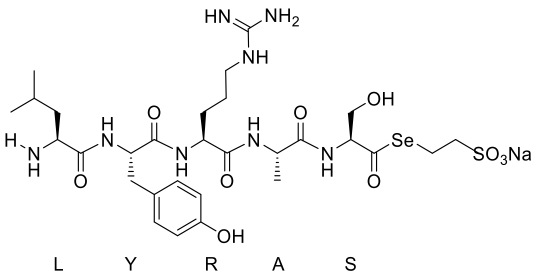

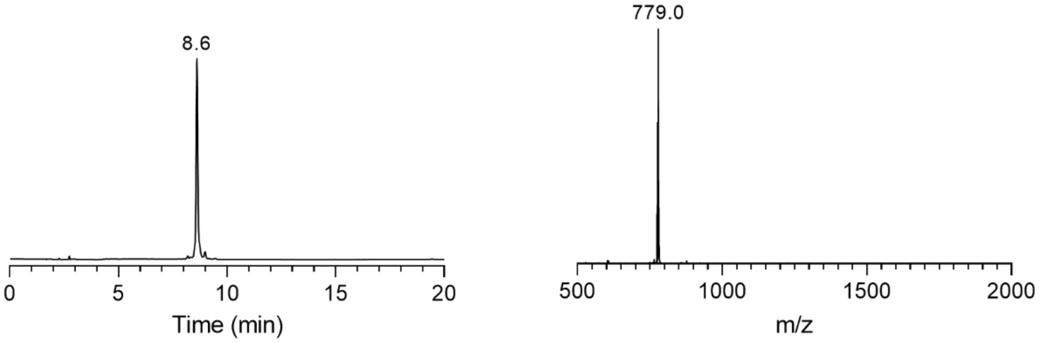

Supplementary figure 20. HPLC trace (220 nm) and MALDI-TOF MS of **LYRAS-CO(SeESNa)**. Column B, gradient 1. Rt = 8.6 min, m/z (**LYRAS-CO(SeESH)**): 779.0 (M-H)$^-$, expected: 778.8.

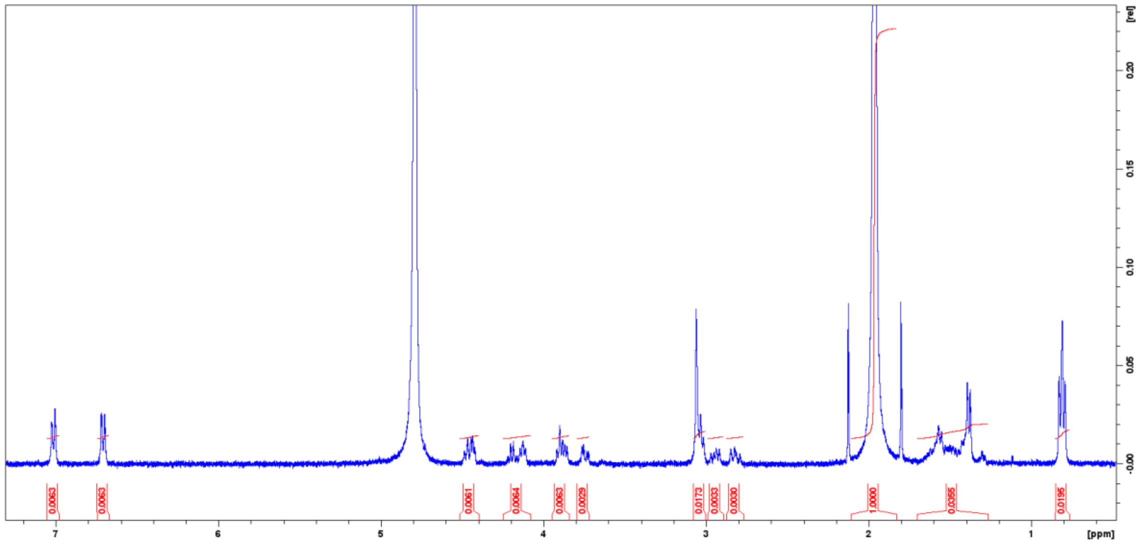

Supplementary figure 21. Quantitative ^{1}H NMR spectrum of **LYRAS-CO(SeESNa)** (D_2O). δ (ppm): 7.01 (d, J = 8.6 Hz, 2H), 6.71 (d, J = 8.6 Hz, 2H), 4.51 – 4.41 (m, 2H), 4.24 – 4.08 (m, 2H), 4.09 – 3.98 (m, 2H), 3.94 – 3.84 (m, 2H), 3.79 – 3.71 (m, 1H), 3.08 – 3.00 (m, 5H), 2.94 (dd, J = 13.6, 6.7 Hz, 1H), 2.82 (dd, J = 13.6, 8.9 Hz, 1H), 1.70 – 1.25 (m, 11H), 0.85 – 0.76 (m, 6H). Peptide content (LYRAS-CO(SeESNa)): 77%.

5.8. LYRAsCTAFS

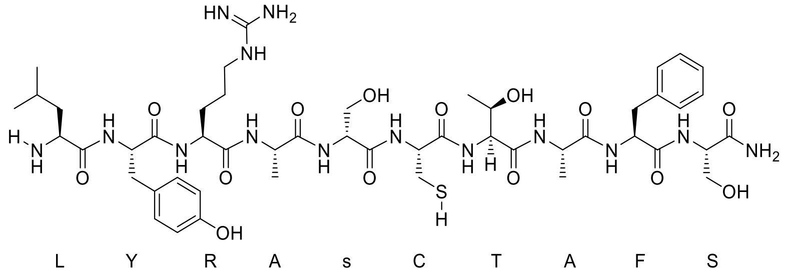

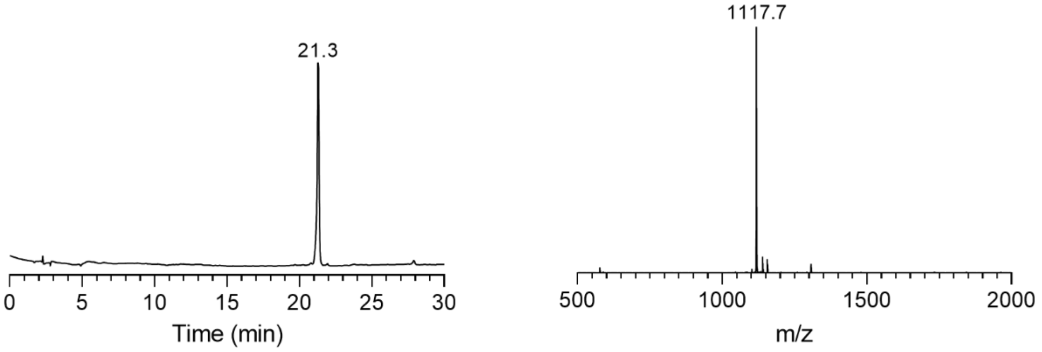

Supplementary figure 22. HPLC trace (220 nm) and MALDI-TOF MS of **LYRAsCTAFS**. Column B, gradient 2. Rt = 21.6 min, m/z: 1117.7 $(M+H)^+$,expected: 1118.3.

5.9. Shh Cys^{160}-Gly^{174}-K(PEG)-Biotin

Synthesis was performed on a Fmoc-Rink-amide resin (0.74 mmol/g, 0.2 mmol scale, Iris Biotech) following the standard Fmoc-SPPS protocols described in the Experimental Section of the manuscript.

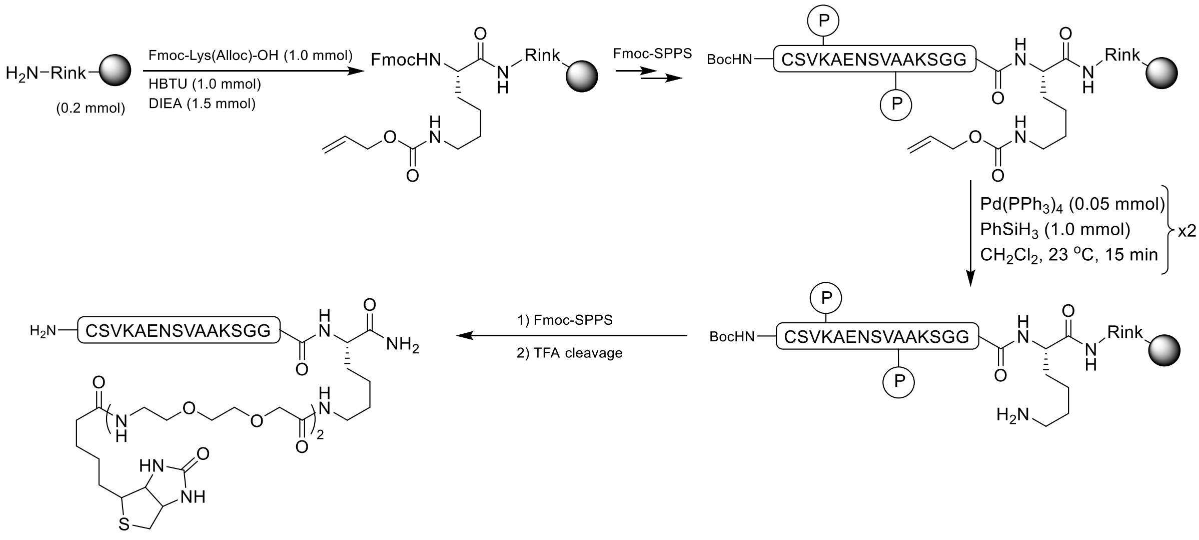

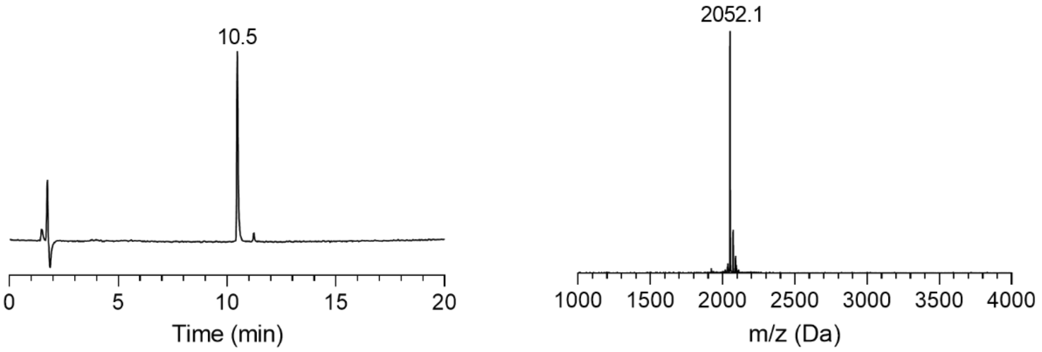

Supplementary figure 23. HPLC trace (220 nm) and MALDI-TOF MS of Shh **Cys^{160}-Gly^{174}-K(PEG)-Biotin**. Column A, gradient 1. Rt = 10.5 min. m/z: 2052.1 $(M+H)^+$, expected: 2052.4.

5.10. Shh Cys^{160}-Gly^{174}-PEG-His$_6$

Synthesis was performed on a Fmoc-Rink-amide resin (0.74 mmol/g, 0.2 mmol scale, Iris Biotech) following the standard Fmoc-SPPS protocols described in the Experimental Section of the manuscript.

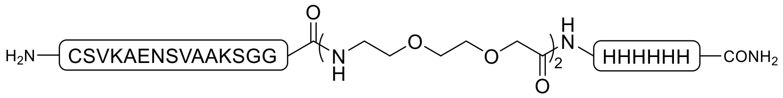

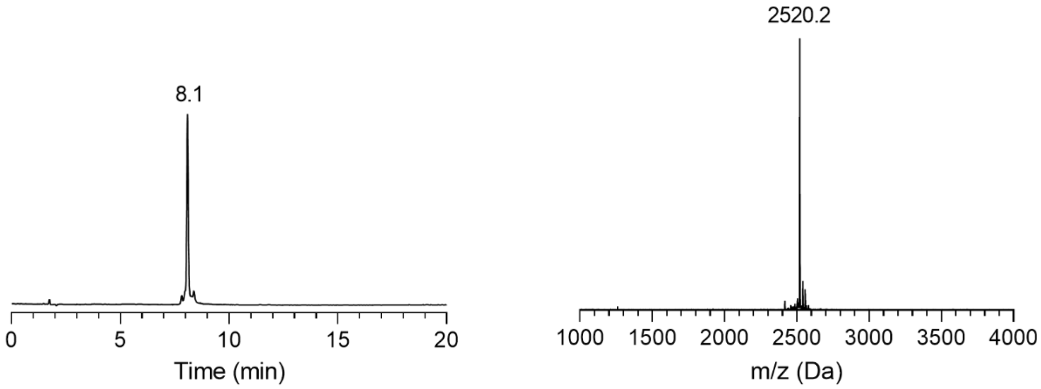

Supplementary figure 24. HPLC trace (220 nm) and MALDI-TOF MS Shh **Cys^{160}-Gly^{174}-PEG-His$_6$**. Column A, gradient 1. Rt = 8.1 min. m/z: 2520.2 $(M+H)^+$,expected: 2520.8.

6. Model peptide ligations

The following experimental procedures typically describe the experimental conditions for one of the replicates. The set of HPLC chromatograms shown in every section (traces at 220 nm) correspond to the described procedure, while the curve fittings for the analyzed products are included for each replicate. For each replicate, aliquots (20 µL) were withdrawn and quenched with the same volume of HCl (0.5 M, 8:2 H2O/CH3CN), and analyzed (5 or 10 µL) by HPLC the same day.

It should be noted that acid quenching induces a side product corresponding to the reaction of TCEP and SeESNa, indicated by an asterisk (*) in the HPLC traces. It does not affect to the kinetic analysis because it is generated after quenching and not during the reaction.

6.1. MS of the ligation products

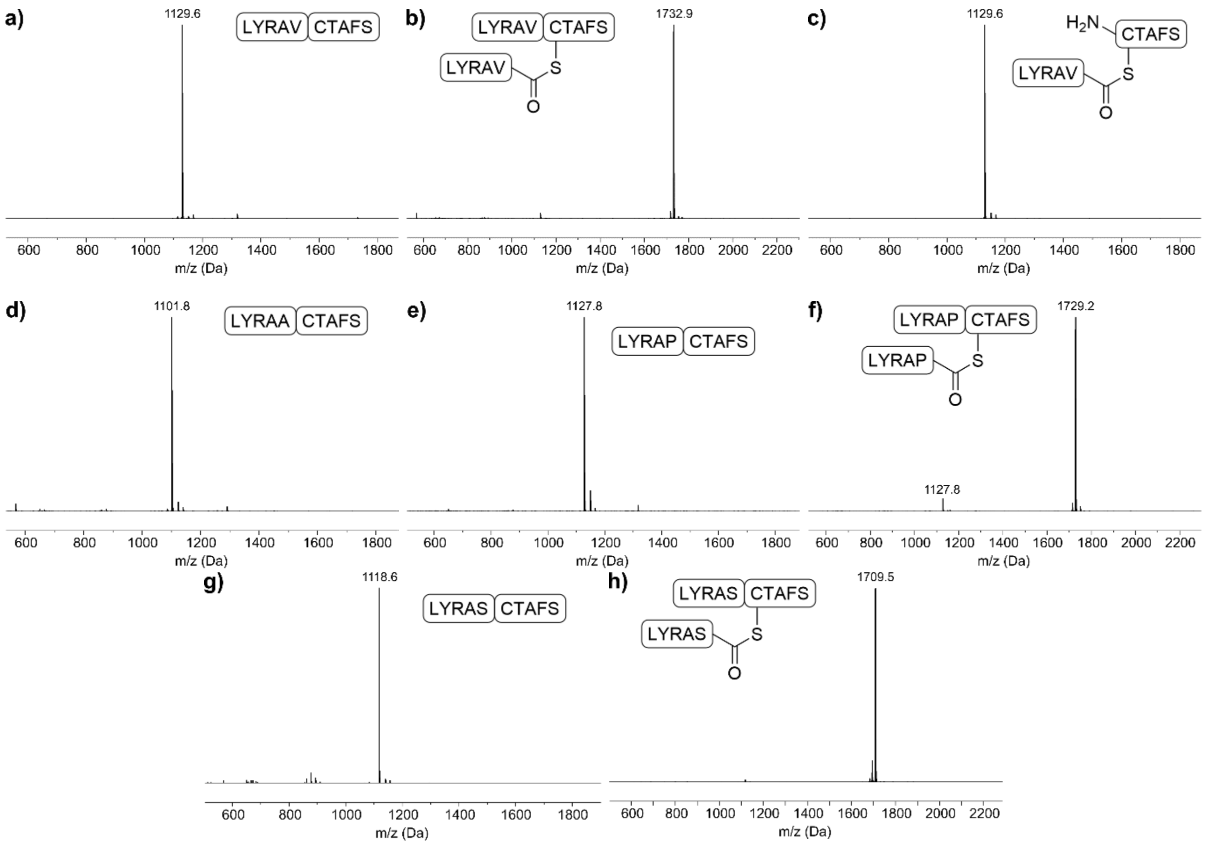

Supplementary figure 25. MALDI-TOF MS of the observed products corresponding to the kinetic experiments: A) **LYRAVCTAFS** (**3**), m/z = 1129.6 (M+H)$^+$, calculated 1130.3. B) **LYRAVC(LYRAV[COS-])TAFS** (**4**), m/z = 1732.9 (M+H)$^+$, calculated 1733.1). C) **LYRAV(COS-)CTAFS** (**3_{int}**), m/z = 1129.6 (M+H)$^+$, calculated: 1130.3. D) **LYRAACTAFS**, m/z = 1101.8 (M+H)$^+$, calculated: 1102.3. E) **LYRAPCTAFS**, m/z = 1127.8 (M+H)$^+$, calculated: 1128.3. F) **LYRAPC(LYRAP[COS-])TAFS**, m/z = 1729.2 (M+H)$^+$, calculated: 1729.1. G) **LYRASCTAFS**, m/z = 1118.6 (M+H)$^+$, calculated: 1119.3. H) **LYRASC(LYRAS[COS-])TAFS**, m/z = 1709.5 (M+H)$^+$, calculated: 1710.0.

6.2. LYRAV-CO(SeESNa) (1), determination of k_1

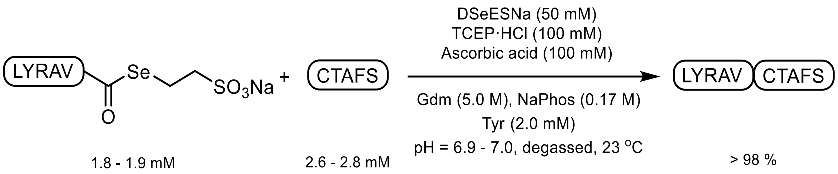

(Exp 1) **1** (1.130 mg, 1.1 x 10^{-3} mmol) and **2** (1.072 mg, 1.7 x 10^{-3} mmol) were weighed in separate polypropylene tubes. In a separate vial, DSeESNa (17.0 mg, 0.03 mmol), TCEP·HCl (17.2 mg, 0.06 mmol) and ascorbic acid (10.6 mg, 0.06 mmol) were dissolved in a mixture of Tyr (20.0 mM, 60 µL) and guanidine / phosphate buffer (500 µL). The pH was adjusted to 7.1 using $NaOH_{(aq)}$ (10 M and 1 M, final volume = 600 µL). The ligation buffer was added to **2**, and the resulting mixture to **1**. The ligation pH, checked at the end of the reaction, was 7.0.

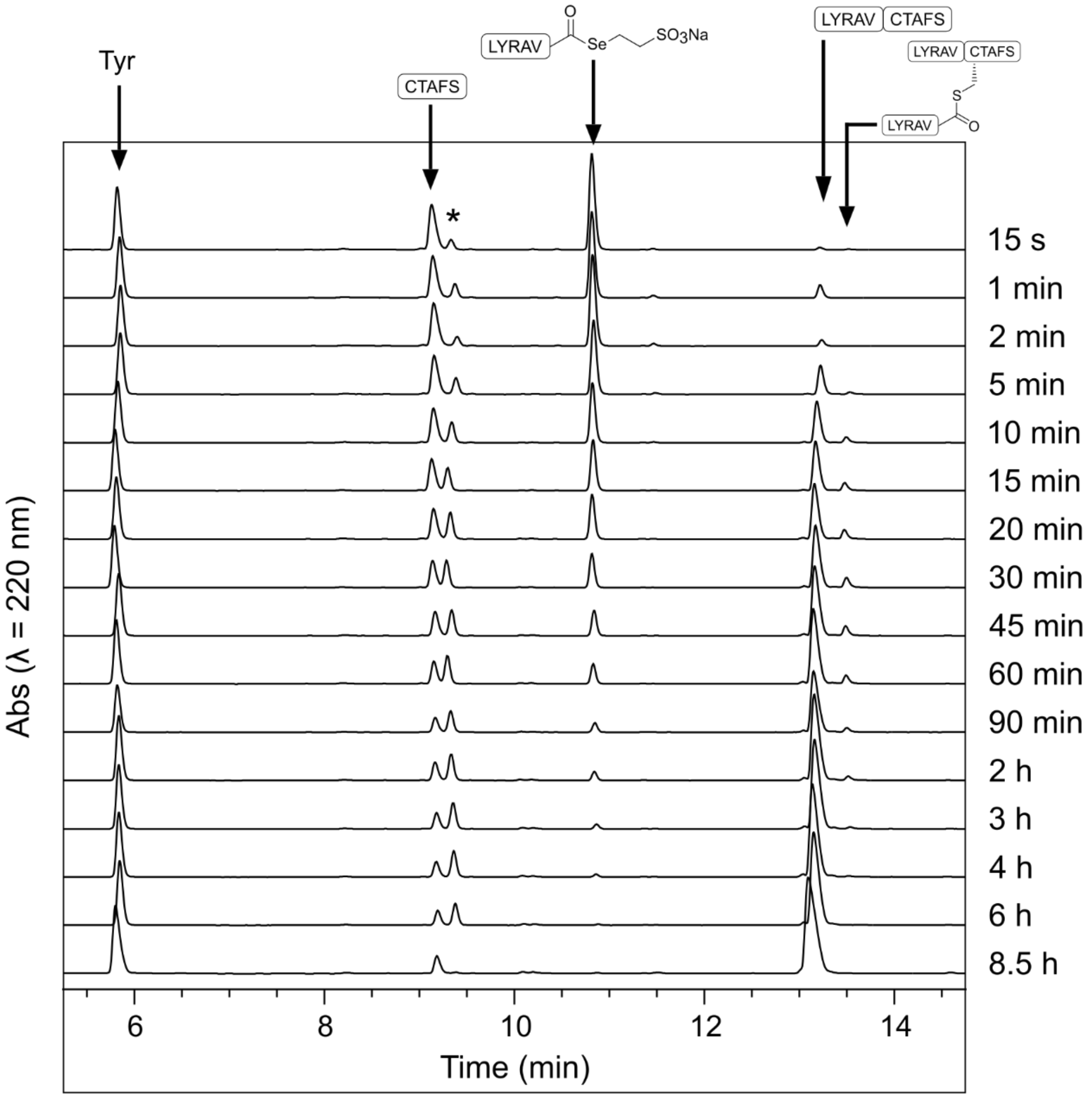

Supplementary figure 26. HPLC traces (220 nm) at given times of the ligation between **1** and **2**, corresponding to Exp 1. Column A, gradient 1. *: TCEP-SeESNa.

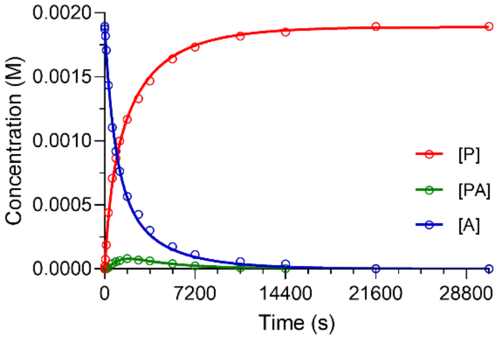

Supplementary figure 27. Experimental points and fitting curves for the ligation between **1** and **2**, corresponding to Exp 1. P = **3**, PA = **4**, A = **1**.

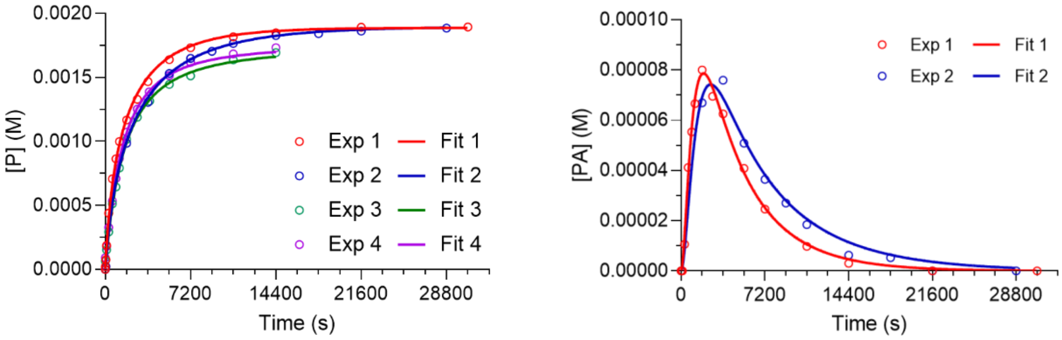

Supplementary figure 28. Experimental points and fitting curves for the replicate ligations between **1** and **2**. P = **3**, PA = **4**.

Supplementary table 4. Calculated rate constants for the ligation between **1** and **2**.

Exp	k_1 (M^{-1} s^{-1})	k_2 (M^{-1} s^{-1})	k_{-2} (M^{-1} s^{-1})
**1**	0.325	0.156	0.0125
**2**	0.230	0.121	0.0110
**3**	0.235	n. d.	n. d.
**4**	0.254	n. d.	n. d.
**Mean**	**0.26 ± 0.04**	**n. d.**	**n. d.**

6.3. LYRAV-CO(SeESNa) (1), determination of k_2 and k_{-2}

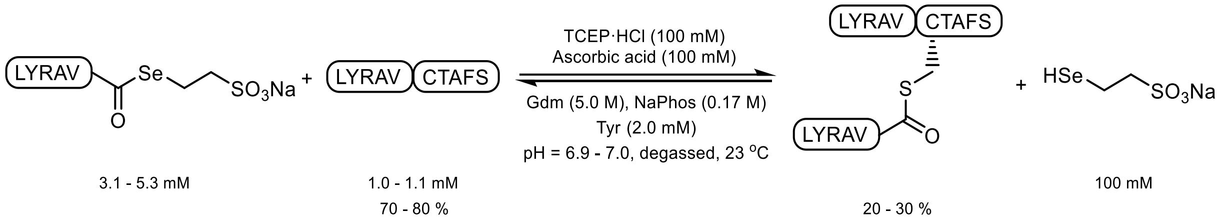

(Exp 1) **1** (3.350 mg, 3.2 x 10^{-3} mmol) and **3** (0.900 mg, 0.60 x 10^{-3} mmol) were weighed in separate polypropylene tubes. In a separate vial, DSeESNa (17.0 mg, 0.03 mmol), TCEP·HCl (17.2 mg, 0.06 mmol) and ascorbic acid (10.6 mg, 0.06 mmol) were dissolved in a mixture of Tyr (20.0 mM, 60 µL) and guanidine / phosphate buffer (500 µL). The pH was adjusted to 7.2 using $NaOH_{(aq)}$ (10 M and 1 M, final volume = 600 µL). The ligation buffer was added to **3**, and the resulting mixture to **1**. The ligation pH, checked at the end of the reaction, was 7.0.

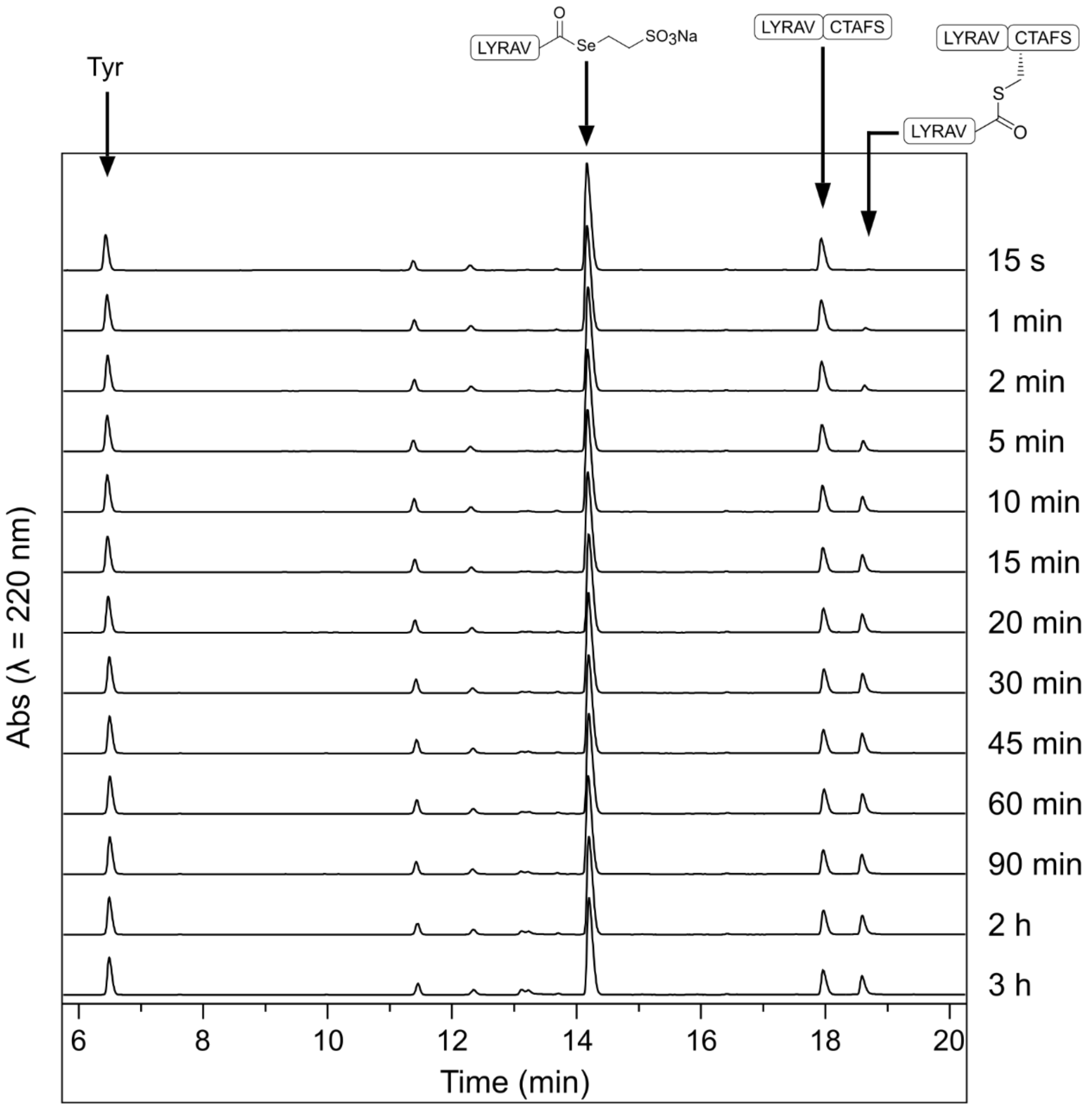

Supplementary figure 29. HPLC traces (220 nm) at given times of the equilibrium between **1**, **3** and **4** corresponding to Exp 1. Column A, gradient 3.

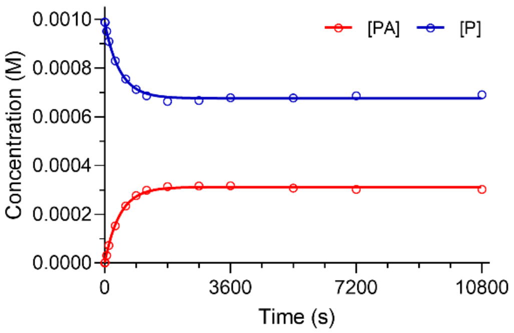

Supplementary figure 30. Experimental points and fitting curves for the equilibrium between **1**, **3** and **4**, corresponding to Exp 1. PA = **4**, P = **3**.

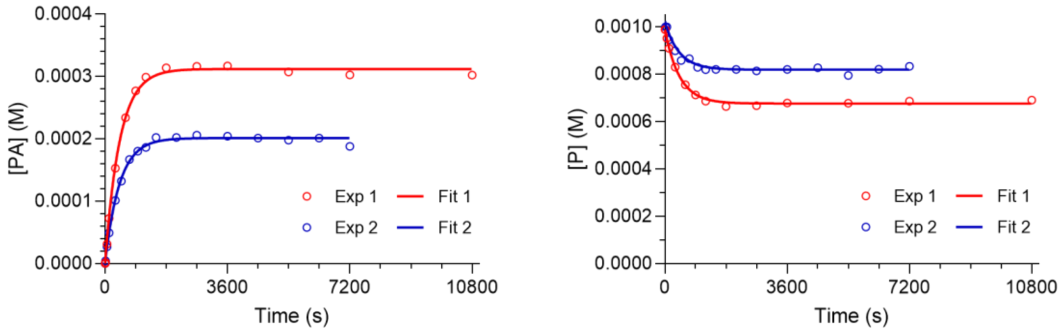

Supplementary figure 31. Experimental points and fitting curves for the equilibrium between **1**, **3** and **4**. PA = **4**, P = **3**.

Supplementary table 5. Calculated k_2, k_{-2}, and k_{eq} for the ligation between **1** and **2**.

Exp	k_2 (M^{-1} s^{-1})	k_{-2} (M^{-1} s^{-1})	K_{eq}
**1**	0.138	0.0149	9.26
**2**	0.151	0.0177	8.53
**1’**	0.156	0.0125	12.48
**2’**	0.121	0.0110	11.00
**Mean**	0.14 ± 0.02	0.014 ± 0.003	10 ± 2

$K_{eq} = k_2 / k_{-2}$. Entries 1’ and 2’ correspond to entries 1 and 2 in Supplementary table 4.

6.4. LYRAV-CO(SeESNa) (1), determination of k_{int}

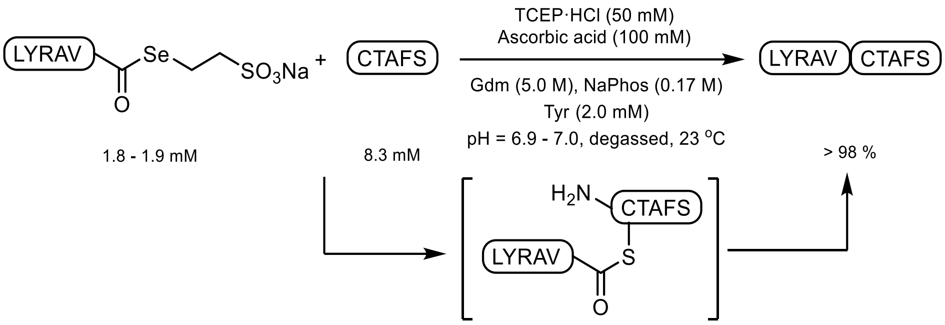

(Exp 3) **1** (1.105 mg, 1.1 x 10^{-3} mmol) and **2** (3.165 mg, 4.9 x 10^{-3} mmol) were weighed in separate polypropylene tubes. In a separate vial, TCEP·HCl (8.6 mg, 0.03 mmol) and ascorbic acid (10.6 mg, 0.06 mmol) were dissolved in a mixture of Tyr (20.0 mM, 60 µL) and guanidine / phosphate buffer (500 µL). The pH was adjusted to 7.05. The ligation buffer was added to **2** and the pH was adjusted to 7.0 (final volume = 600 µL). The resulting mixture was added to **1**. The ligation pH, checked at the end of the reaction, was 7.0.

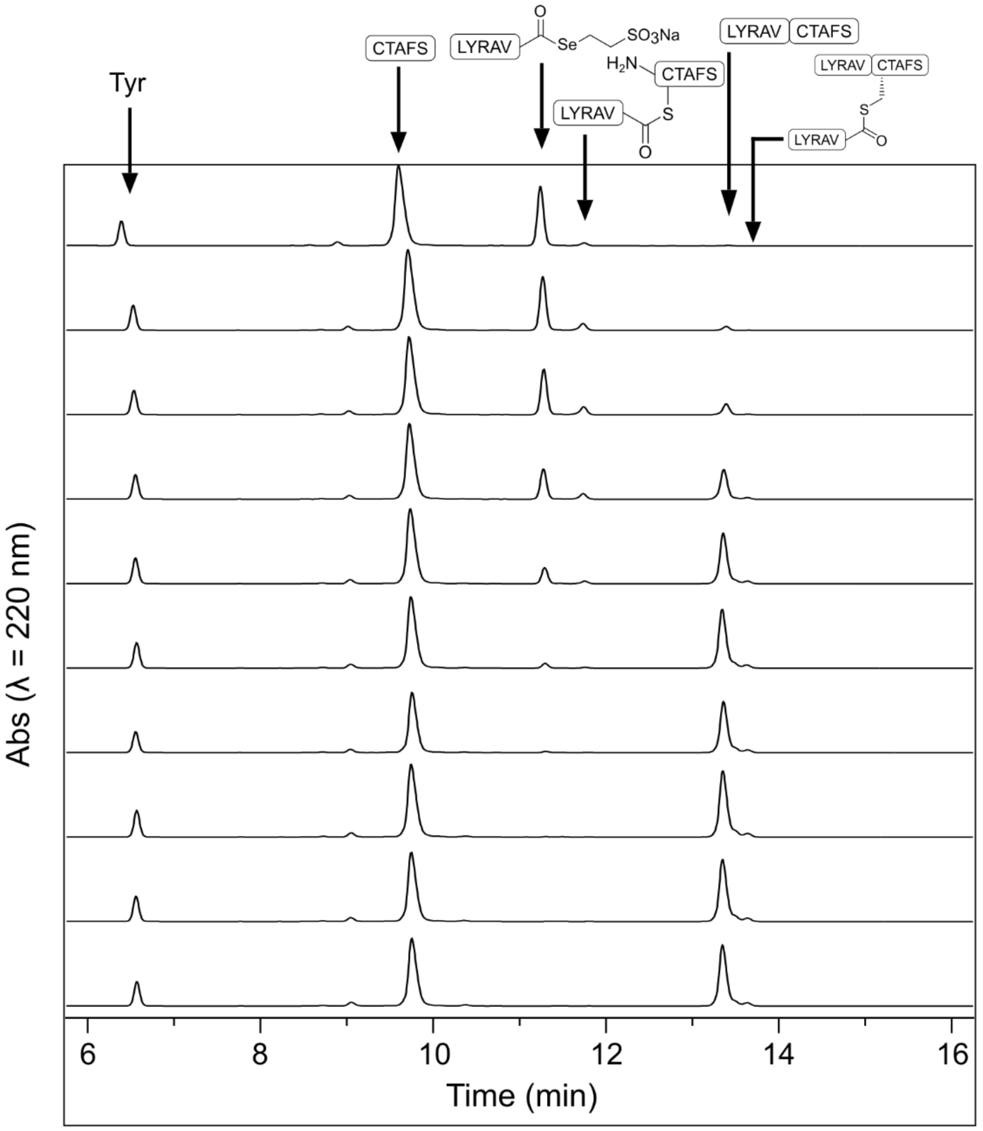

Supplementary figure 32. HPLC traces (220 nm) at given times of the ligation between **1** and **2**, corresponding to Exp 3. Column B, gradient 1.

[**1**]$_t$, [**3$_{int}$**]$_t$, [**3**]$_t$, and [**4**]$_t$ data were analyzed and fitted according to the following kinetic model:

$$\mathbf{1} + \mathbf{2} \xrightarrow{k_1} \mathbf{3_{int}} \xrightarrow{k_{int}} \mathbf{3} + \mathbf{1} \underset{k_{-2}}{\overset{k_2}{\rightleftharpoons}} \mathbf{4} + \mathbf{SeESNa}$$

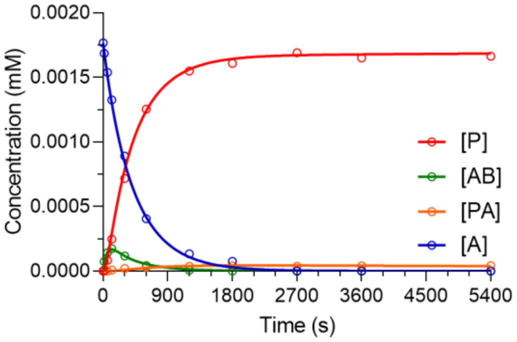

Supplementary figure 33. Experimental points and fitting curves for the ligation between **1** and **2**, corresponding to Exp 3. P = **3**, AB = **3$_{int}$**, PA = **4**, A = **1**.

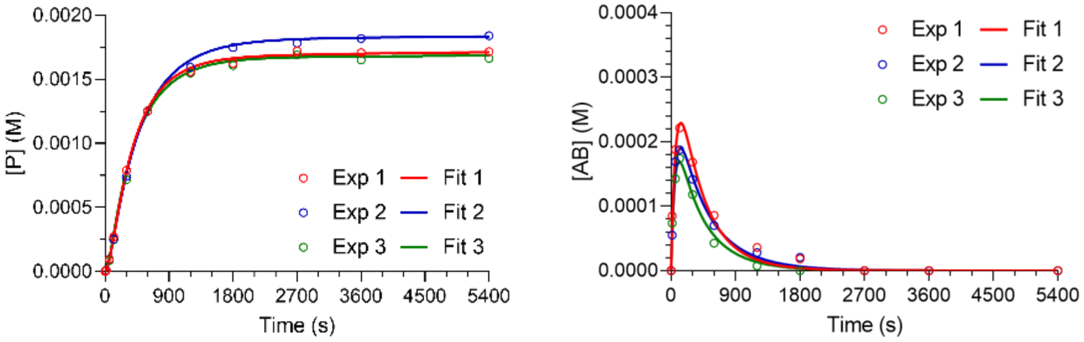

Supplementary figure 34. Experimental points and fitting curves for the replicate ligations between **1** and **2**. P = **3**, AB = **3$_{int}$**.

Supplementary table 6. Calculated k_1 and k_{int} for the ligation between **1** and **2**.

**Exp**	**k_1 (M^{-1} s^{-1})**	**k_{int} (s^{-1})**
**1**	0.345	0.0140
**2**	0.279	0.0162
**3**	0.315	0.0192
**Mean**	**0.31 ± 0.03**	**0.016 ± 0.003**

6.5. LYRAV-CO(4-MPAA)

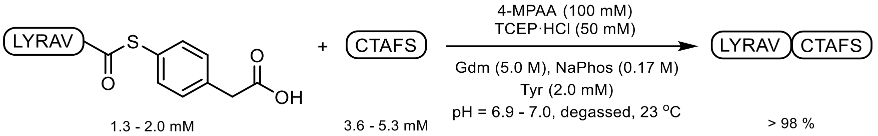

(Exp 3) **LYRAV-CO(4-MPAA)** (1.385 mg, 1.2 x 10^{-3} mmol) and **2** (1.624 mg, 2.5 x 10^{-3} mmol) were weighed in separate polypropylene tubes. In a separate vial, 4-MPAA (10.1 mg, 0.06 mmol) and TCEP·HCl (8.6 mg, 0.03 mmol) were dissolved in a mixture of Tyr (20.0 mM, 60 µL) and guanidine / phosphate buffer (500 µL). The pH was adjusted to 7.1 with $NaOH_{(aq)}$ (10 M and 1 M, final volume = 600 µL). The ligation buffer was added to **2** and the resulting mixture to LYRAV-CO(4-MPAA). The final pH, checked at the end of the reaction, was 7.0.

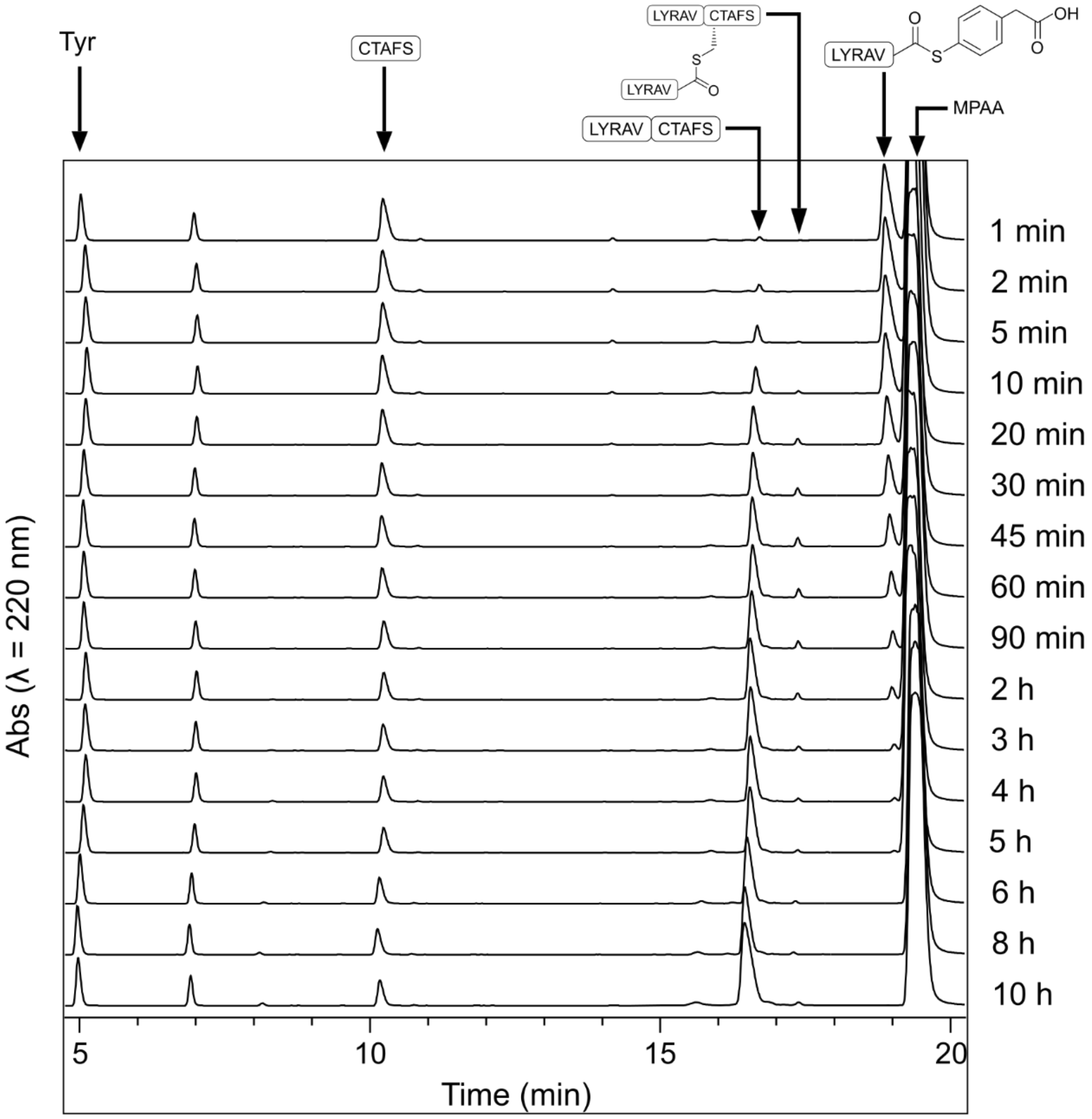

Supplementary figure 35. HPLC traces (220 nm) at given times of the ligation between **LYRAV-CO(4-MPAA)** and **2**, corresponding to Exp 3. Column A, gradient 3.

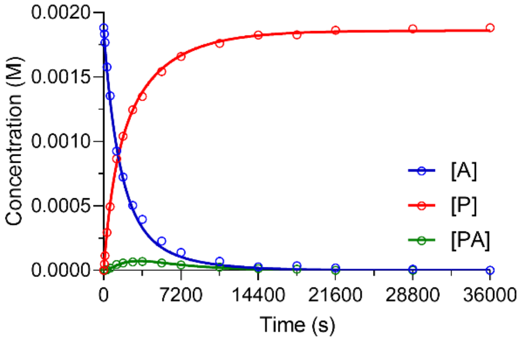

Supplementary figure 36. Experimental points and fitting curves for the ligation between **LYRAV-CO(4-MPAA)** and **2**, corresponding to Exp 3. P = **3**, PA = **4**, A = **LYRAV-COS(4-MPAA)**.

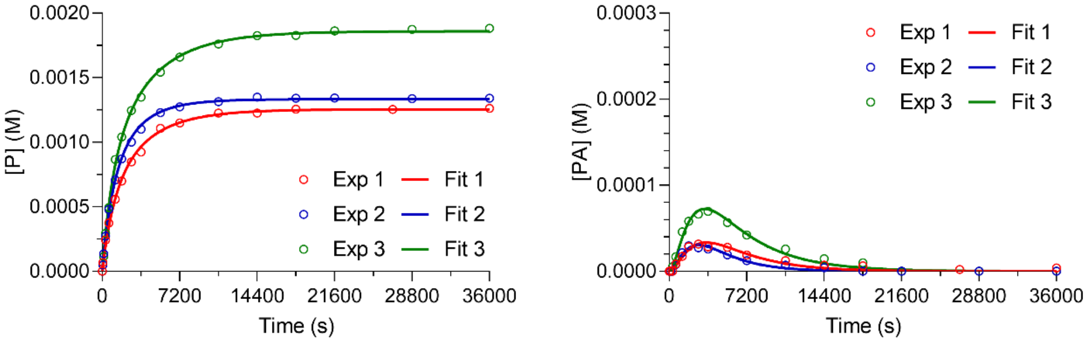

Supplementary figure 37. Experimental points and fitting curves for the replicate ligations between **LYRAV-CO(4-MPAA)** and **2**. P = **3**, PA = **4**.

Supplementary table 7. Calculated rate constants for the ligation between **LYRAV-CO(4-MPAA)** and **2**.

Exp	k_1 (M^{-1} s^{-1})	k_2 (M^{-1} s^{-1})	k_{-2} (M^{-1} s^{-1})
**1**	0.161	0.0628	0.00424
**2**	0.135	0.0621	0.00495
**3**	0.140	0.0689	0.00474
**Mean**	**0.15 ± 0.01**	**0.065 ± 0.003**	**0.0046 ± 0.0003**

6.6. LYRAV-CO(SeESNa) (1), catalyzed by PhSeH

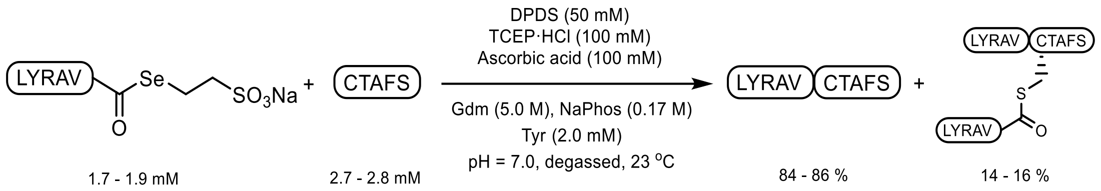

(Exp 1) **1** (1.110 mg, 1.1 x 10^{-3} mmol) and **2** (1.080 mg, 1.7 x 10^{-3} mmol) were weighed in separate polypropylene tubes. In a separate vial, DPDS (9.36 mg, 0.03 mmol), TCEP·HCl (17.2 mg, 0.06 mmol) and ascorbic acid (10.6 mg, 0.06 mmol) were dissolved in a mixture of Tyr (20.0 mM, 60 µL) and guanidine / phosphate buffer (500 µL). The pH was adjusted to 7.1 with $NaOH_{(aq)}$ (10 M and 1 M, (final volume = 600 µL). The ligation buffer was added to **2**, and the resulting mixture to **1**. The final pH, checked at the end of the reaction, was 7.0.

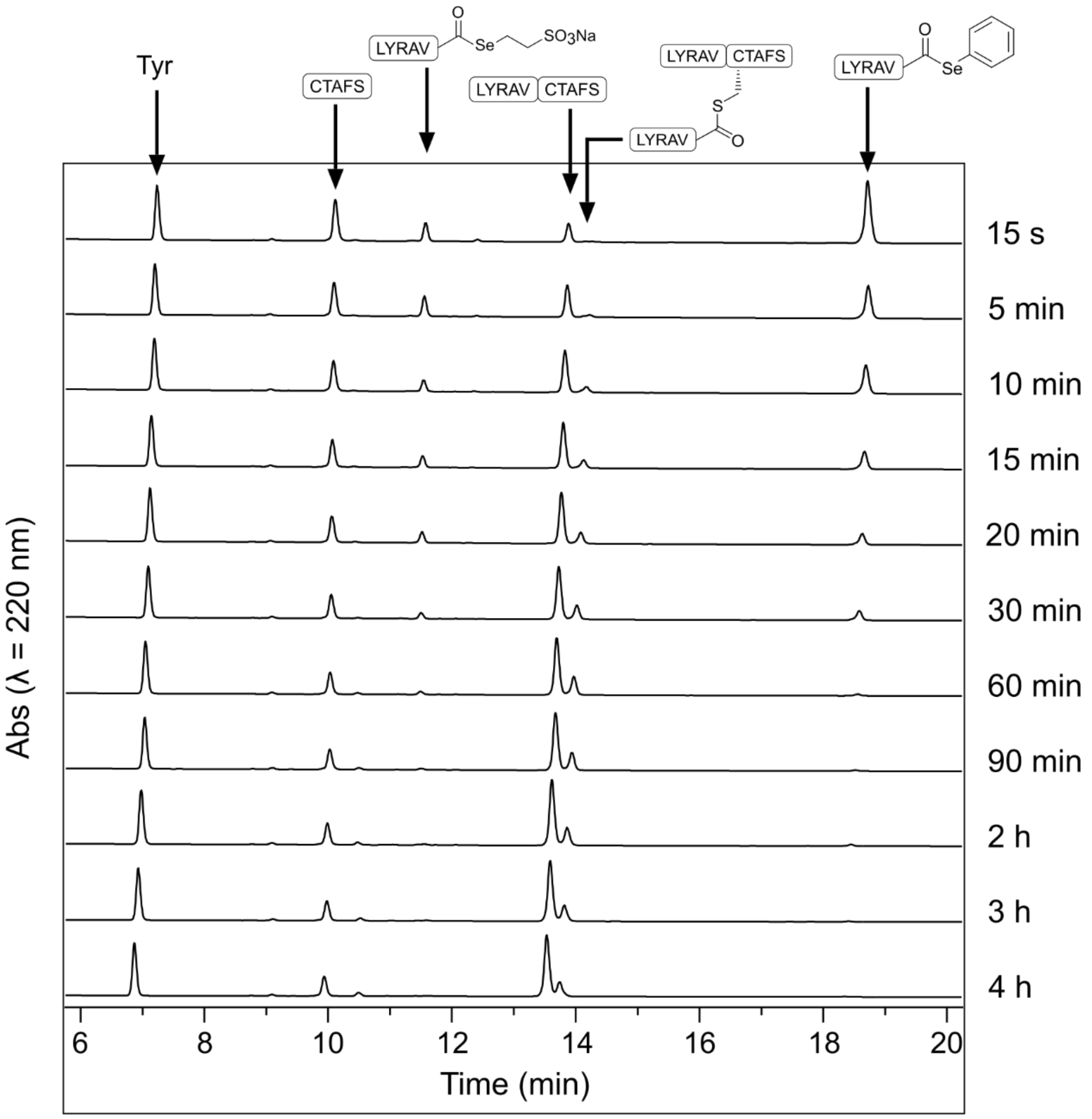

Supplementary figure 38. HPLC traces (220 nm) at given times of the ligation between **1** and **2**, catalyzed by DPDS. Column B, gradient 1.

[**3**]$_t$ and [**4**]$_t$ data were analyzed and fitted according to the following kinetic model:

$$\mathbf{1} + \mathbf{2} \xrightarrow{k_{obs}} \mathbf{3} + \mathbf{1} \xrightarrow{k_{obs.branch}} \mathbf{4}$$

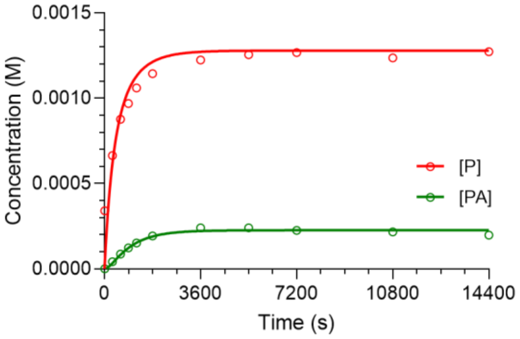

Supplementary figure 39. Experimental points and fitted curves for the peptides in the HPLC traces of the ligation of **1** and **2**, corresponding to Exp 1. P = **3**, PA = **4**.

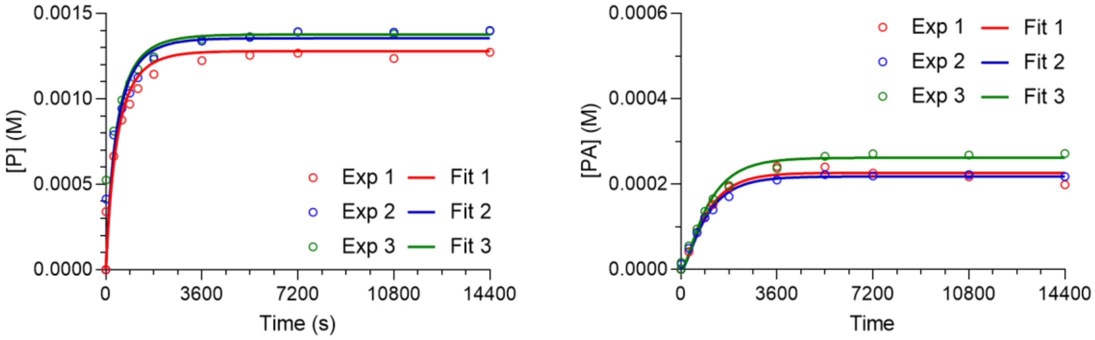

Supplementary figure 40. Experimental points and fitting curves for the replicate ligations between **1** and **2**. P = **3**, PA = **4**.

Supplementary table 8. Calculated rate constants for the ligation between **1** and **2** catalyzed by phenylselenol.

Exp	k_{obs} (M^{-1} s^{-1})	$k_{obs.branch}$ (M^{-1} s^{-1})
**1**	0.654	0.253
**2**	0.756	0.234
**3**	0.733	0.225
**Mean**	**0.71 ± 0.05**	**0.24 ± 0.01**

6.7. LYRAV-CO(MESNa) (5), catalyzed by SeESNa

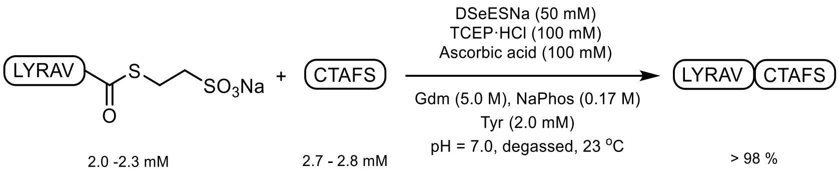

(Exp 2) **5** (1.262 mg, 1.2 x 10^{-3} mmol) and **2** (1.077 mg, 1.7 x 10^{-3} mmol) were weighed in separate polypropylene tubes. In a separate vial, DSeESNa (17.0 mg, 0.03 mmol), TCEP·HCl (17.2 mg, 0.06 mmol) and ascorbic acid (10.6 mg, 0.06 mmol) were dissolved in a mixture of Tyr (20.0 mM, 60 µL) and guanidine / phosphate buffer (500 µL). The pH was adjusted to 7.1 (final volume = 600 µL), and the ligation buffer was added to **2**. The resulting mixture was added to **5**. The ligation pH, checked at the end of the reaction, was 7.0.

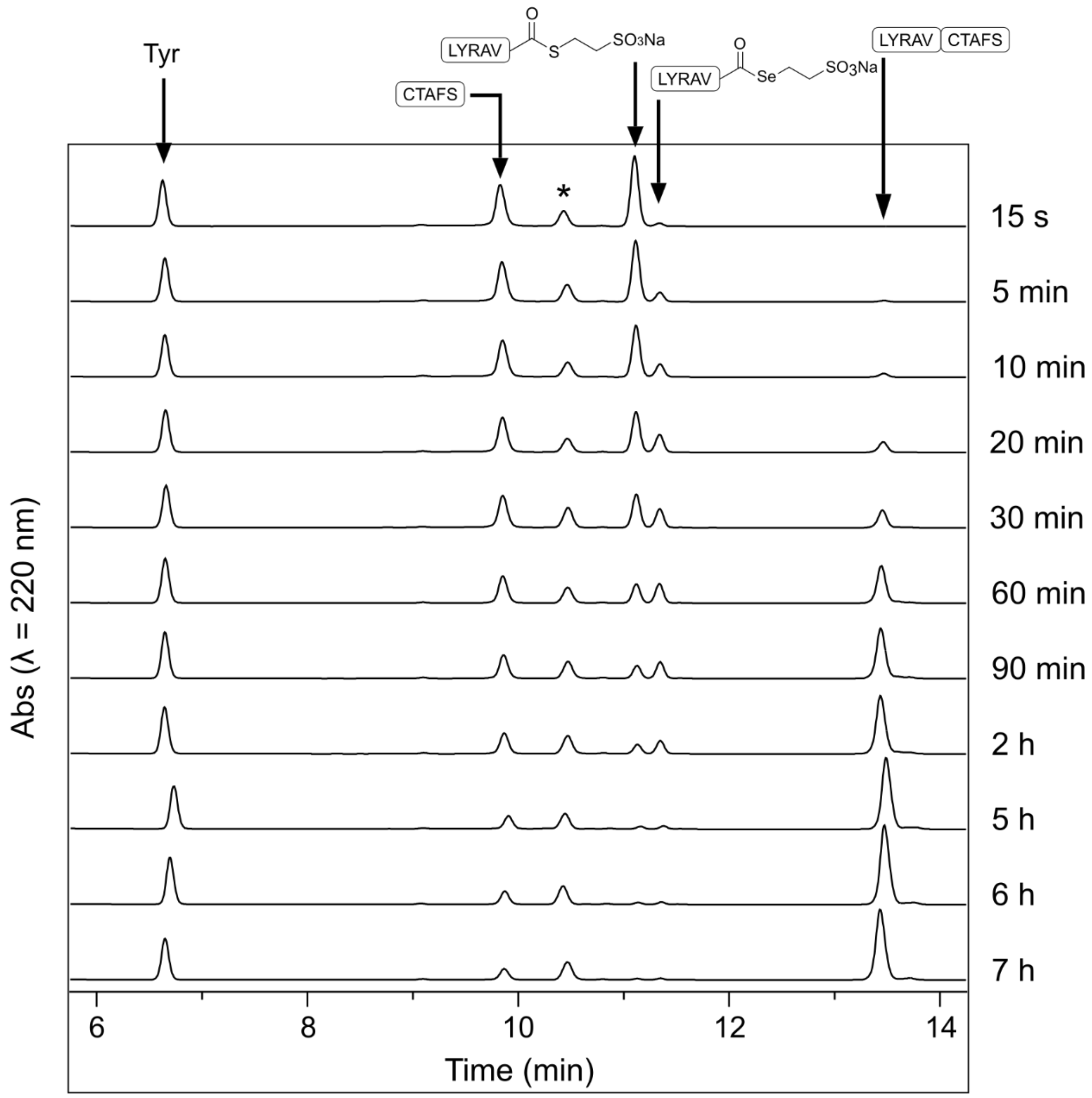

Supplementary figure 41. HPLC traces (220 nm) at given times of the ligation between **5** and **2**, catalyzed by SeESNa. Column B, gradient 1. *: TCEP-SeSNa.

[**3**]$_t$ and [**4**]$_t$ data were analyzed and fitted according to the following kinetic model:

$$\mathbf{5} + \mathbf{SeESNa} \underset{k_{Se\rightarrow S}}{\overset{k_{S\rightarrow Se}}{\rightleftharpoons}} \mathbf{1} + \mathbf{MESNa}$$

$$\mathbf{1} + \mathbf{2} \xrightarrow{k_1 = 0.26\ M^{-1}s^{-1}} \mathbf{3} + \mathbf{1} \underset{k_{-2} = 0.014\ \mathrm{M^{-1}s^{-1}}}{\overset{k_2 = 0.14\ \mathrm{M^{-1}s^{-1}}}{\rightleftharpoons}} \mathbf{4} + \mathbf{SeESNa}$$

In where the constants previously calculated (k_1, k_2, and k_{-2}) were fixed, and $k_{S\rightarrow Se}$ and $k_{Se\rightarrow S}$ estimated.

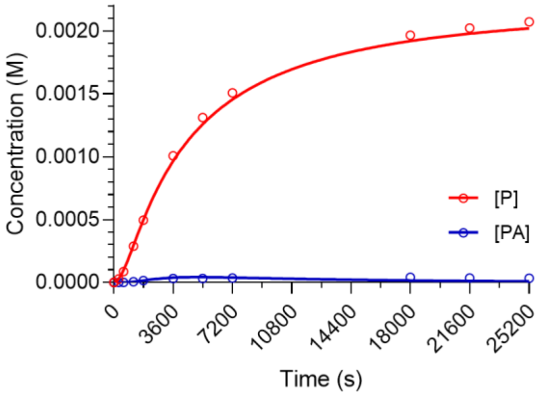

Supplementary figure 42. Experimental points and fitted curves for the peptides in the HPLC traces of the ligation of **5** and **2**, corresponding to Exp 2. P = **3**, PA = **4**.

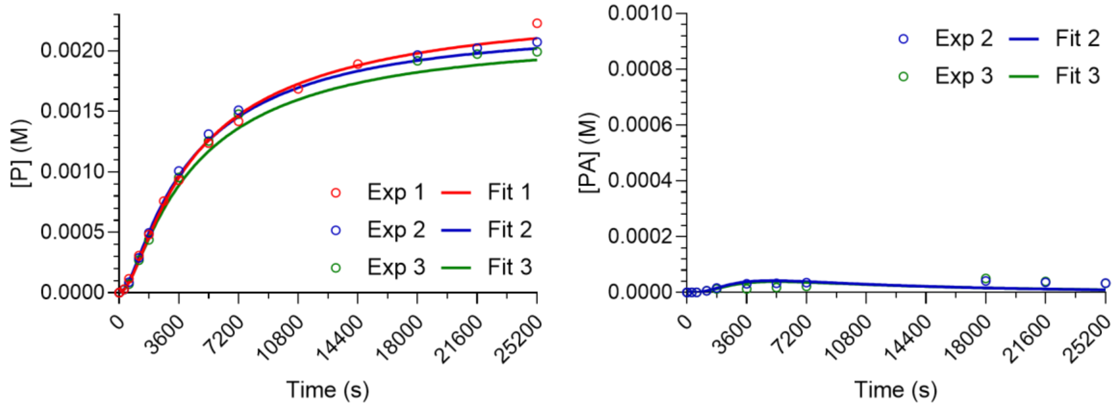

Supplementary figure 43. Experimental points and fitting curves for the replicate ligations between **5** and **2**. P = **3**, PA = **4**.

Supplementary table 9. Calculated rate constants for the ligation between **5** and **2** catalyzed by SeESNa.

Exp	$k_{S\rightarrow Se}$ ($M^{-1}\ s^{-1}$)	$k_{Se\rightarrow S}$ ($M^{-1}\ s^{-1}$)
**1**	0.00371	0.284
**2**	0.00466	0.247
**3**	0.00437	0.255
**Mean**	**0.0043 ± 0.0005**	**0.26 ± 0.02**

6.8. LYRAV-CO(SeESNa) (1) equilibrium with MESNa, determination of $k_{Se\rightarrow S}$ and $k_{S\rightarrow Se}$

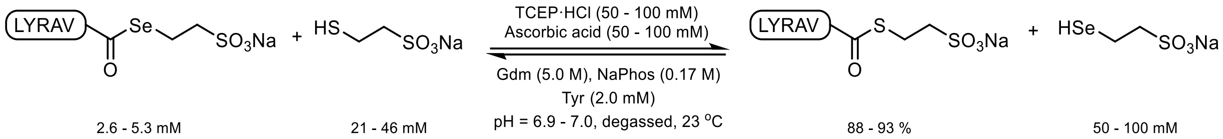

(Exp 1) **1** (3.305 mg, 3.2 x 10^{-3} mmol) was weighed in a polypropylene tube and, in a separate tube, MESNa (4.95 mg, 0.027 mmol), DSeESNa (17.0 mg, 0.03 mmol). In a third different vial, TCEP·HCl (17.2 mg, 0.06 mmol) and ascorbic acid (10.6 mg, 0.06 mmol) were dissolved in a mixture of Tyr (20.0 mM, 60 µL) and guanidine / phosphate buffer (500 µL). The pH was adjusted to 7.1 using $NaOH_{(aq)}$ (10 M and 1 M, final volume = 600 µL). The ligation buffer was first added to the MESNa + DSeESNa mixture and, finally, to **5**. The pH, checked at the end of the reaction, was 7.0.

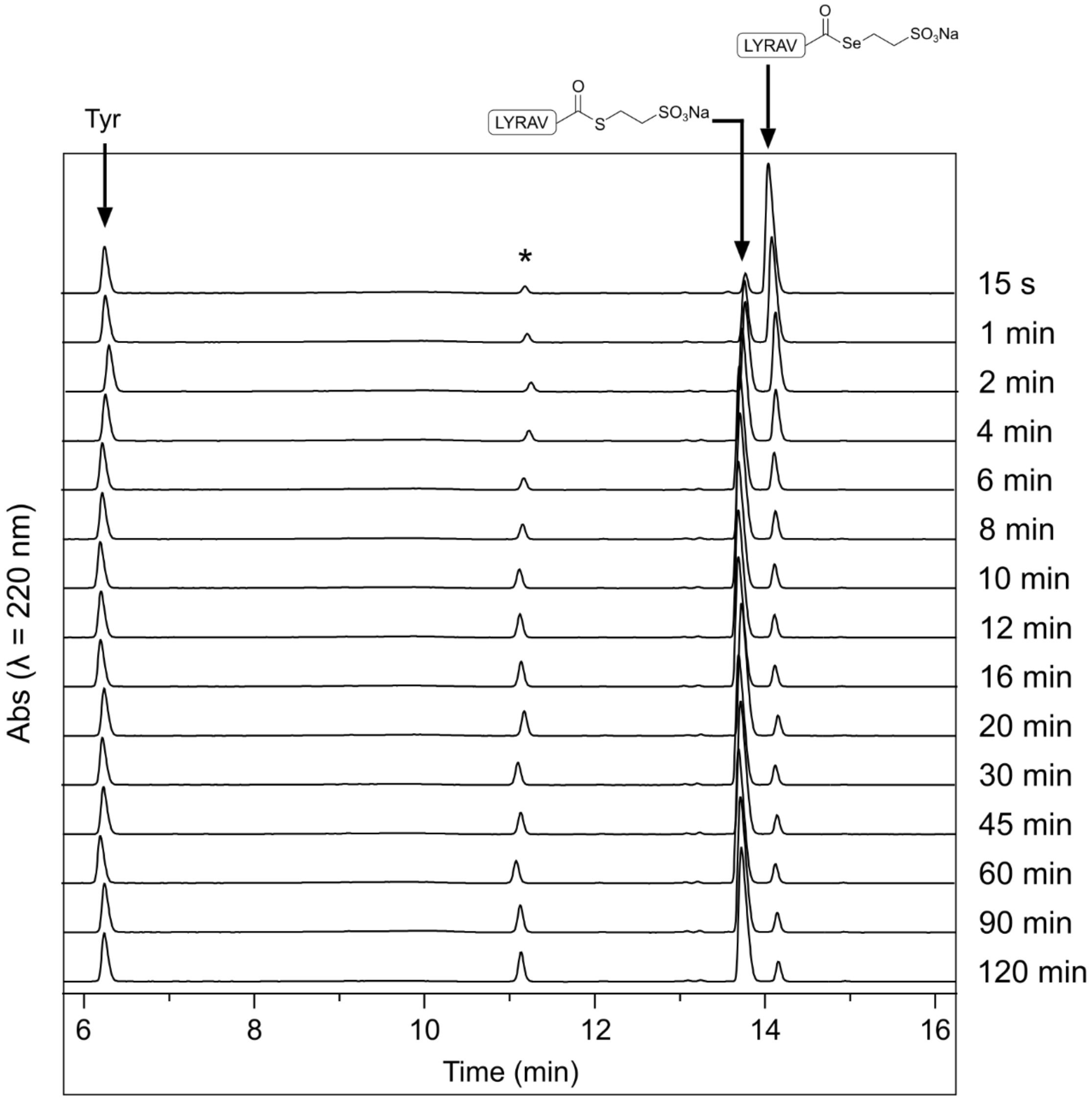

Supplementary figure 44. HPLC traces (220 nm) at given times of the equilibrium between **5** and **1**. Column A, gradient 3. *: TCEP-SeESNa.

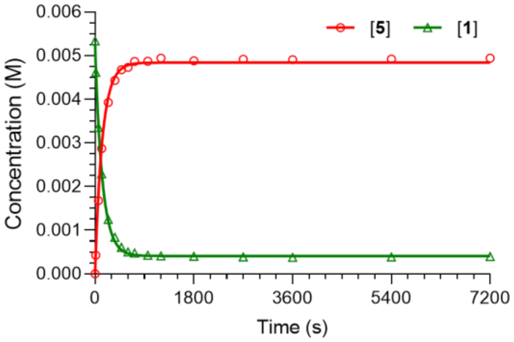

Supplementary figure 45. Experimental points and fitting curves for the equilibrium between **5** and **1**, corresponding to Exp 1.

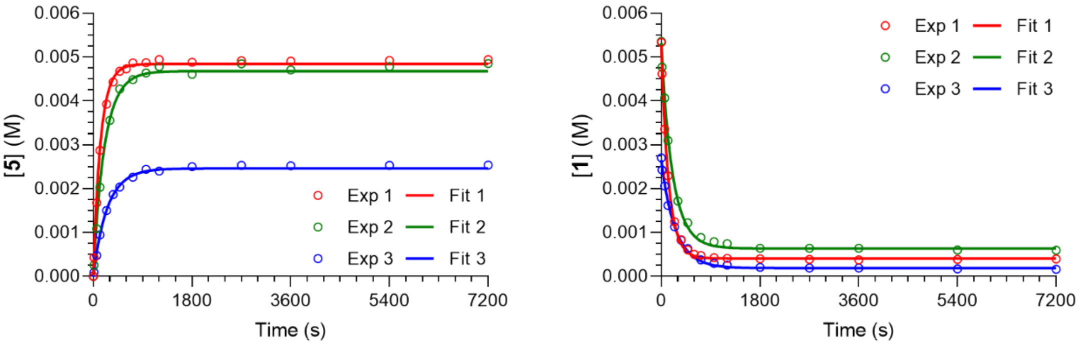

Supplementary figure 46. Experimental points and fitting curves for the replicate equilibriums between **5** and **1**.

Supplementary table 10. Calculated rate constants for the equilibrium between **5** and **1**.

Exp	$k_{Se \rightarrow S}$ (M^{-1} s^{-1})	$k_{S \rightarrow Se}$ (M^{-1} s^{-1})	K_{eq}
**1**	0.158	0.00523	30.1
**2**	0.184	0.00485	37.8
**3**	0.181	0.00487	37.2
**Mean**	**0.17 ± 0.01**	**0.0050 ± 0.0002**	**35 ± 3**

6.9. LYRAVC(LYRAV[COS-])TAFS (4), catalyzed by PhSeH

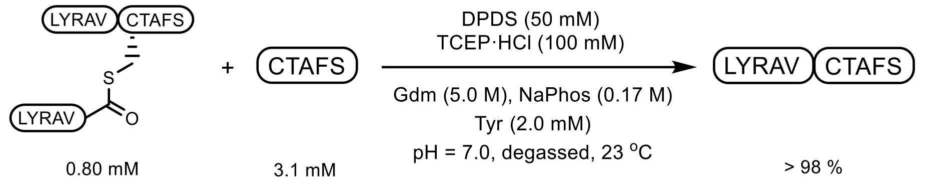

4 (1.180 mg, 4.8 x 10^{-4} mmol) and **2** (1.170 mg, 1.8 x 10^{-3} mmol) were weighed in separate polypropylene tubes. In a separate vial, DPDS (15.6 mg, 0.05 mmol), TCEP·HCl (28.6 mg, 0.1 mmol) were dissolved in a mixture of Tyr (20.0 mM, 100 µL) and guanidine / phosphate buffer (833 µL). The pH was adjusted to 7.1. The final volume was brought to 1.0 mL with H_2O to form the ligation buffer. 0.600 mL of this buffer was added to **2**, and the resulting mixture was added to **4**. The ligation pH, checked after 9 h, was 7.0.

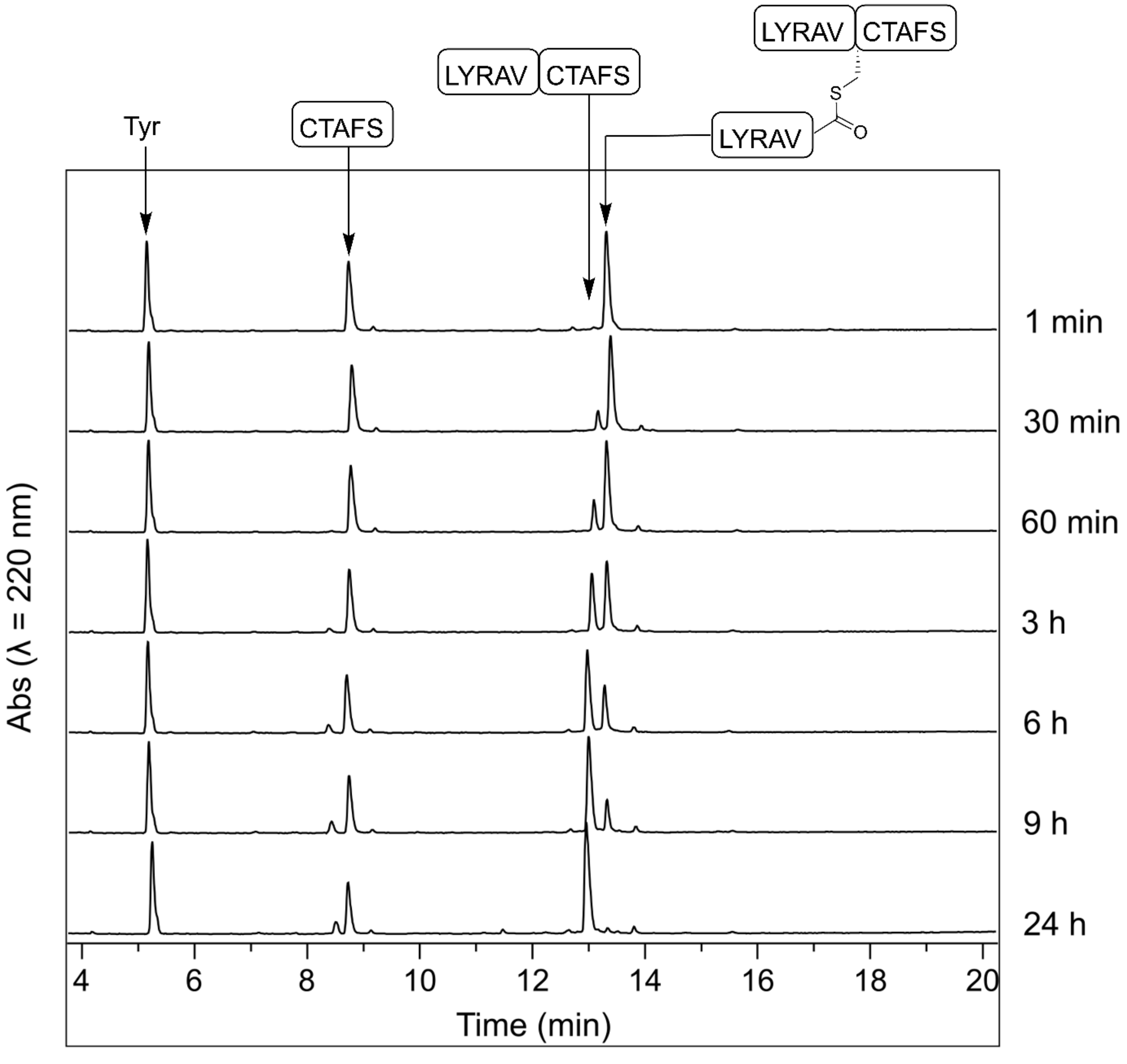

Supplementary figure 47. HPLC traces (220 nm) at given times of the ligation between **4** and **2**, catalyzed by phenylselenol. Column B, gradient 1.

6.10. LYRAV-CO(MESNa) (5), catalyzed by PhSeH

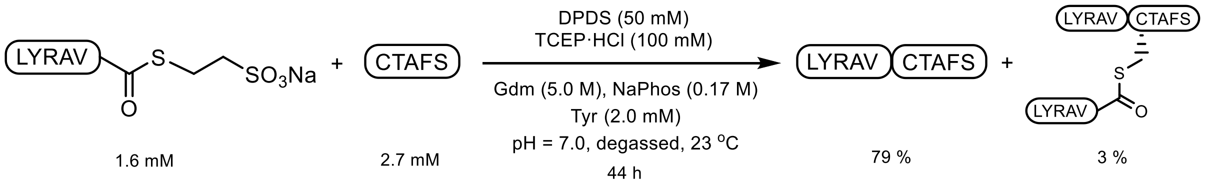

5 (1.040 mg, 9.9 x 10^{-4} mmol) and **2** (1.024 mg, 1.6 x 10^{-3} mmol) were weighed in separate polypropylene tubes. In a separate vial, DPDS (15.6 mg, 0.05 mmol), TCEP·HCl (28.6 mg, 0.1 mmol) were dissolved in a mixture of Tyr (20.0 mM, 100 µL) and guanidine / phosphate buffer (833 µL). The pH was adjusted to 7.1. The final volume was brought to 1.0 mL with H_2O to form the ligation buffer. 0.600 mL of this buffer was added to **2**, and the resulting mixture was added to **5**. The ligation pH, checked after 3 h, was 7.0.

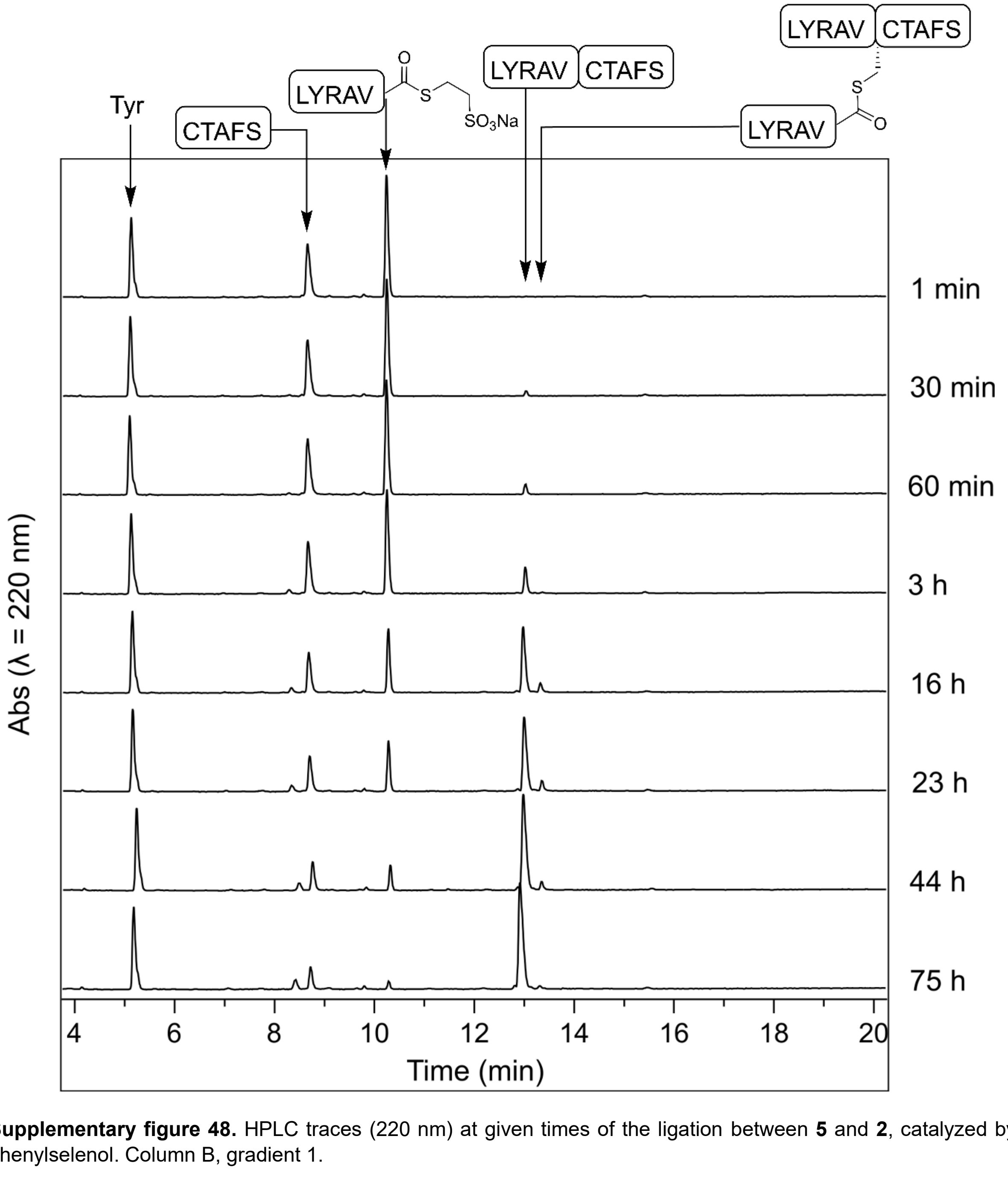

Supplementary figure 48. HPLC traces (220 nm) at given times of the ligation between **5** and **2**, catalyzed by phenylselenol. Column B, gradient 1.

6.11. LYRAV-CO(Nbz)-G (6), catalyzed by SeESNa

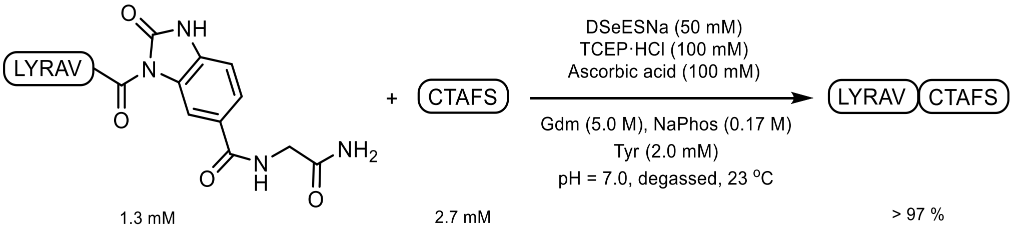

(Exp 3) **6** (1.215 mg, 7.8 x 10^{-4} mmol) and **2** (1.047 mg, 1.6 x 10^{-3} mmol) were weighed in separate polypropylene tubes. In a separate vial, DSeESNa (17.0 mg, 0.03 mmol), TCEP·HCl (13.9 mg, 0.06 mmol) and ascorbic acid (10.6 mg, 0.06 mmol) were dissolved in a mixture of Tyr (20.0 mM, 60 µL) and guanidine / phosphate buffer (500 µL). The pH was adjusted to 7.1, and the ligation buffer added to **2** (final volume = 600 µL). The resulting mixture was added to **6**. The ligation pH, checked at the end of the reaction, was 7.0.

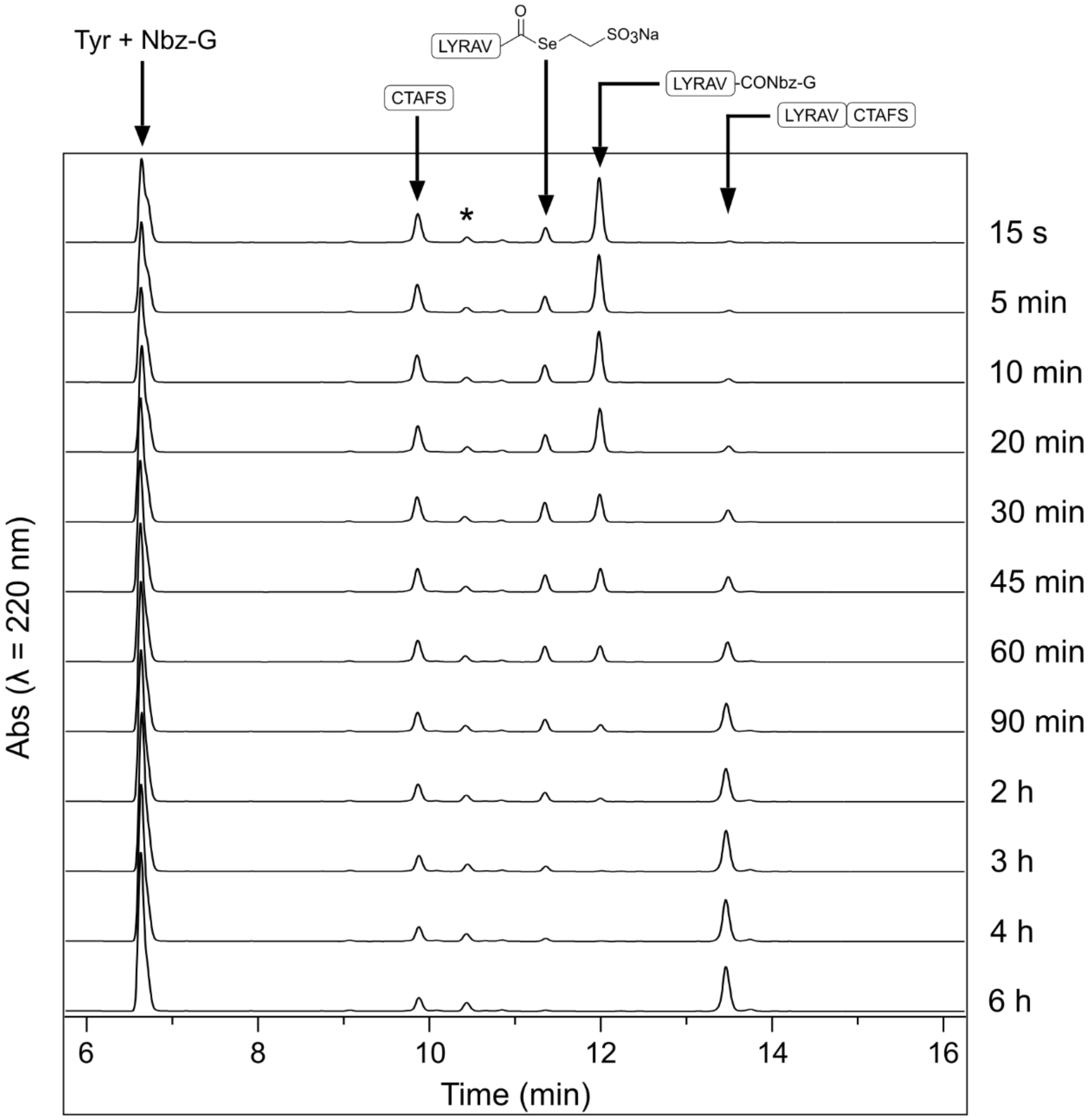

Supplementary figure 49. HPLC traces (220 nm) at given times of the ligation between **6** and **2**, catalyzed by SeESNa. Column B, gradient 1. *: TCEP-SeESNa.

Note that Nbz-G coelutes with Tyr, and so the determination of $[\mathbf{3}]_t$ and $[\mathbf{4}]_t$ resulted inaccurate. To estimate $[\mathbf{3}]_t$ and $[\mathbf{4}]_t$, the areas at 280 nm of **3** (obtained from the different kinetic experiments involving LYRAV-CO(XR) + CTAFS ligations) were plotted with their corresponding [**3**]. A lineal fit correlation was obtained using the following expression: $[\mathbf{3}] = k \times (area_{280nm}) + c$. $[\mathbf{3}]_t$ was estimated using the regression line, and $[\mathbf{4}]_t$ was calculated from the same equation dividing by 2 (absorption of 2 Tyr residues).

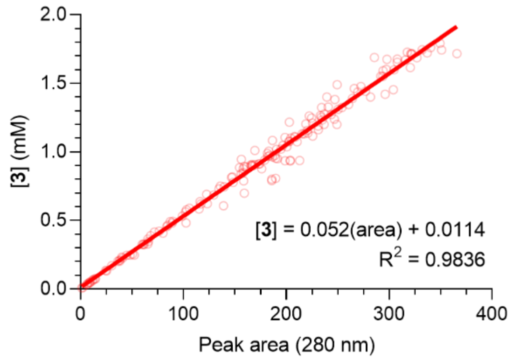

Supplementary figure 50. Regression line for the calculation of [**3**] and [**4**] in the ligation between **6** and **2**.

$[\mathbf{3}]_t$ and $[\mathbf{4}]_t$ data were analyzed and fitted according to the following kinetic model (k_1, k_2, and k_{-2} were fixed, and $k_{Nbz\text{->}Se}$ estimated):

$$\mathbf{6} + \mathbf{SeESNa} \xrightarrow{k_{Nbz\rightarrow Se}} \mathbf{1} + \mathbf{Nbz-G}$$

$$\mathbf{1} + \mathbf{2} \xrightarrow{k_1 = 0.26\ M^{-1}s^{-1}} \mathbf{3} + \mathbf{1} \underset{k_{-2}=0.014\ M^{-1}s^{-1}}{\overset{k_2=0.14\ M^{-1}s^{-1}}{\rightleftharpoons}} \mathbf{4} + \mathbf{SeESNa}$$

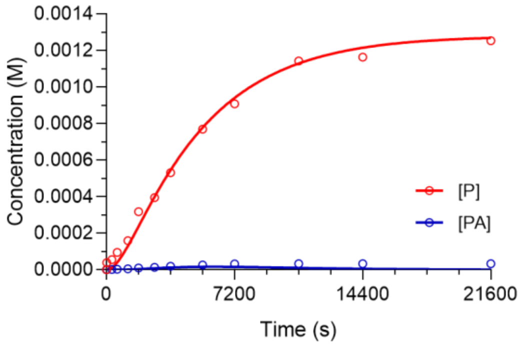

Supplementary figure 51. Experimental points and fitting curves for the ligation between **6** and **2**, corresponding to Exp 3. P = **3**, PA = **4**.

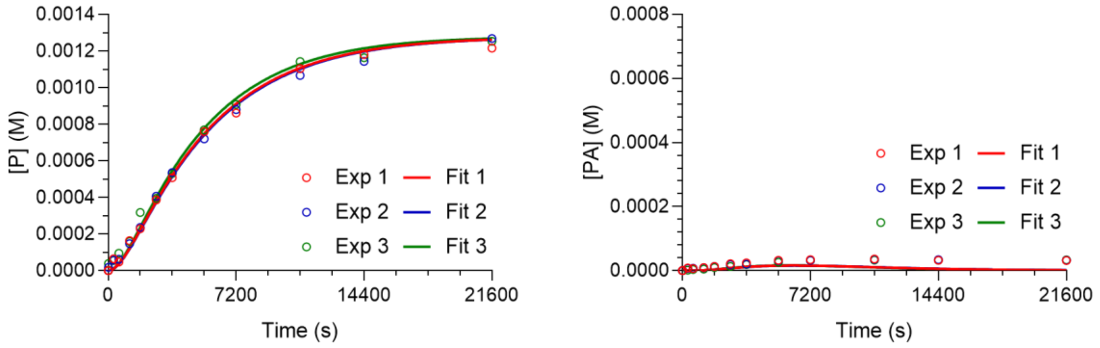

Supplementary figure 52. Experimental points and fitting curves for the replicate ligations between **6** and **2**. P = **3**, PA = **4**.

Supplementary table 11. Calculated rate constants for the ligation between **6** and **2** catalyzed by SeESNa.

Exp	$k_{Nbz\text{->}Se}$ (M^{-1} s^{-1})
**1**	0.00283
**2**	0.00278
**3**	0.00300
**Mean**	**0.0029 ± 0.0001**

6.12. LYRAA-CO(SeESNa)

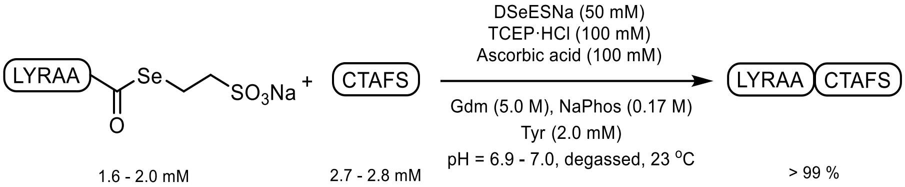

(Exp 1) **LYRAA-CO(SeESNa)** (1.005 mg, 9.6 x 10^{-4} mmol) and **2** (1.030 mg, 1.6 x 10^{-3} mmol) were weighed in separate polypropylene tubes. In a separate vial, DSeESNa (17.0 mg, 0.03 mmol), TCEP·HCl (17.2 mg, 0.06 mmol) and ascorbic acid (10.6 mg, 0.06 mmol) were dissolved in a mixture of Tyr (20.0 mM, 60 µL) and guanidine / phosphate buffer (500 µL). The pH was adjusted to 7.05 (final volume = 600 µL), and the ligation buffer added to **2**. The resulting mixture was added to LYRAA-CO(SeESNa). The final pH, checked at the end of the reaction, was 7.0.

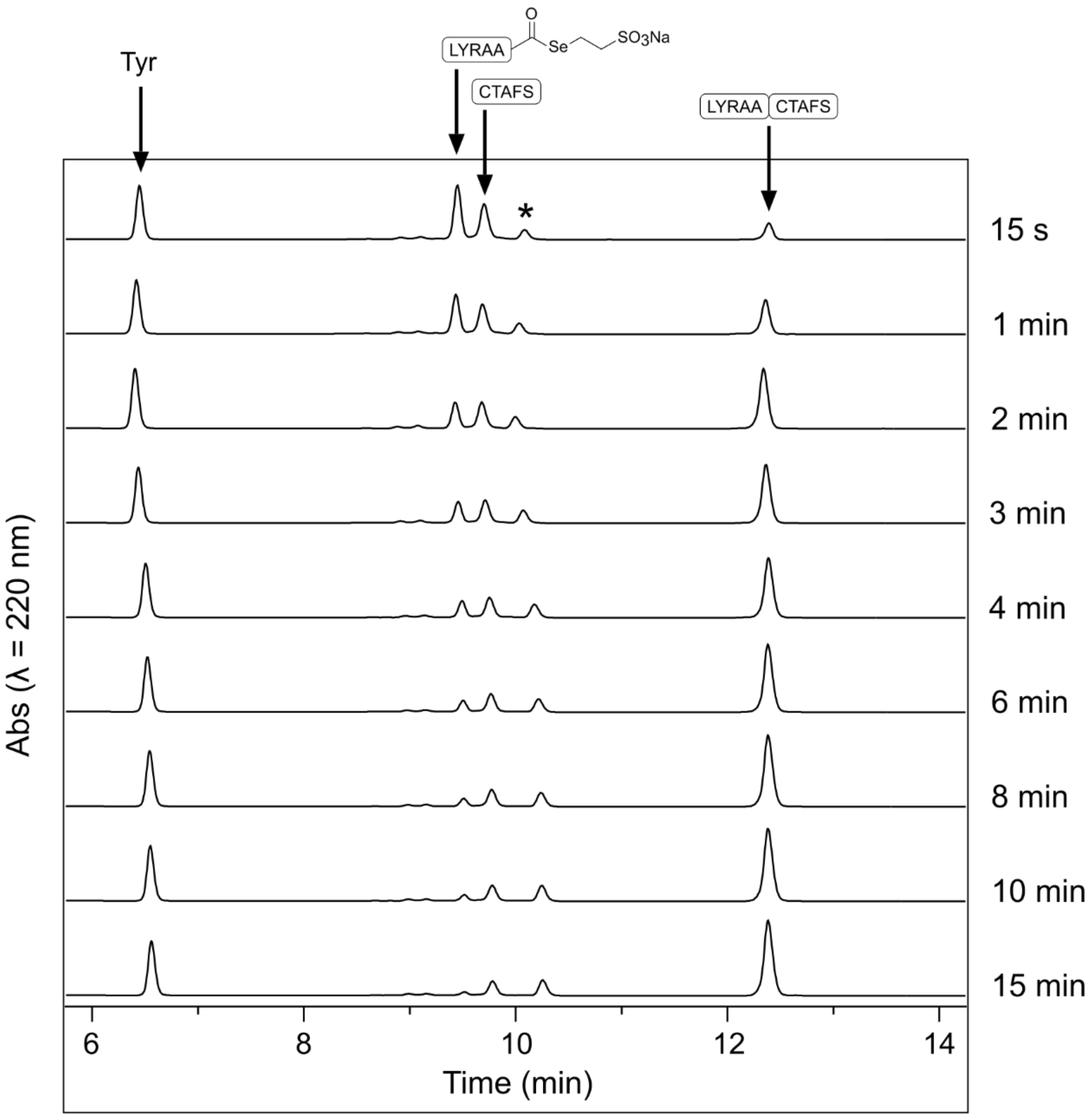

Supplementary figure 53. HPLC traces (220 nm) at given times of the ligation between **LYRAA-CO(SeESNa)** and **2**, corresponding to Exp 1. Column B, gradient 1. *: TCEP-SeESNa.

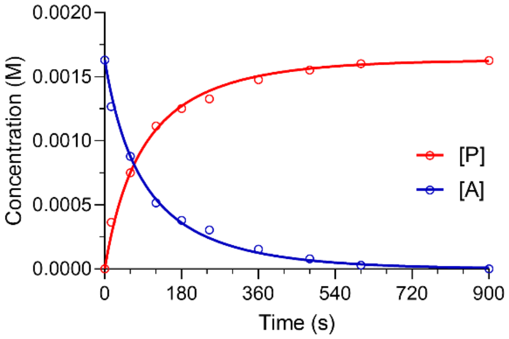

Supplementary figure 54. Experimental points and fitting curves for the ligation between **LYRAA-CO(SeESNa)** and **2**, corresponding to Exp 1. P = LYRAACTAFS, A = LYRAA-CO(SeESNa).

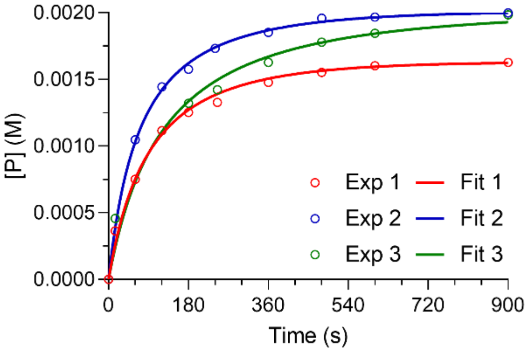

Supplementary figure 55. Experimental points and fitting curves for the replicate ligations between **LYRAA-CO(SeESNa)** and **2**. P = **LYRAACTAFS**.

Supplementary table 12. Calculated k_1 for the ligation between **LYRAA-CO(SeESNa)** and **2**.

Exp	k_1 (M^{-1} s^{-1})
**1**	4.57
**2**	5.53
**3**	3.30
**Mean**	**4.5 ± 0.9**

6.13. LYRAP-CO(SeESNa)

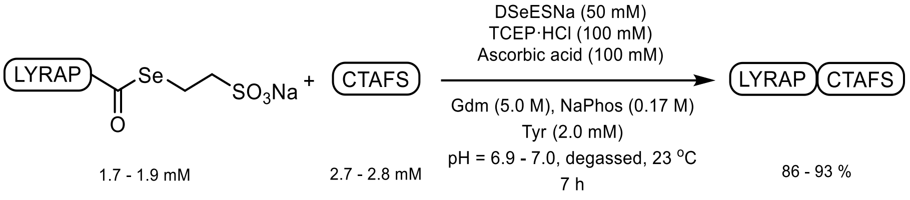

(Exp 3) **LYRAP-CO(SeESNa)** (1.202 mg, 1.1 x 10^{-3} mmol) and **2** (1.038 mg, 1.6 x 10^{-3} mmol) were weighed in separate polypropylene tubes. In a separate vial, DSeESNa (17.0 mg, 0.03 mmol), TCEP·HCl (17.2 mg, 0.06 mmol) and ascorbic acid (10.6 mg, 0.06 mmol) were dissolved in a mixture of Tyr (20.0 mM, 60 µL) and guanidine / phosphate buffer (500 µL). The pH was adjusted to 7.05. The ligation buffer was added to **2** and the pH checked (7.00, final volume = 600 µL). The resulting mixture was added to LYRAP-CO(SeESNa). The final pH, checked at the end of the reaction, was 7.0.

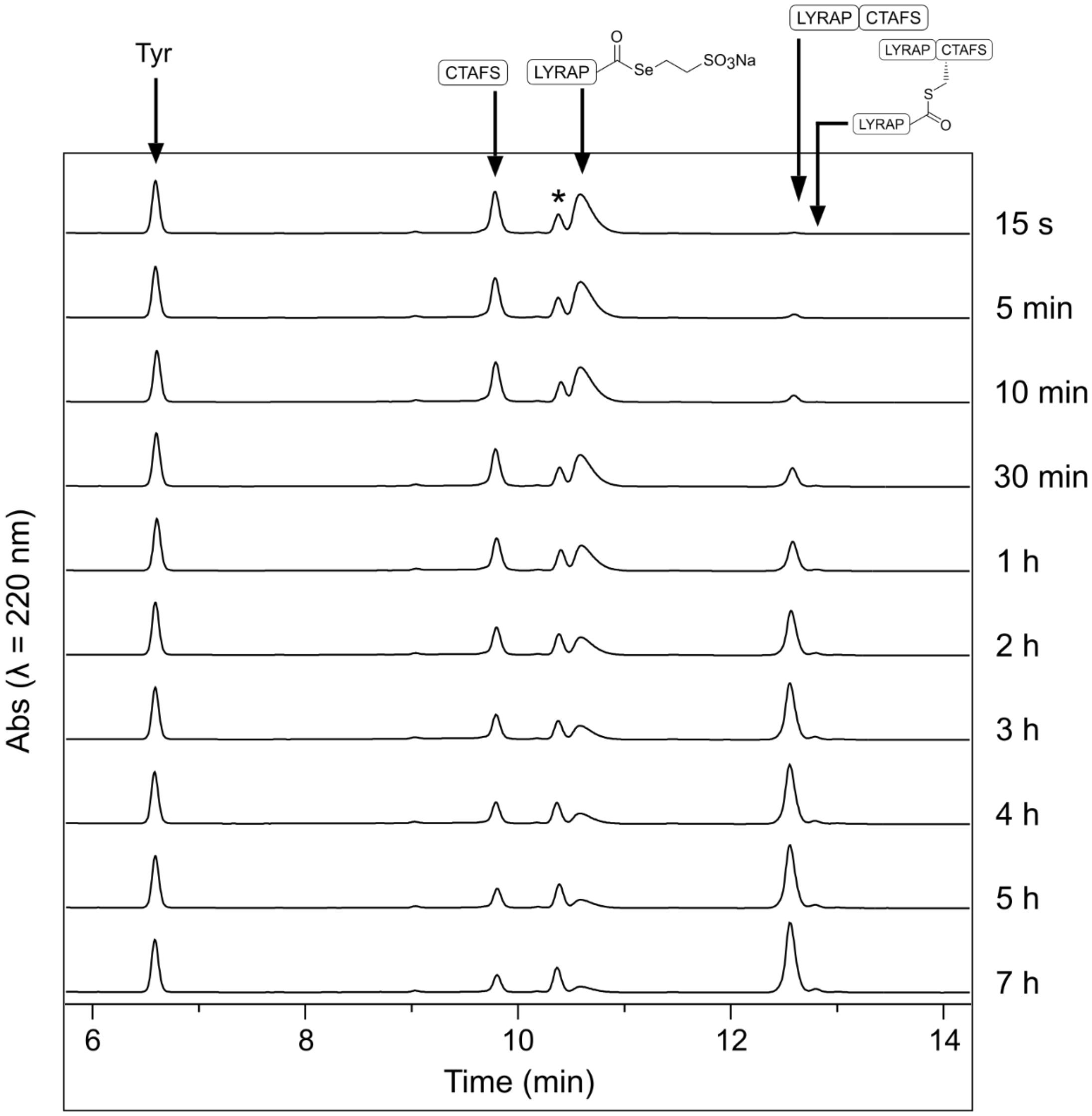

Supplementary figure 56. HPLC traces (220 nm) at given times of the ligation between **LYRAP-CO(SeESNa)** and **2**, corresponding to Exp 3. Column B, gradient 1. *: TCEP-SeESNa.

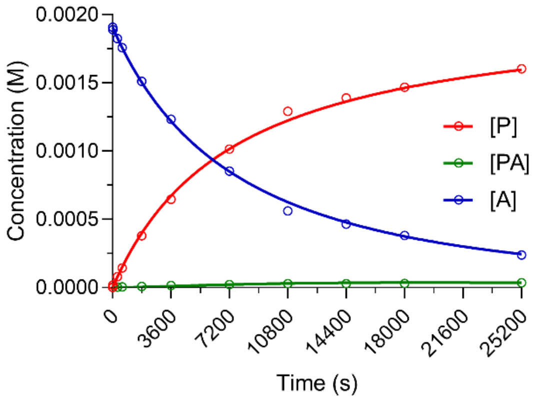

Supplementary figure 57. Experimental points and fitting curves for the ligation between **LYRAP-CO(SeESNa)** and **2**, corresponding to Exp 3. P = **LYRAPCTAFS**, PA = **LYRAPC(LYRAP-[COS])TAFS**, A = **LYRAP-CO(SeESNa)**.

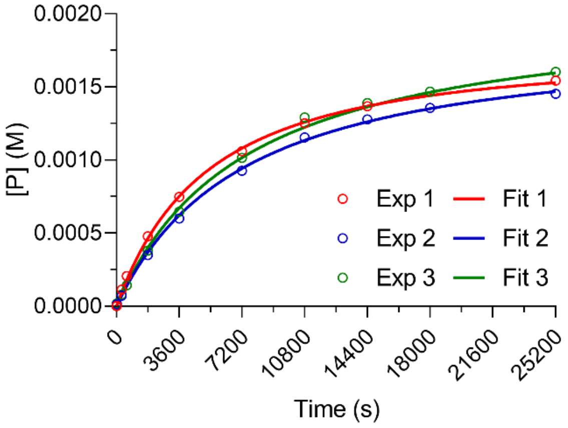

Supplementary figure 58. Experimental points and fitting curves for the replicate ligations between **LYRAP-CO(SeESNa)** and **2**. P = **LYRAPCTAFS**.

Supplementary table 13. Calculated k_1 for the ligation between **LYRAP-CO(SeESNa)** and **2**.

Exp	k_1 (M^{-1} s^{-1})
**1**	0.0720
**2**	0.0558
**3**	0.0522
**Mean**	**0.060 ± 0.009**

6.14. LYRAP-CO(SeESNa), catalyzed by PhSeH

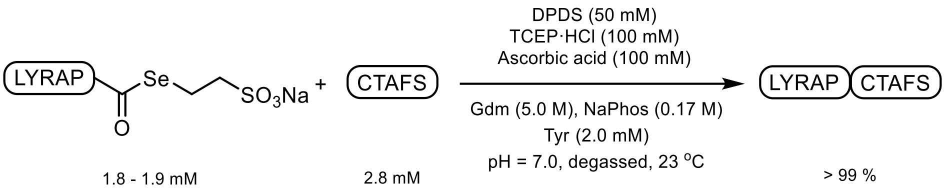

LYRAP-CO(SeESNa) (1.134 mg, 1.1 x 10^{-3} mmol) and **2** (1.067 mg, 1.7 x 10^{-3} mmol) were weighed in separate polypropylene tubes. In a separate vial, DPDS (9.36 mg, 0.03 mmol), TCEP·HCl (17.0 mg, 0.06 mmol) and ascorbic acid (10.6 mg, 0.06 mmol) were dissolved in a mixture of Tyr (20.0 mM, 60 μL) and guanidine / phosphate buffer (500 μL). The pH was adjusted to 7.1. The ligation buffer was added to **2** and the pH checked (7.0, final volume = 600 μL). The resulting mixture was added to LYRAP-CO(SeESNa). The final pH, checked at the end of the reaction, was 7.0.

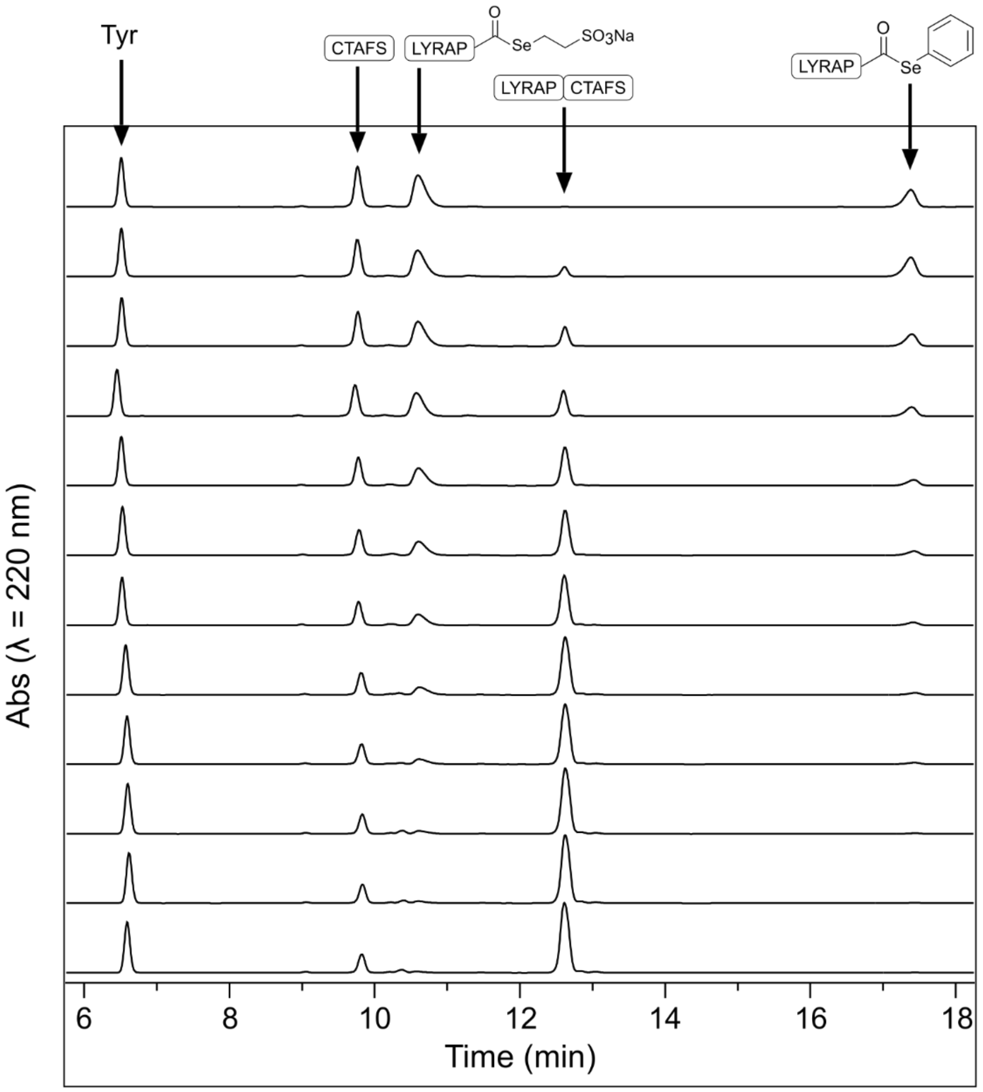

Supplementary figure 59. HPLC traces (220 nm) at given times of the ligation between **LYRAP-CO(SeESNa)** and **2**, catalyzed by DPDS. Column B, gradient 1.

The [**LYRAPCTAFS**]$_t$ data was analyzed and fitted according to the following kinetic model:

$$\textbf{LYRAP-CO(SeESNa)} + \textbf{2} \xrightarrow{k_{obs}} \textbf{LYRAPCTAFS}$$

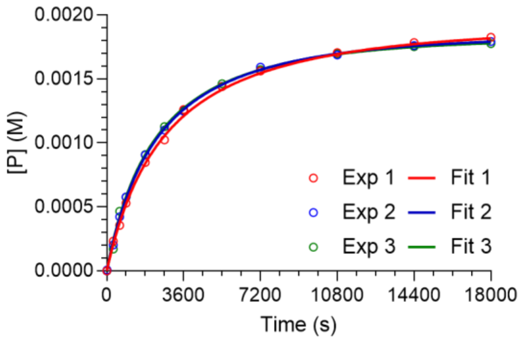

Supplementary figure 60. Experimental points and fitting curves for the replicate ligations between **LYRAP-CO(SeESNa)** and **2**. P = LYRAPCTAFS.

Supplementary table 14. Calculated rate constants for the ligation between **LYRAP-CO(SeESNa)** and **2**.

Exp	k_{obs} (M^{-1} s^{-1})
**1**	0.150
**2**	0.168
**3**	0.176
**Mean**	**0.16 ± 0.01**

6.15. LYRAS-CO(SeESNa)

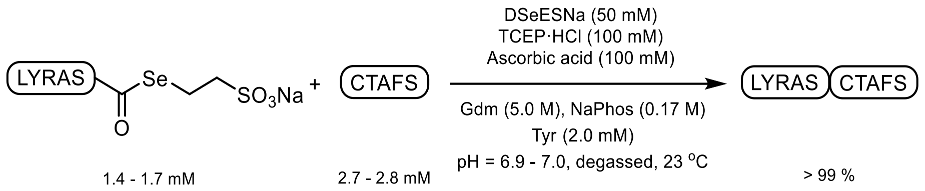

(Exp 2) **LYRAS-CO(SeESNa)** (1.008 mg, 9.7 x 10^{-4} mmol) and **2** (1.049 mg, 1.6 x 10^{-3} mmol) were weighed in separate polypropylene tubes. In a separate vial, DSeESNa (17.2 mg, 0.03 mmol), TCEP·HCl (17.0 mg, 0.06 mmol) and ascorbic acid (10.6 mg, 0.06 mmol) were dissolved in a mixture of Tyr (20.0 mM, 60 µL) and guanidine / phosphate buffer (500 µL). The pH was adjusted to 7.05. The ligation buffer was added to **2** and the pH checked (7.00, final volume = 600 µL). The resulting mixture was added to LYRAS-CO(SeESNa). The final pH, checked at the end of the reaction, was 7.0.

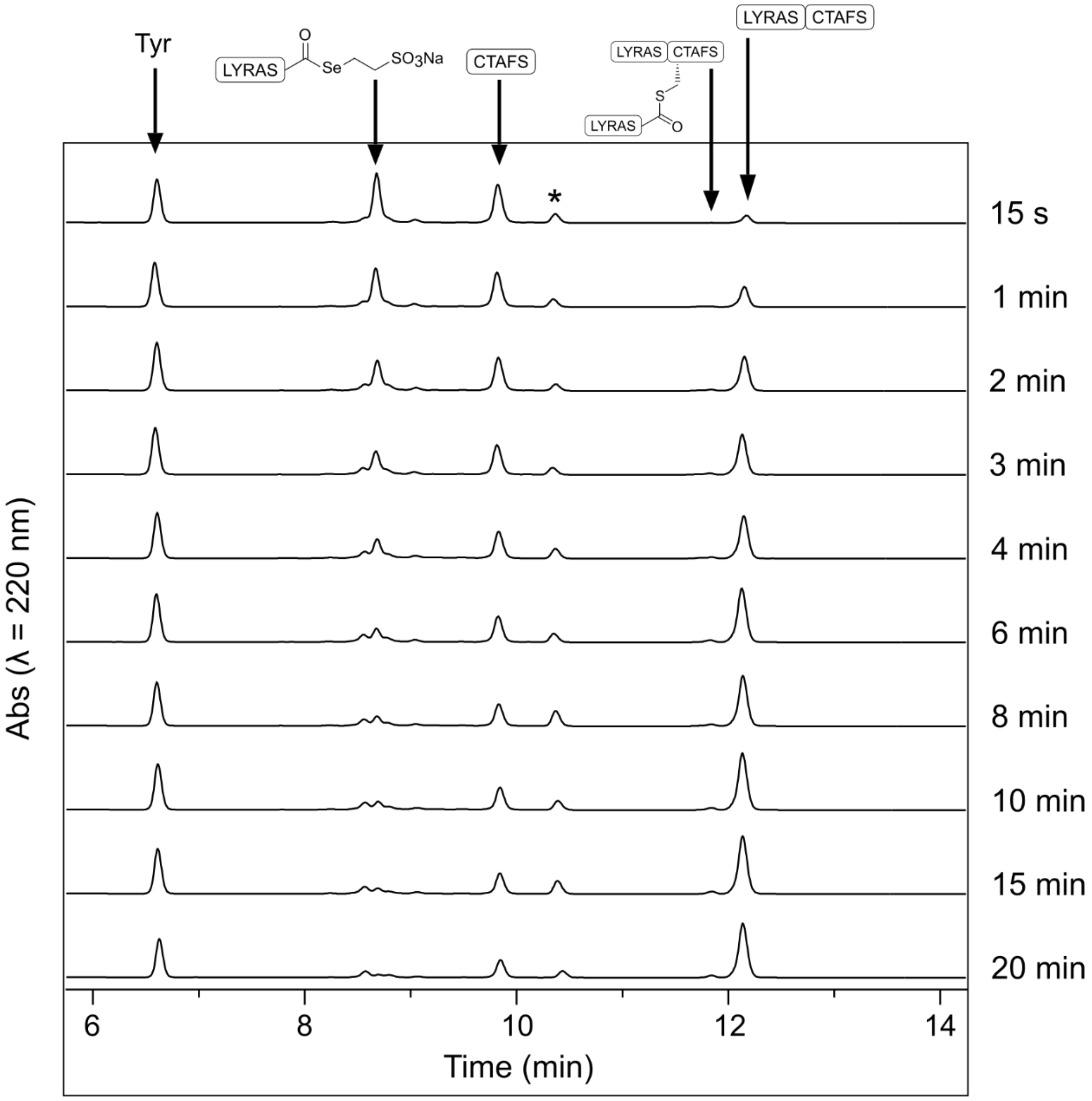

Supplementary figure 61. HPLC traces (220 nm) at given times of the ligation between **LYRAS-CO(SeESNa)** and **2**, corresponding to Exp 2. Column B, gradient 1. *: TCEP-SeESNa.

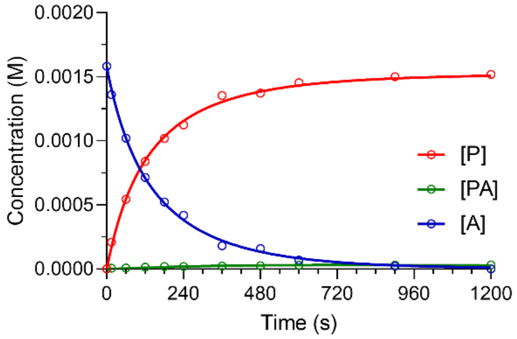

Supplementary figure 62. Experimental points and fitting curves for the ligation between **LYRAS-CO(SeESNa)** and **2**, corresponding to Exp 2. P = **LYRASCTAFS**, PA = **LYRASC(LYRAS-[COS])TAFS**, A = **LYRAS-CO(SeESNa)**.

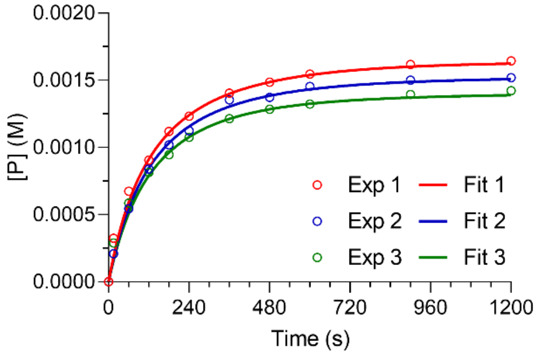

Supplementary figure 63. Experimental points and fitting curves for the replicate ligations between **LYRAS-CO(SeESNa**) and **2**. P = LYRASCTAFS.

Supplementary table 15. Calculated k_1 for the ligation between **LYRAS-CO(SeESNa)** and **2**.

Exp	k_1 (M^{-1} s^{-1})
**1**	3.36
**2**	2.95
**3**	3.15
**Mean**	**3.2 ± 0.2**

Epimerization experiment.

In an HPLC vial, 50 µL of the reaction crude were mixed with 50 µL of **LYRAsCTAFS** (2 mM in H_2O/ACN 1:1 with TFA 0.05%). The reaction crude, the epimer and the mixture were analyzed by RP-HPLC. The results show a >99% de.

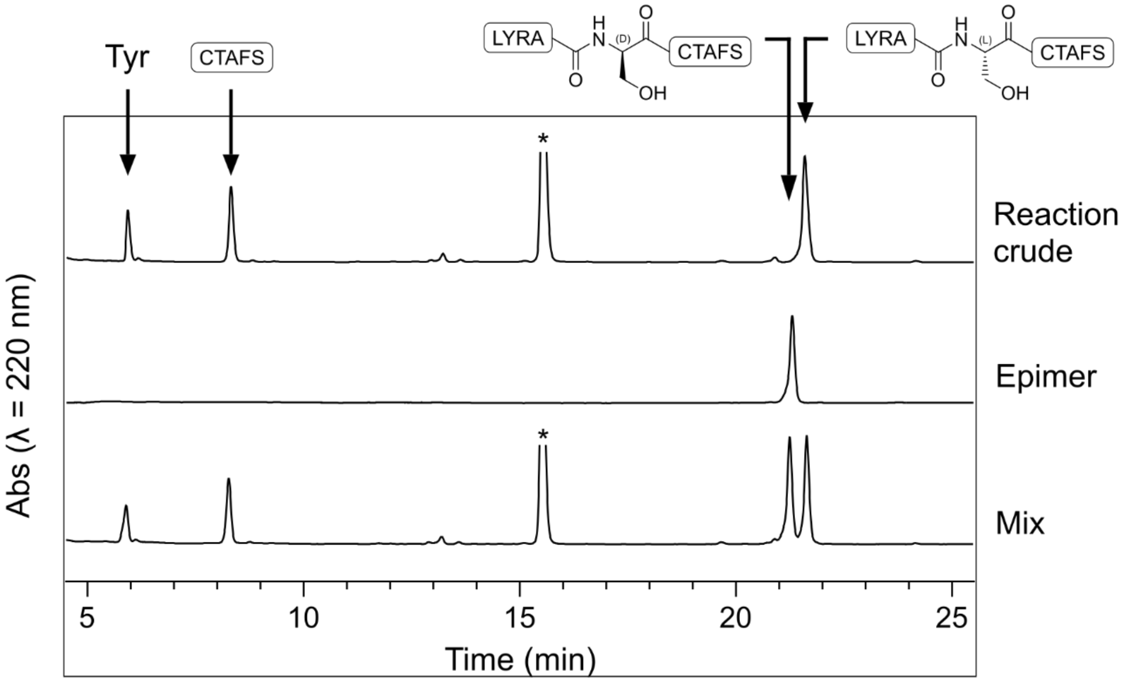

Supplementary figure 64. HPLC traces (220 nm) of the epimerization test in the ligation of **LYRAS-CO(SeESNa)** and **CTAFS**. Column B, gradient 2. *: TCEP-SeESNa.

7. Chemical synthesis of Cardiotoxin A5 (CTX5)

7.1. F1-CO(Nbz)-CONH$_2$

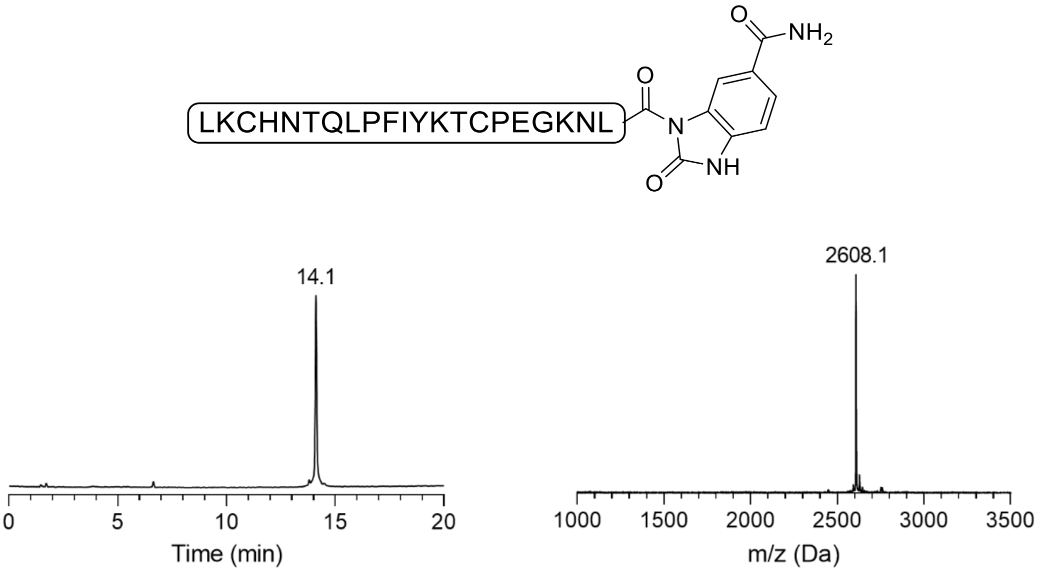

Supplementary figure 65. HPLC (220 nm) and MALDI-TOF MS of **F1-CO(Nbz)-CONH$_2$**. Column A, gradient 1. Rt = 15.7 min. Calculated $(M+H)^+$: 2608.1. Observed m/z: 2608.1.

7.2. F2-CO(Dbz)

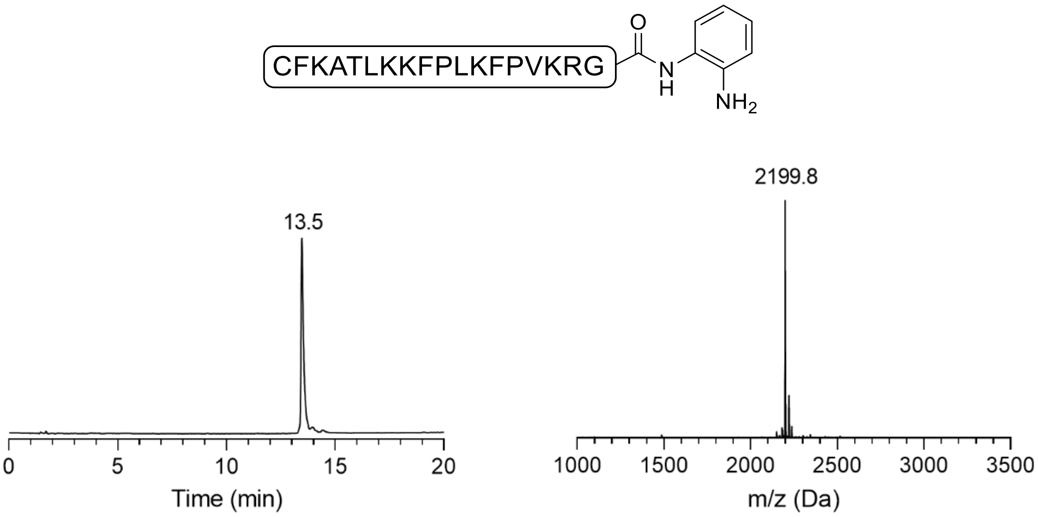

Supplementary figure 66. HPLC (220 nm) and MALDI-TOF MS of **F2-CO(Dbz)**. Column A, grandient 1. Rt = 13.4 min. Calculated $(M+H)^+$: 2199.8. Observed m/z: 2199.8.

7.3. F3

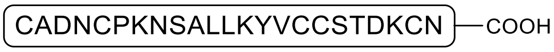

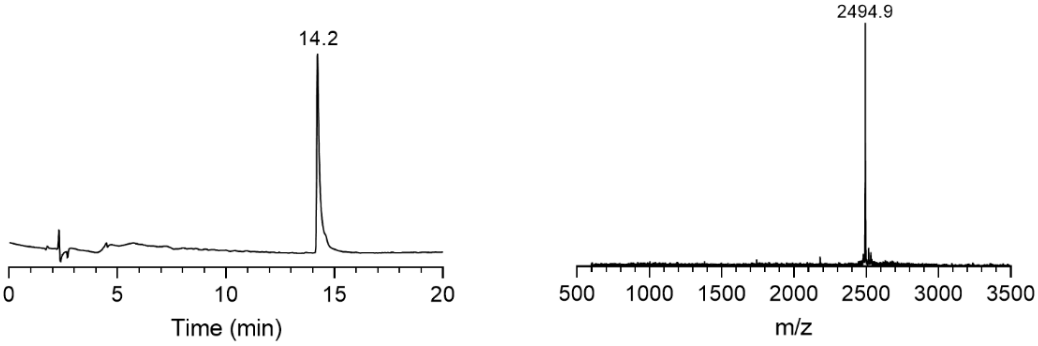

Supplementary figure 67. HPLC (220 nm) and MALDI-TOF MS of **F3**. Column A, gradient 1. Rt = 14.2 min. Calculated $(M+H)^+$: 2494.9. Observed m/z: 2494.9.

7.4. F1-F2-CO(Dbz)

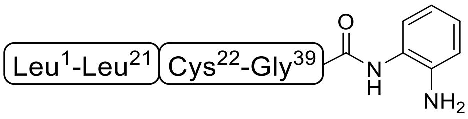

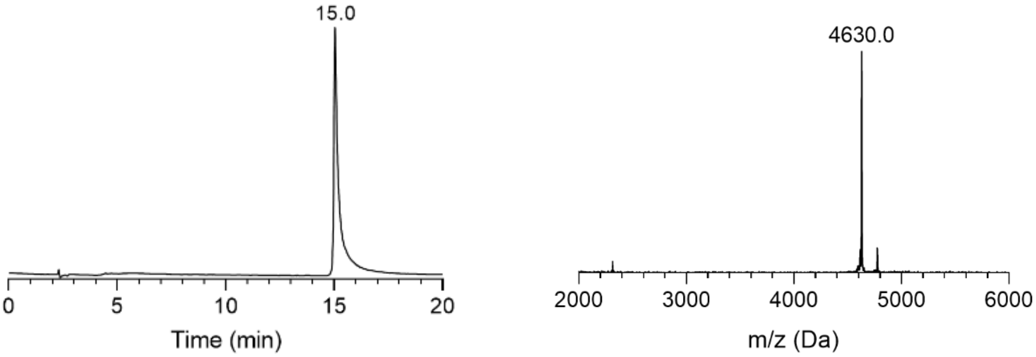

Supplementary figure 68. HPLC (220 nm) and MALDI-TOF MS of purified **F1-F2-CO(Dbz)**. Column A, gradient 1. t = 15.0 min. Calculated $(M+H)^+$: 4629.7. Observed m/z: 4630.0.

7.5. F1-F2-CO(MESNa)

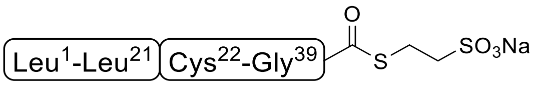

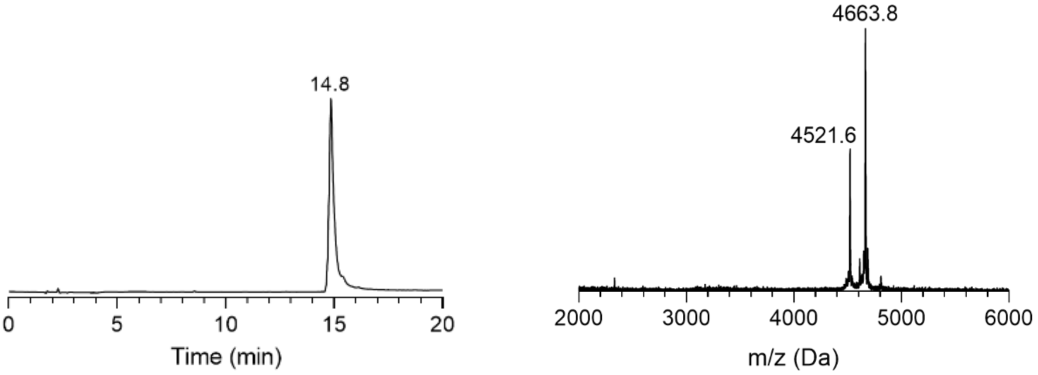

Supplementary figure 69. HPLC (220 nm) and MALDI-TOF MS of **F1-F2-CO(MESNa)**. Column A, gradient 1. t = 14.8 min. Calculated $(M+H)^+$ for **F1-F2-CO(MESH)**: 4663.7. Observed m/z: 4663.8. Calculated $(M+H)^+$ for **F1-F2-CO(thiolactone)**: 4521.5. Observed m/z: 4521.6.

7.6. F1-F2-CO(MESNa) + SeESNa, stability test

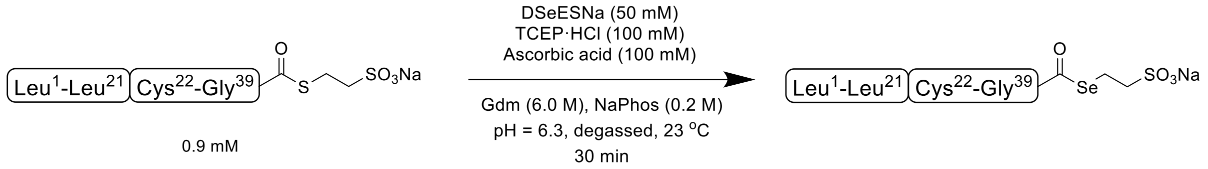

F1-F2-CO(MESNa+thiolactone) (0.820 mg, 1.8 x 10^{-4} mmol) was dissolved in a degassed guanidine / phosphate buffer (200 μL, pH = 6.3) containing DSeESNa (50 mM), TCEP·HCl (100 mM) and ascorbic acid (100 mM), and incubated at 23 °C.

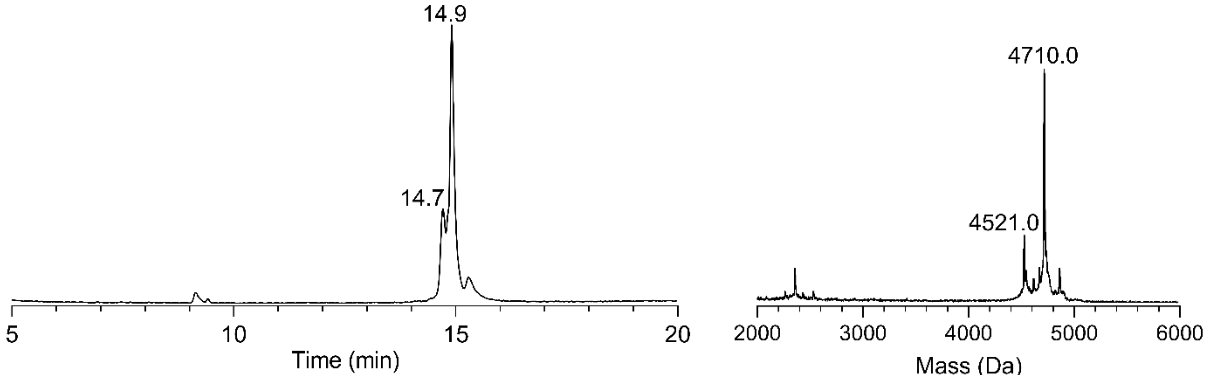

Supplementary figure 70. HPLC trace (220 nm) at 30 min of the incubation of F1-F2-CO(MESNa) and SeESNa. Column B, gradient 1. t = 14.7 min (F1-F2-CO(thiolactone)), t = 14.9 min (F1-F2-CO(SeESH)). Calculated $(M+H)^+$ for **F1-F2-CO(SeESH)**: 4710.7. Observed m/z: 4710.0.

7.7. F1-F2-F3

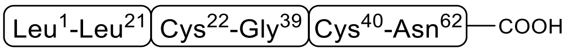

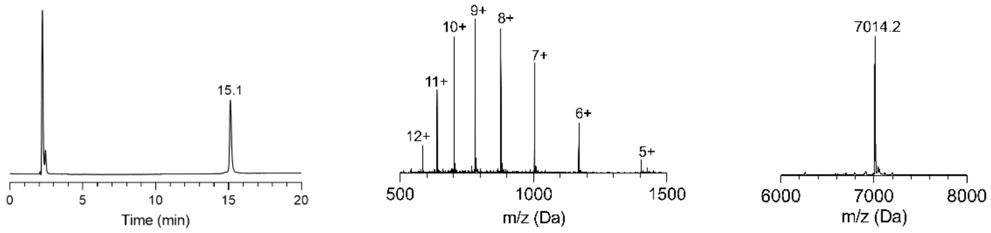

Supplementary figure 71. HPLC (220 nm) and ESIMS of **F1-F2-F3** (peptide purified and dissolved in folding conditions at time = 0 h). Column C, gradient 1. t = 15.1 min. Found m/z: 585.82 (M+12), 638.66 (M+11), 702.41 (M+10), 780.33 (M+9), 877.76 (8+), 1002.98 (7+), 1170.02 (6+), 1403.75 (5+). Calculated M^+ (average isotopes): 7014.4. Observed deconvoluted mass: 7014.2.

7.8. Oxidation and folding: CTX5

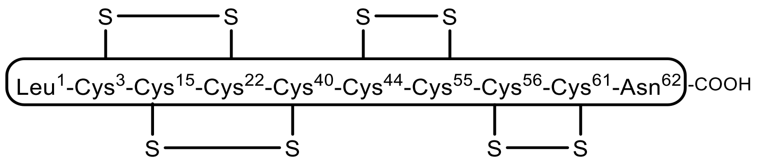

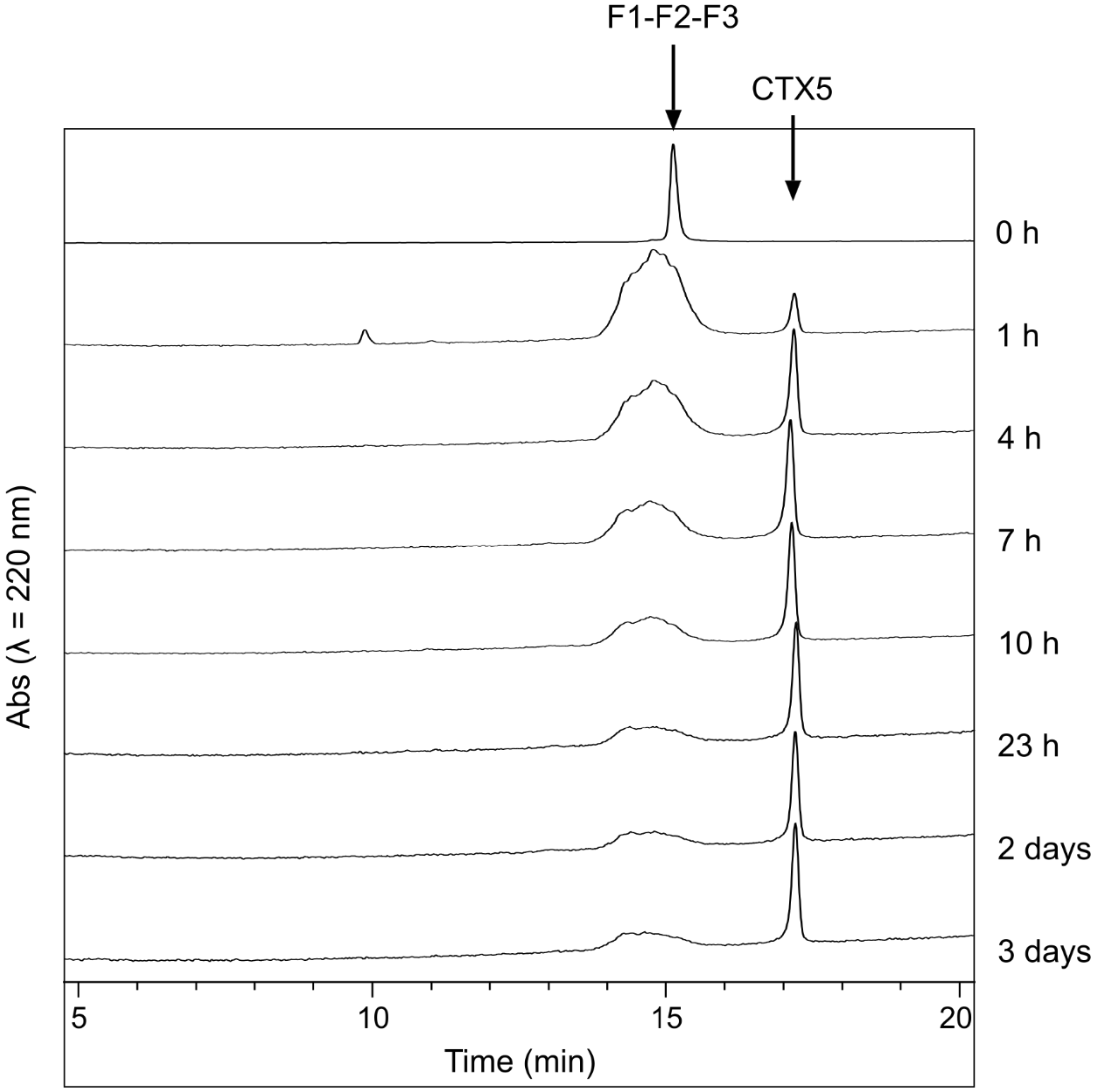

Supplementary figure 72. HPLC traces (220 nm) at given times of the **CTX5** folding. Column C, gradient 1.

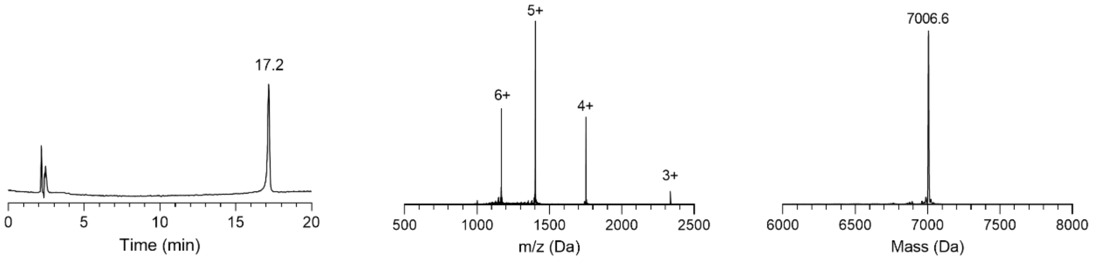

Supplementary figure 73. HPLC (220 nm) and ESIMS of pure folded **CTX5**. Column C, gradient 1. t = 17.2 min. Found m/z: 1168.81 (M+6), 1402.32 (M+5), 1752.54 (M+4), 2336.52 (M+3). Calculated M^+ (average isotopes): 7006.4. Observed deconvoluted mass: 7006.6.

7.9. NMR of CTX5

^{1}H NMR

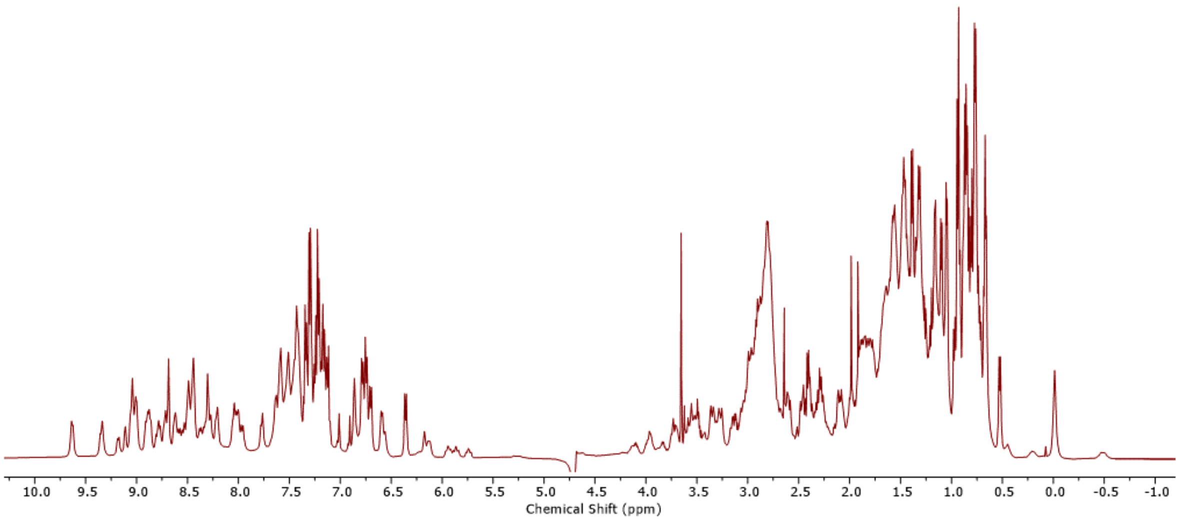

NOESY

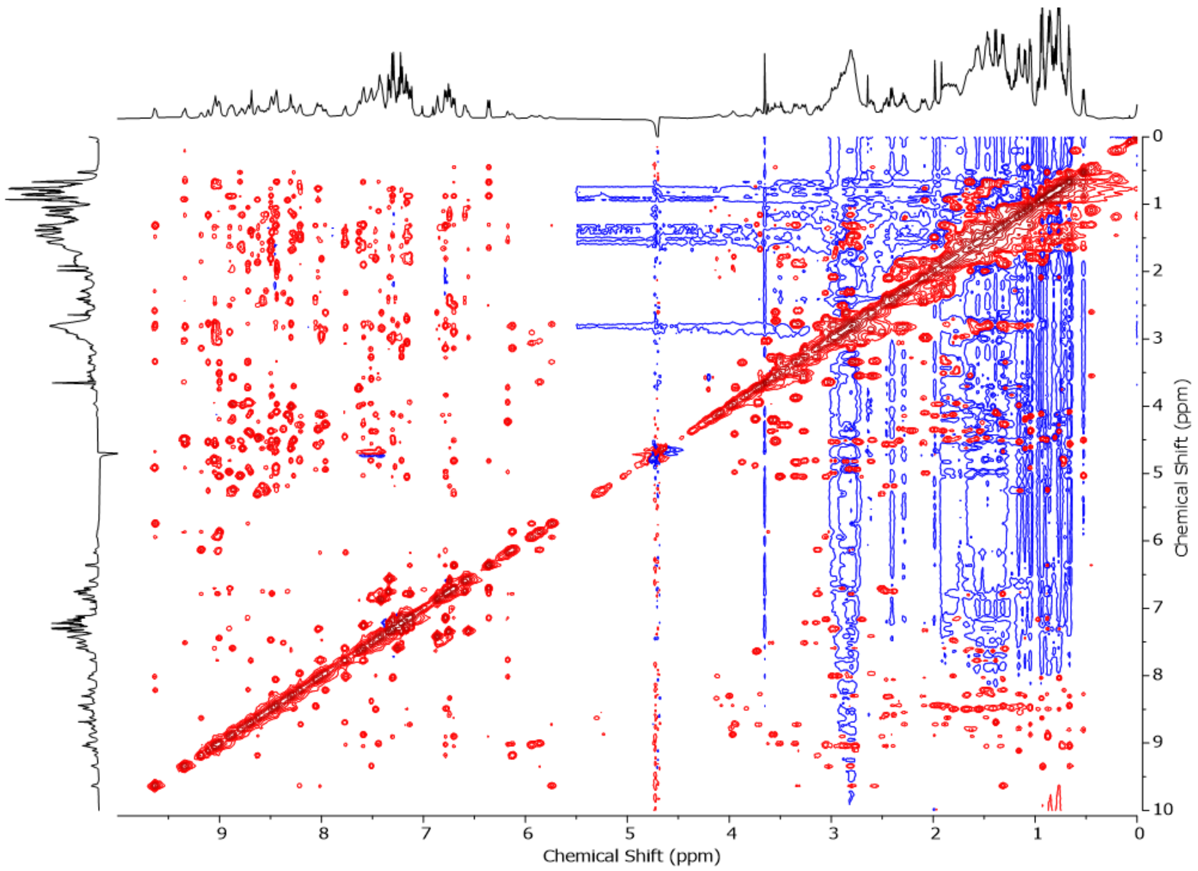

Supplementary figure 74. ^{1}H and NOESY NMR spectra of **CTX5**.

8. Protein expression and SeESNa-catalyzed Expressed Protein Ligation

8.1. Expression of His$_6$-SUMO2(ΔG)-GyrA-CBD

The plasmid pSS01, encoding for His$_6$-SUMO2(ΔG)-GyrA-CBD, was described in a previous report.[5] It was designed using the pGEX-4T-1 template, a commercial vector from Cytiva (stock code: 28-9545-49). This vector includes a thrombin cleavage site and resistance to ampicillin.

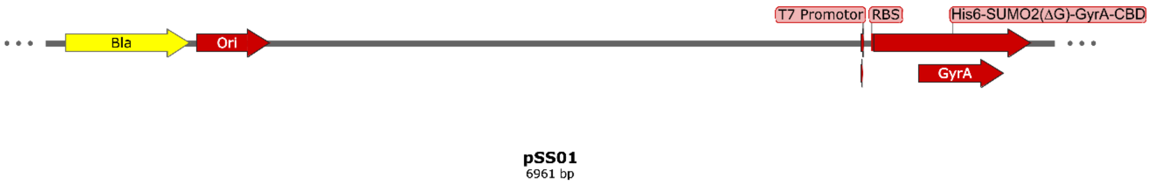

MGSSHHHHHH^{10} GADEKPKEGV^{20} KTENNDHINL^{30} KVAGQDGSVV^{40} QFKIKRHTPL^{50}
SKLMKAYCER^{60} QGLSMRQIRF^{70} RFDGQPINET^{80} DTPAQLEMED^{90} EDTIDVFQQQ^{100}
TGCITGDALV^{110} ALPEGESVRI^{120} ADIVPGARPN^{130} SDNAIDLKVL^{140} DRHGNPVLAD^{150}
RLFHSGEHPV^{160} YTVRTVEGLR^{170} VTGTANHPLL^{180} CLVDVAGVPT^{190} LLWKLIDEIK^{200}
PGDYAVIQRS^{210} AFSVDCAGFA^{220} RGKPEFAPTT^{230} YTVGVPGLVR^{240} FLEAHHRDPD^{250}
AQAIADELTD^{260} GRFYYAKVAS^{270} VTDAGVQPVY^{280} SLRVDTADHA^{290} FITNGFVSHA^{300}
TGLTGLNSGL^{310} TTNPGVSAWQ^{320} VNTAYTAGQL^{330} VTYNGKTYKC^{340} LQPHTSLAGW^{350}
EPSNVPALWQ^{360} LQ^{362}

His$_6$-SUMO2(ΔG)-GyrA-CBD

8.2. Synthesis of His$_6$-SUMO2(ΔG)-CTAFS by SeESNa-catalyzed EPL

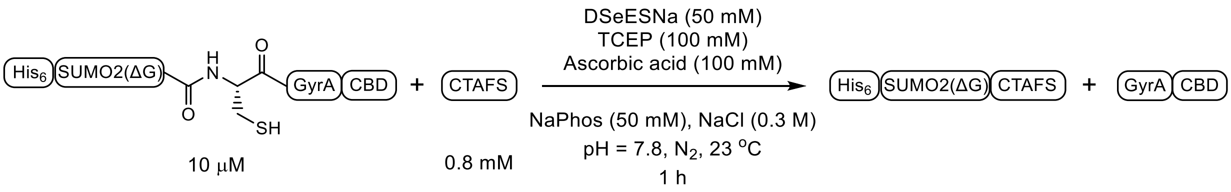

Product characterization by MS spectrometry.

To obtain a sample for MS spectrometry characterization, a reaction performed in the same conditions as shown in the scheme was purified after 1 h by IMAC (0.3 mL of column bed volume). A small amount of TCEP·HCl (~ 5 mg) was added to the eluate, and the sample was directly analyzed by UPLC-qToF MS.

Control experiment with MESNa.

A solution containing the ligation buffer (2x, 1 mL) and **2** was prepared in a polypropylene tube: MESNa (32.8 mg, 0.2 mmol), TCEP·HCl (28.7 mg, 0.1 mmol), and **2** (1.05 mg, 2.0 mmol) were weighed and dissolved in Ni-NTA buffer (0.9 mL). The pH was adjusted to 8.0 with $NaOH_{(aq)}$ (10 M) and Ni-NTA buffer (total final volume = 1 mL). To this ligation buffer (0.300 mL) was added a solution of Ni-NTA buffer (0.254 mL, pH = 8.0), and the resulting mixture incubated with His$_6$-SUMO2(ΔG)-GyrA-CBD (0.046 mL, 0.006 µmol, 130 µM stock). The reaction was flushed with N_2 and run overnight. Aliquots of the ligation were withdrawn at indicated times and stored at -20 °C for SDS-PAGE analysis. The ligation pH, checked at the beginning and the end of the reaction, was 7.8.

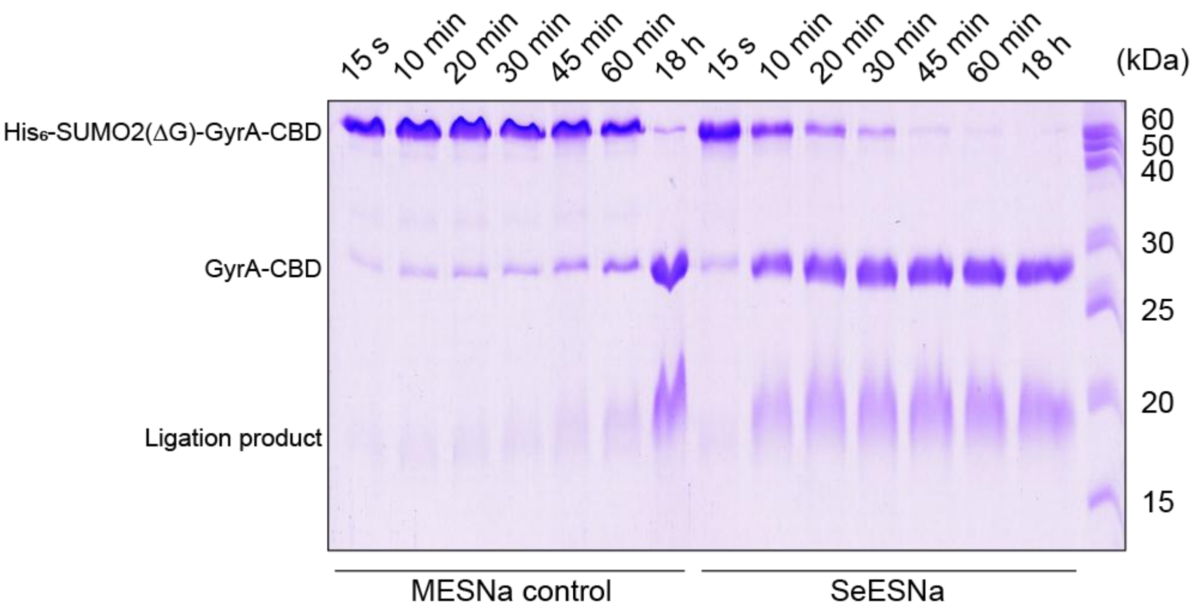

Supplementary figure 75. SDS-PAGE monitoring of the one-pot SeESNa-catalyzed EPL between His$_6$-SUMO2(ΔG)-GyrA-CBD and **2**.

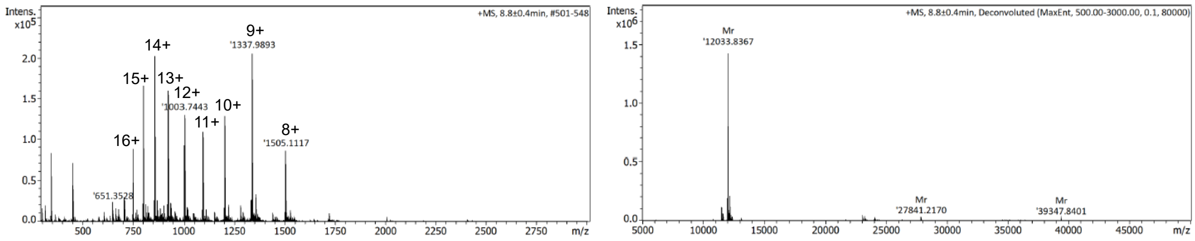

Supplementary figure 76. Raw HRMS of the one-pot SeESNa-catalysed EPL between His$_6$-SUMO2(ΔG)-GyrA-CBD and **2** after 1 h of reaction. 708.8179 (M+17), 753.0565 (M+16), 803.1936 (M+15), 860.4931 (M+14), 926.6080 (M+13), 1003.7443 (M+12), 1094.9018 (M+11), 1204.3912 (M+10), 1337.9893 (M+9), 1505.1117 (M+8). Calculated $(M + H)^+$ (main isotopes) for $C_{516}H_{810}N_{159}O_{165}S_5$ (His$_6$-SUMO2(ΔG)-CTAFS): 12032.8405, observed deconvoluted mass: 12033.8367.

8.3. Cloning and expression of His_6-SUMO-Shh^{1-159}-GyrA

The plasmid PECF795, used for Shh gene cloning, was generated by previously described protocols.[6] Briefly, PECF795 is based on a pET SUMO vector including the Smt3 gene (the yeast analog of mammalian SUMO1, found in *Saccharomyces cerevisiae*), an N-terminal His_6 tag, a SUMO protease cleavage site and resistance to kanamycin. The ORF of Sino Biological cDNA HG10372-M was cloned (sequence identical to GenBank reference NM_000193.2, full-length human Shh clone) into this vector. It is 6096 bp long and digestion with HindIII and EcoRV results in two fragments of 3971 and 2125 bp.

The plasmid pTXB1-CfaC-Gyra, used for cloning the GyrA gene, was obtained from the Addgene repository (stock number: 122950), encoding for GyrA-His_6.[7]

PECF795 and pTXB1-CfaC-Gyra were used as templates to generate the plasmid encoding His_6-SUMO-Shh^{1-159}-GyrA: first, the GyrA sequence was PCR-amplified using pTXB1-CfaC-Gyra (0.4 ng) as template DNA, and the GyrA-F and GyrA-R (0.5 µM each) primers. PCR parameters were: 50 µL of reaction volume, 98 °C for 30 seconds, 30 cycles of 98 °C for 10 seconds, 60 °C for 30 seconds and 72 °C for 4 min, with a final extension at 72 °C for 7 min. The amplified sequence encodes for $Shh^{153-159}$-GyrA-$Shh^{160-166}$. For the insertion of the GyrA gene into PECF795, PCR was performed using PECF795 (10 ng) as template DNA and the previously amplified GyrA sequence (215 ng) as primer. PCR was performed with the following parameters: 20 µL of total volume, 98 °C for 30 seconds, 30 cycles of 98 °C for 15 seconds and 72 °C for 9 min, with a final extension at 72 °C for 10 min. 2 µL of the PCR product were incubated with KLD reaction mixture for 10 min at 25 °C. Bacterial plasmid stocks were prepared by transforming *E. coli* DH5alpha cells with 5 µL of the KLD processed PCR product which is the plasmid encoding His_6-SUMO-Shh^{1-159}-GyrA. The resulting plasmid is 6690 bp long and digestion with HindIII and HpaI results in two fragments of 3915 and 2775 bp.

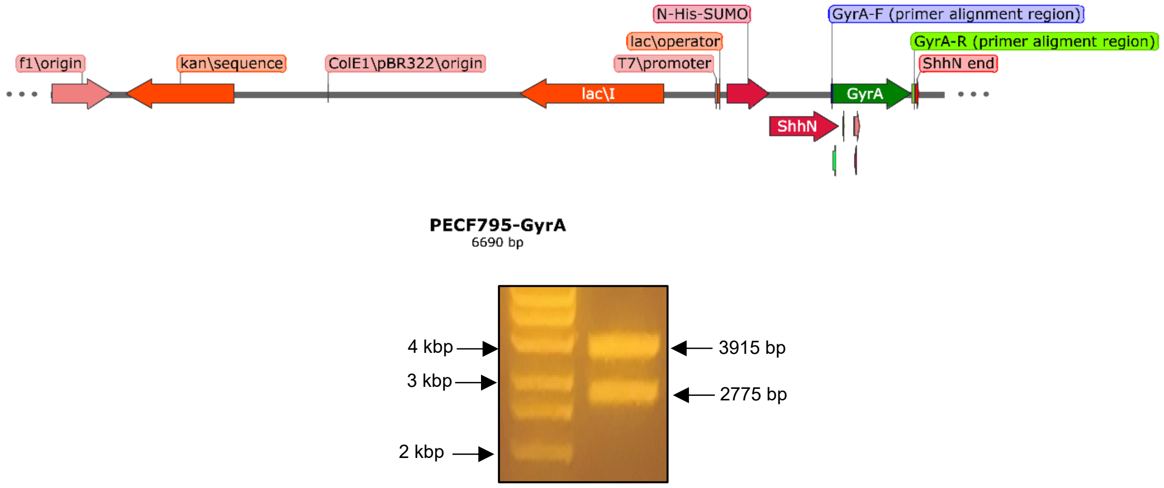

Supplementary figure 77. Sequence map and restriction enzyme profile of PECF795-GyrA, the plasmid encoding His_6-SUMO-Shh^{1-159}-GyrA.

Supplementary table 16. Oligonucleotides used in PCR

Oligonucletide	Sequence (5’ → 3’)
GyrA-F	GAG TCC AAG GCA CAT ATC CAC TGC ATC ACG GGA
GyrA-R	GTT CTC TGC TTT CAC CGA CGA AGC GTG GCT GAC

Supplementary table 17. Encoded amino acid sequences used in the cloning of His$_6$-SUMO-Shh$^{(1-159)}$-GyrA.

Protein	Sequence
Mxe GyrA N198A (pTXB1-CfaC-GyrA)	CITGDALVAL^{10} PEGESVRIAD^{20} IVPGARPNSD^{30} NAIDLKVLDR^{40}<br>HGNPVLADRL^{50} FHSGEHPVYT^{60} VRTVEGLRVT^{70} GTANHPLLCL^{80}<br>VDVAGVPTLL^{90} WKLIDEIKPG^{100} DYAVIQRSAF^{110} SVDCAGFARG^{120}<br>KPEFAPTTYT^{130} VGVPGLVRFL^{140} EAHHRDPDAQ^{150} AIADELTDGR^{160}<br>FYYAKVASVT^{170} DAGVQPVYSL^{180} RVDTADHAFI^{190} TNGFVSHA^{198}
Shh wt	CGPGRGFGKR^{10} RHPKKLTPLA^{20} YKQFIPNVAE^{30} KTLGASGRYE^{40}<br>GKISRNSERF^{50} KELTPNYNPD^{60} IIFKDEENTG^{70} ADRLMTQRCK^{80}<br>DKLNALAISV^{90} MNQWPGVKLR^{100} VTEGWDEDGH^{110} HSEESLHYEG^{120}<br>RAVDITTSDR^{130} DRSKYGMLAR^{140} LAVEAGFDWV^{150} YYESKAHIHC^{160}<br>SVKAENSVAA^{170} KSGG^{174}
His$_6$-SUMO-Shh (PECF795)	MGSSHHHHHH^{10} GSDSEVNQEA^{20} KPEVKPEVKP^{30} ETHINLKVSD^{40}<br>GSSEIFFKIK^{50} KTTPLRRLME^{60} AFAKRQGKEM^{70} DSLRFLYDGI^{80}<br>RIQADQTPED^{90} LDMEDNDIIE^{100} AHREQIGGCG^{110} PGRGFGKRRH^{120}<br>PKKLTPLAYK^{130} QFIPNVAEKT^{140} LGASGRYEGK^{150} ISRNSERFKE^{160}<br>LTPNYNPDII^{170} FKDEENTGAD^{180} RLMTQRCKDK^{190} LNALAISVMN^{200}<br>QWPGVKLRVT^{210} EGWDEDGHHS^{220} EESLHYEGRA^{230} VDITTSDRDR^{240}<br>SKYGMLARLA^{250} VEAGFDWVYY^{260} ESKAHIHCSV^{270} KAENSVAAKS^{280}<br>GG^{282}
Shh$^{153-159}$-GyrA-Shh$^{160-166}$	ESKAHIHCIT^{10} GDALVALPEG^{20} ESVRIADIVP^{30} GARPNSDNAI^{40}<br>DLKVLDRHGN^{50} PVLADRLFHS^{60} GEHPVYTVRT^{70} VEGLRVTGTA^{80}<br>NHPLLCLVDV^{90} AGVPTLLWKL^{100} IDEIKPGDYA^{110} VIQRSAFSVD^{120}<br>CAGFARGKPE^{130} FAPTTYTVGV^{140} PGLVRFLEAH^{150} HRDPDAQAIA^{160}<br>DELTDGRFYY^{170} AKVASVTDAG^{180} VQPVYSLRVD^{190} TADHAFITNG^{200}<br>FVSHACSVKA^{210} EN^{212}
His$_6$-SUMO-Shh$^{1-159}$-GyrA	MGSSHHHHHH^{10} GSDSEVNQEA^{20} KPEVKPEVKP^{30} ETHINLKVSD^{40}<br>GSSEIFFKIK^{50} KTTPLRRLME^{60} AFAKRQGKEM^{70} DSLRFLYDGI^{80}<br>RIQADQTPED^{90} LDMEDNDIIE^{100} AHREQIGGCG^{110} PGRGFGKRRH^{120}<br>PKKLTPLAYK^{130} QFIPNVAEKT^{140} LGASGRYEGK^{150} ISRNSERFKE^{160}<br>LTPNYNPDII^{170} FKDEENTGAD^{180} RLMTQRCKDK^{190} LNALAISVMN^{200}<br>QWPGVKLRVT^{210} EGWDEDGHHS^{220} EESLHYEGRA^{230} VDITTSDRDR^{240}<br>SKYGMLARLA^{250} VEAGFDWVYY^{260} ESKAHIHCIT^{270} GDALVALPEG^{280}<br>ESVRIADIVP^{290} GARPNSDNAI^{300} DLKVLDRHGN^{310} PVLADRLFHS^{320}<br>GEHPVYTVRT^{330} VEGLRVTGTA^{340} NHPLLCLVDV^{350} AGVPTLLWKL^{360}<br>IDEIKPGDYA^{370} VIQRSAFSVD^{380} CAGFARGKPE^{390} FAPTTYTVGV^{400}<br>PGLVRFLEAH^{410} HRDPDAQAIA^{420} DELTDGRFYY^{430} AKVASVTDAG^{440}<br>VQPVYSLRVD^{450} TADHAFITNG^{460} FVSHACSVKA^{470} ENSVAAKSGG^{480}

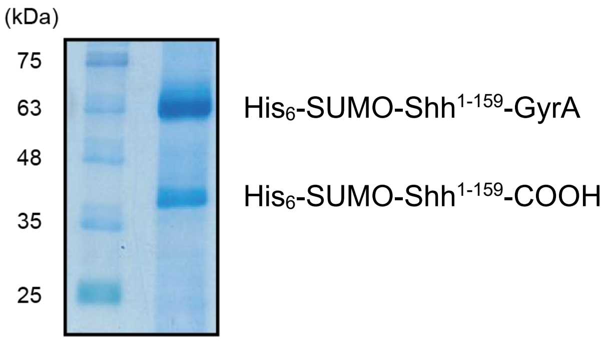

Supplementary figure 78. SDS-PAGE analysis of the His$_6$-SUMO-Shh$^{1-159}$-GyrA stock.

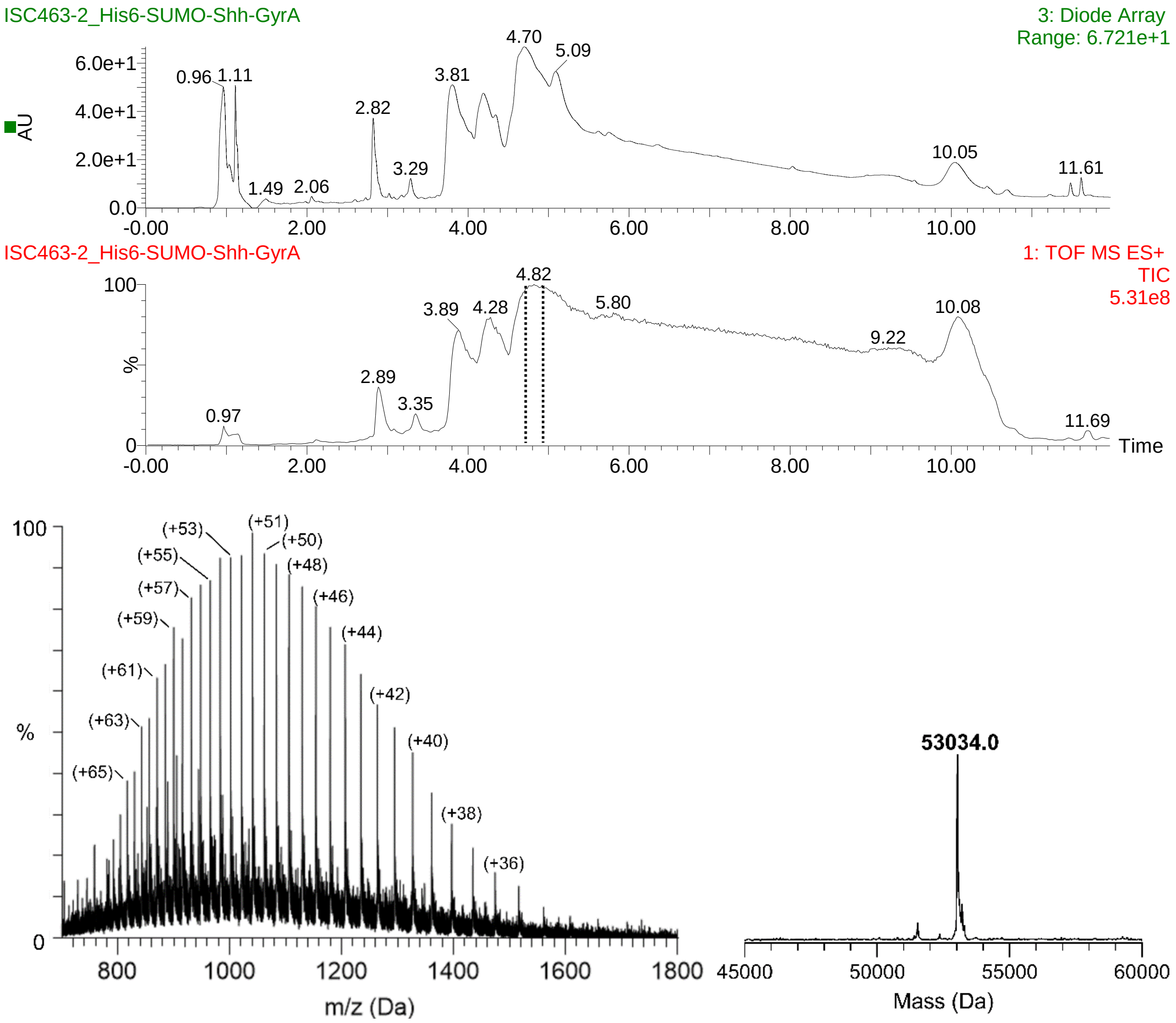

Supplementary figure 79. Raw UPLC-MS and deconvoluted MS of His$_6$-SUMO-Shh$^{1-159}$-GyrA stock (column: ACQUITY Premier Protein BEH C4 300 Å 1.7 µm 2.1 x 100 mm, 5 –> 60% of CH_3CN 0.1% formic acid over H_2O 0.1% formic acid). t = 4.70 min m/z: 842.8187 (M+63), 856.3741 (M+62), 870.4269 (M+61), 884.9038 (M+60), 899.9003 (M+59), 915.3509 (M+58), 931.4610 (M+57), 948.0592 (M+56), 965.2595 (M+55), 983.0786 (M+54), 1001.6082 (M+53), 1020.8915 (M+52), 1040.8695 (M+51), 1061.6907 (M+50), 1083.3309 (M+49), 1105.8572 (M+48), 1129.4086 (M+47), 1153.9200 (M+46), 1179.5557 (M+45), 1206.2568 (M+44), 1234.3591 (M+43), 1263.7437 (M+42), 1294.4918 (M+41), 1326.8323 (M+40), 1360.7776 (M+39), 1396.6141 (M+38), 1434.4043 (M+37), 1474.1115 (M+36), 1516.2898 (+35). Calculated M^+ (average isotopes) for $C_{2337}H_{3660}N_{676}O_{714}S_{12}$: 53035.7250, observed deconvoluted mass: 53034.0.

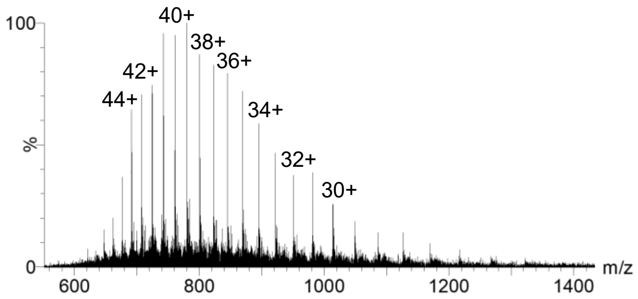

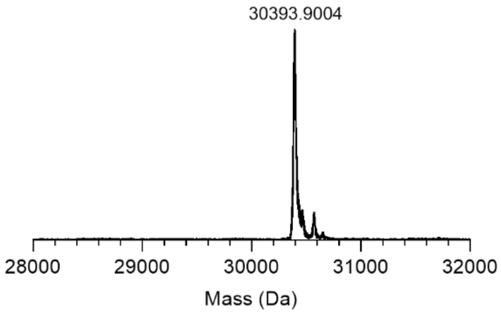

Supplementary figure 80. Raw ESI-MS and deconvoluted MS of His_6-SUMO-Shh^{1-159}-COOH. t = 4.19 min. m/z: 676.5154 (M+45), 691.7546 (M+44), 707.8148 (M+43), 724.6236 (M+42), 742.2965 (M+41), 760.050 (M+40), 780.2866 (M+39), 800.8482 (M+38), 822.4380 (M+37), 845.2824 (M+36), 869.4597 (M+35), 894.0039 (M+34), 922.0930 (M+33), 950.8151 (M+32), 981.44194 (M+31), 1014.2533 (M+30), 1049.1053 (M+29). Calculated M^+ (average isotopes) for $C_{1329}H_{2080}N_{394}O_{410}S_8$: 30394.0870, observed deconvoluted mass: 30393.9004.

8.4. Synthesis of His$_6$-SUMO-Shh$^{1-174}$-PEG-His$_6$ and His$_6$-SUMO-Shh$^{1-174}$-K(PEG)-Biotin by SeESNa-catalyzed EPL

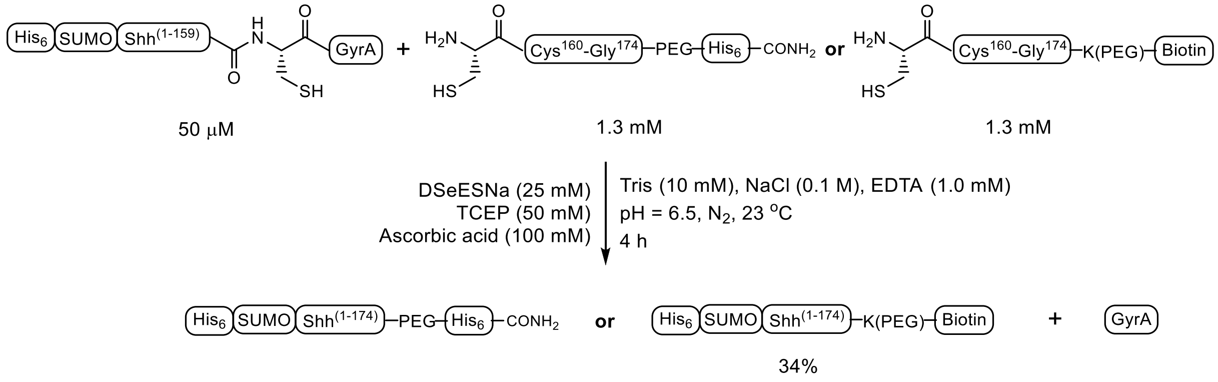

Ligation buffer (2x).

DSeESNa (28.0 mg, 0.05 mmol), TCEP.HCl (28.7 mg, 0.1 mmol) and ascorbic acid (35.2 mg, 0.2 mmol) were weighed and dissolved in STE buffer (0.900 mL). The pH was adjusted to 6.6 with $NaOH_{(aq)}$ (10 M) and STE (final total volume = 1 mL).

His$_6$-SUMO-Shh$^{1-174}$-PEG-His$_6$.

Cys^{160}-Gly^{174}-PEG-His$_6$ (2.52 mg, 1.0 x 10^{-3} mmol) was weighed in a polypropylene tube and dissolved in STE (0.266 mL) and ligation buffer 2x (0.350 mL). Then, 133 µL of His$_6$-SUMO-Shh$^{1-159}$-GyrA (0.133 mL, 0.035 µmol, 262 µM stock) were added to the mixture. The reaction was flushed with N_2 and left at 23 °C with occasional stirring. The pH, checked at the beginning and at the end of the reaction, was 6.5. Aliquots of the ligation were withdrawn at indicated times and stored at -20 °C for SDS-analysis. After 4 h of reaction, TCEP.HCl (~ 5 mg) was added to the mixture, and the buffer was exchanged to STE (PD-10). The sample was concentrated to 0.5 mL (Amicon Ultra® - Centrifugal Filters -3K, 6500 rcf, 4 °C), and analysed by UPLC-qToF MS without further purification.

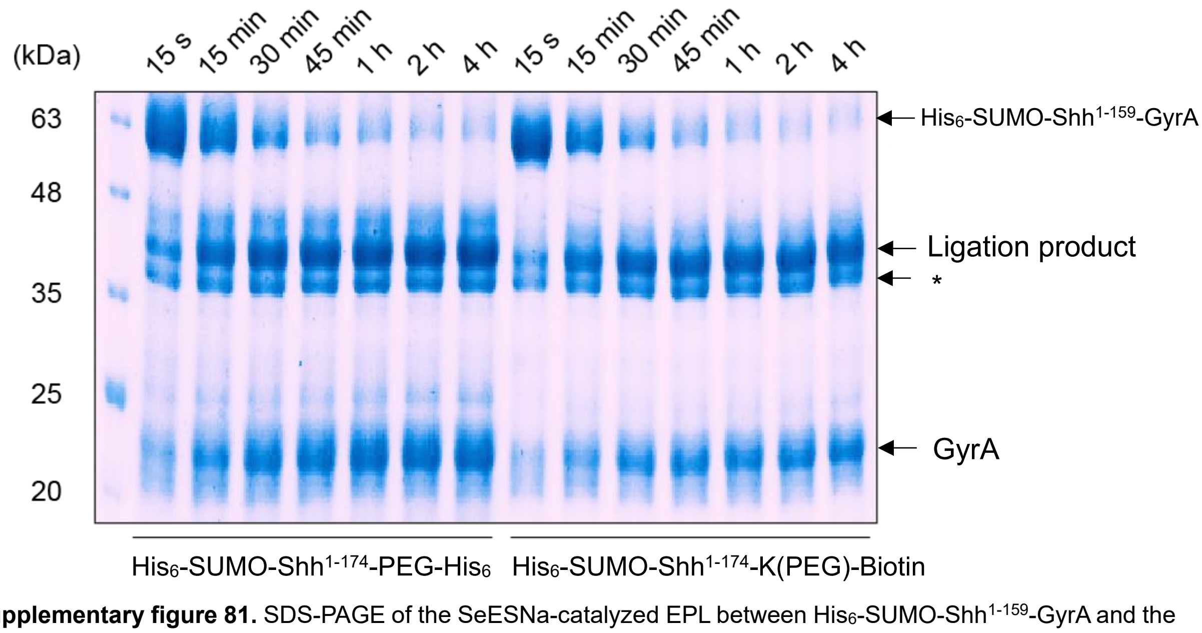

Supplementary figure 81. SDS-PAGE of the SeESNa-catalyzed EPL between His$_6$-SUMO-Shh$^{1-159}$-GyrA and the Shh Cys^{160}-Gly^{174}-tagged peptides. * = His$_6$-SUMO-Shh$^{1-159}$-COOH

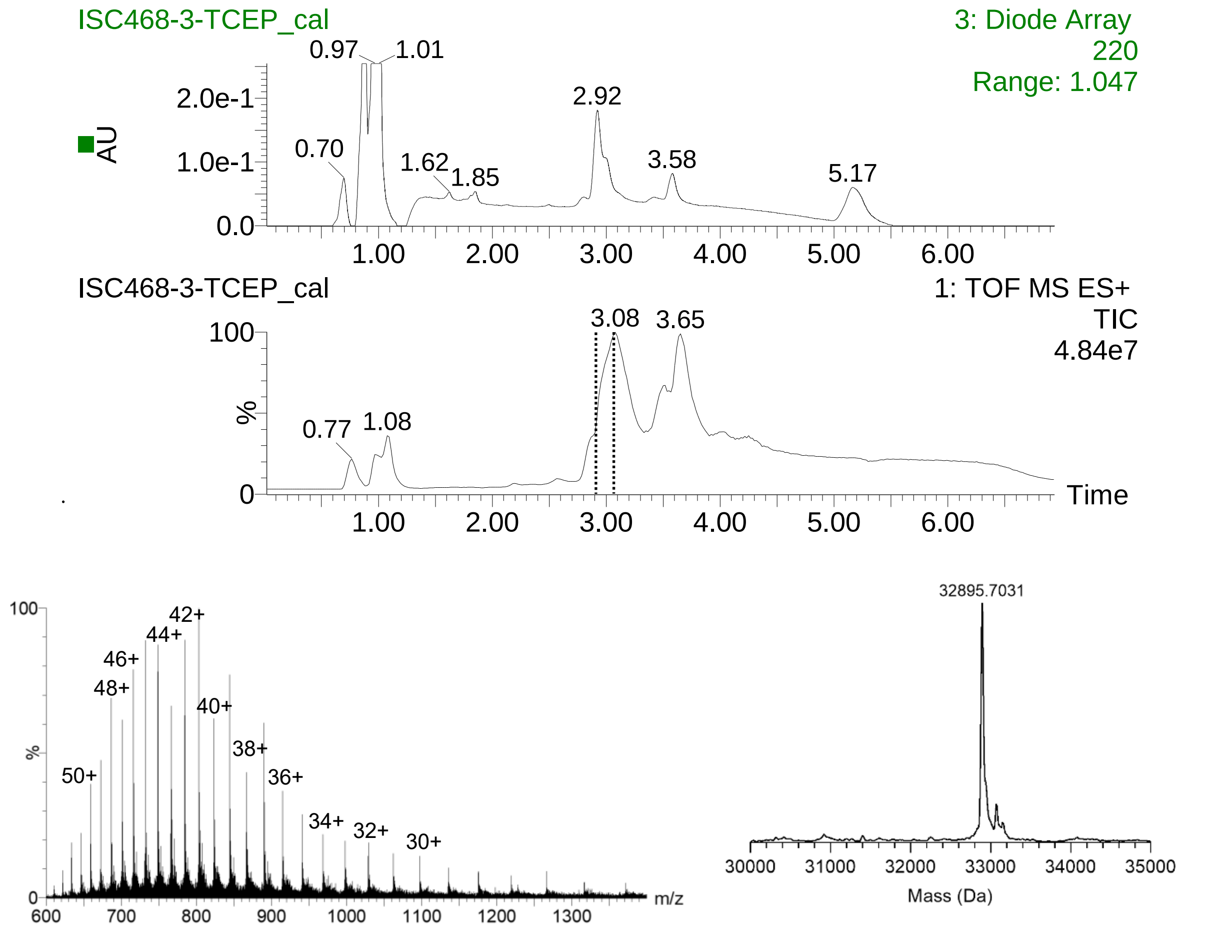

Supplementary figure 82. Raw UPLC-MS and deconvoluted MS of His$_6$-SUMO-Shh$^{1\text{-}174}$-PEG-His$_6$ ligation crude at 4 h (column: ACQUITY UPLC BEH300 C18 1.7 µm 2.1 x 100 mm, 10 –> 100% of CH_3CN 0.1% formic acid over H_2O 0.1% formic acid). t = 2.92 min. m/z: 621.7402 (M+53), 633.6812 (M+52), 645.9651 (M+51), 658.8492 (M+50), 672.2924 (M+49), 686.3640 (M+48), 700.8583 (M+47), 716.0950 (M+46), 732.0594 (M+45), 748.6668 (M+44), 766.0474 (M+43), 784.3237 (M+42), 803.3569 (M+41), 823.3209 (M+40), 844.5021 (M+39), 866.6000 (M+38), 890.0732 (M+37), 914.7126 (M+36), 940.9100 (M+35), 968.4686 (M+34), 997.8061 (M+33), 1029.0149 (M+32), 1062.2850 (M+31), 1097.5596 (M+30), 1135.3436 (M+29), 1175.8789 (M+28), 1219.3650 (M+27), 1266.2435 (M+26), 1317.0269 (M+25). Calculated M^+ (average isotopes) for $C_{1433}H_{2241}N_{433}O_{442}S_9$: 32895.8200, observed deconvoluted mass: 32895.7031. Peak at t = 0.70 min corresponds to Cys^{160}–Gly^{174}–PEG–His$_6$.

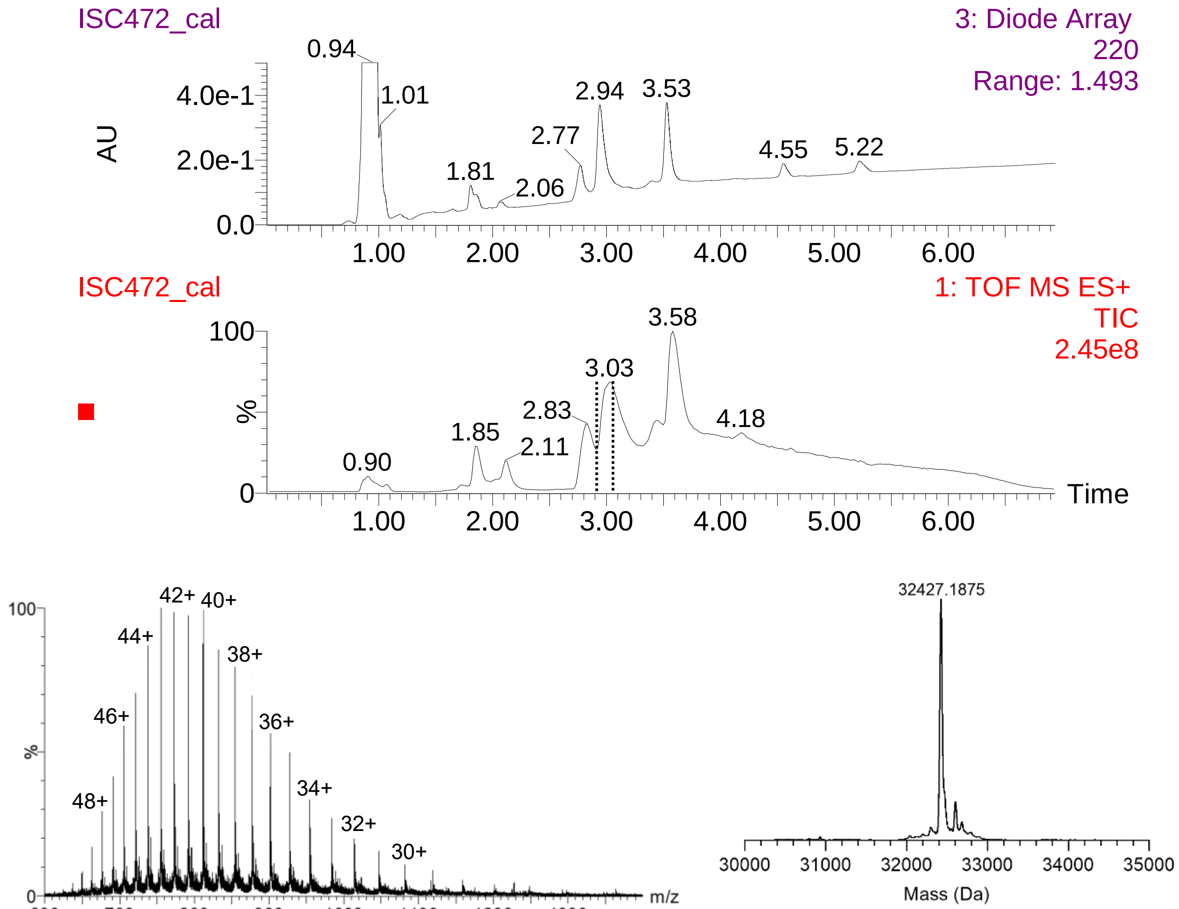

Supplementary figure 83. Raw UPLC-MS and deconvoluted MS of His6-SUMO-Shh[1-174]-K(PEG)-Biotin ligation crude at 4 h (column: ACQUITY UPLC BEH300 C18 1.7 µm 2.1 x 100 mm, 10 –> 100% of CH_3CN 0.1% formic acid over H_2O 0.1% formic acid). t = 2.94. m/z: 662.8191 (M+49), 676.5649 (M+48), 690.9373 (M+47), 705.9359 (M+46), 721.6036 (M+45), 737.9808 (M+44), 755.1418 (M+43), 773.0994 (M+42), 791.9297 (M+41), 811.6804 (M+40), 832.4373 (M+39), 854.3732 (M+38), 877.3802 (M+37), 901.7801 (M+36), 927.4877 (M+35), 954.8247 (M+34), 983.6667 (M+33), 1014.3754 (M+32), 1046.9694 (M+31), 1081.9298 (M+30), 1119.2786 (M+29), 1159.0322 (M+28), 1201.9996 (M+27). Calculated M^+ (average isotopes) for $C_{1413}H_{2225}N_{419}O_{439}S_{10}$: 32427.4370, observed deconvoluted mass: 32427.1875. Peak at t = 1.81 min corresponds to Cys[160]−Gly[174]−K(PEG)−Biotin. Peak at t = 2.77 min corresponds to His6-SUMO-Shh[1-159]-COOH.

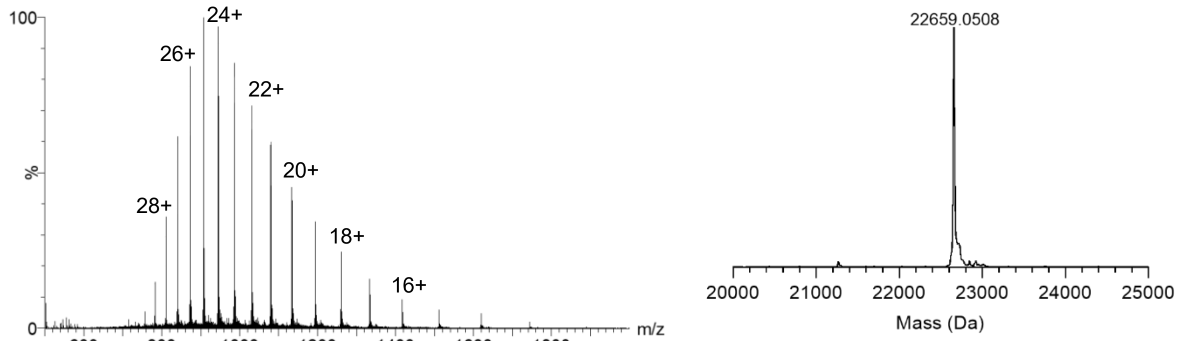

Supplementary figure 84. Raw and deconvoluted MS of GyrA. t = 3.53 min. m/z: 756.2950 (M+30), 782.3400 (M+29), 810.2444 (M+28), 840.2163 (M+27), 872.4547 (M+26), 907.3527 (M+25), 945.0751 (M+24), 986.1212 (M+23), 1030.9904 (M+22), 1079.9900 (M+21), 1133.9384 (M+20), 1193.5654 (M+19), 1259.8759 (M+18),

1333.8678 (M+17), 1417.1711 (M+16), 1511.6486 (M+15), 1619.5529 (M+14). Calculated M^+ for $C_{1008}H_{1582}N_{282}O_{305}S_4$ (average isotopes): 22658.6530, observed deconvoluted mass: 22659.0508.

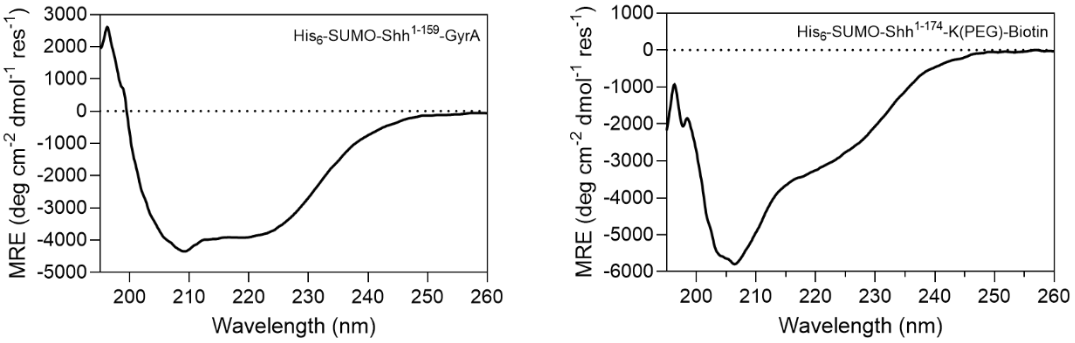

Supplementary figure 85. Circular dichroism of His$_6$-SUMO-Shh$^{1-159}$-GyrA and His$_6$-SUMO-Shh$^{1-174}$-K(PEG)-Biotin.

ELISA.

ELISA assays were carried out in Nunc Maxisorp F96 plates. Analysis was performed in a BioTek Synergy H1 microplate reader:

1- His$_6$-SUMO-Shh$^{1-174}$-K(PEG)-Biotin: plate wells were coated with streptavidin (50 µL, 5 µg/mL in PBS) at 4 °C overnight. After washing away the streptavidin, blocking buffer (120 µL) was added at room temperature and left for 1 h. Next, the solution was discarded, and the wells were washed with PT buffer (x4). His$_6$-SUMO-Shh$^{1-174}$-K(PEG)-Biotin (50 µL, 0.1 µM in PBS) was added and left for 1 h. After washing with PT buffer (4x), primary antibody 5E1 (50 µL, 50 nM in PBT buffer) was added and incubated for 1.5 h. Following washing with PT buffer, secondary antibody antimouse-HRP (50 µL, 1:6000 dilution in PBT buffer) was added and incubated for 1 h. Then, wells were washed with PT buffer (x4) and PBS (x4). TMB reagent (50 µL) was added, and the reactions were quenched with $HCl_{(aq)}$ (0.1 M) after 10 minutes.

2- Streptavidin control: plate wells were coated with streptavidin (50 µL, 5 µg/mL in PBS) at 4 °C overnight. Blocking buffer (120 µL) was added at room temperature and left for 1 h. The mixture was discarded and the wells were washed with PT buffer (x4). PBS (50 µL) was added and left for 1 h. After washing with PT buffer (4x), primary antibody 5E1 (50 µL, 50 nM in PBT buffer) was added and left for 1.5 h. Following washing with PT buffer (4x), secondary antibody antimouse-HRP (50 µL, 1:6000 dilution in PBT buffer) was added and left for 1 h. The wells were washed with PT buffer (x4) and PBS (x4). TMB reagent (50 µL) was added, and the reactions were quenched with $HCl_{(aq)}$ (0.1 M) after 10 minutes.

3- BSA control: plate wells were coated with BSA (50 µL, 5 µg/mL in PBS) at 4 °C overnight. The mixture was discarded and the wells were washed with PT buffer (x4). After washing with PT buffer (4x), primary antibody 5E1 (50 µL, 50 nM in PBT buffer) was added to the wells, and left for 1.5 h. After washing with PT buffer (4x), secondary antibody antimouse-HRP (50 µL, 1:6000 dilution in PBT) was added, and incubated for 1 h. Then, the wells were washed with PT (x4) times and PBS (x4). TMB reagent (50 µL) was added and the reactions were quenched with $HCl_{(aq)}$ (0.1 M) after 10 minutes.

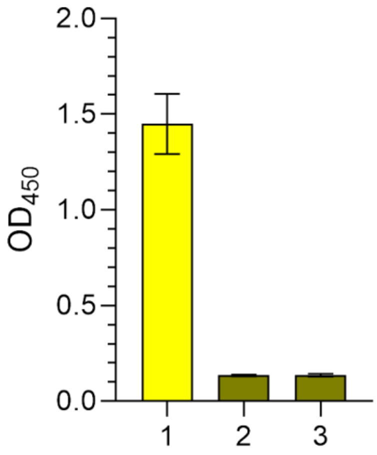

Supplementary figure 86. ELISA assay of His$_6$-SUMO-Shh$^{1-174}$-K(PEG)-Biotin with 5E1. 1: of His$_6$-SUMO-Shh$^{1-174}$-K(PEG)-Biotin. 2: Streptavidin control. 3: BSA control.

8.5. Analysis of the EPL between His$_6$-SUMO-Shh$^{1-159}$-GyrA and Cys^{160}-Gly^{174}-K(PEG)-Biotin catalyzed by MESNa, 4-MPAA, and SeESNa.

Three 2x ligation buffers were prepared using the STE buffer (total volume = 0.5 mL) and their pH was adjusted to 6.7:

A: MESNa 100 mM (8.21 mg, 0.05 mmol) and TCEP.HCl 50 mM (7.16 mg, 0.025 mmol)

B: 4-MPAA 100 mM (8.41 mg, 0.05 mmol) and TCEP.HCl 50 mM (7.16 mg, 0.025 mmol)

C: DSeESNa 50 mM (14.0 mg, 0.025 mmol), TCEP.HCl 100 mM (14.35 mg, 0.05 mmol) and ascorbic acid 200 mM (17.6 mg, 0.1 mmol)

In three separate polypropylene tubes, each ligation buffer (0.1 mL) and STE buffer (0.023 mL) were added over Cys^{160}-Gly^{174}-K(PEG)-Biotin (0.41 mg, 2.0 x 10^{-4} mmol). Then, 77 µL of His$_6$-SUMO-Shh$^{1-159}$-GyrA (0.010 µmol, 130 µM stock) were added to every mixture. The reactions were flushed with N_2 and left at 23 °C with occasional stirring. The pH, checked at the beginning and at the end of the reactions, was 6.6. Aliquots of the ligation were withdrawn at the indicated times. After 4 h of reaction, TCEP.HCl (~ 1 mg) was added to the mixture, and the buffer was exchanged to STE with 10% glycerol (Amicon Ultra® - Centrifugal Filters -3K, 6500 rcf, 4 °C). Samples were analyzed by UPLC-qToF MS to confirm the products.

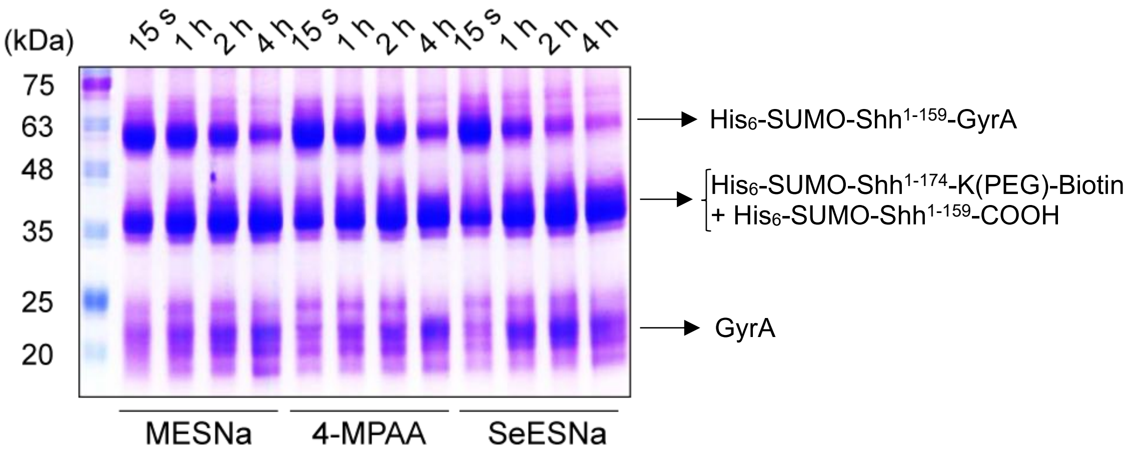

Supplementary figure 87. SDS-PAGE of the control experiment comparing MESNa, 4-MPAA, and SeESNa catalyzing the EPL between His$_6$-SUMO-Shh$^{1-159}$-GyrA and Shh Cys^{160}-Gly^{174}-K(PEG)-Biotin.

9. References

[1] Albericio, F.; Barany, G. An acid-labile anchoring linkage for solid-phase synthesis of C-terminal peptide amides under mild conditions. *Int. J. Peptide Protein Res.* **1987**, *30*, 206-216.

[2] Sánchez-Campillo, I.; Miguel-Gracia, J.; Karamanis, P.; Blanco-Canosa, J. B. A versatile *o*-aminoanilide linker for native chemical ligation. *Chem. Sci.* **2022**, *13*, 10904-101913.

[3] Yan, Y.; Remhof, A.; Rentsch, D.; Lee, Y.-S.; Cho, Y. W.; Züttel, A. Is $Y_2(B_{12}H_{12})_3$ the main intermediate in the decomposition process of $Y(BH_4)_3$? *Chem. Commun.* **2013**, *49*, 5234-5236.

[4] Stephens, S. J.; Jonich, M. J.; Determination of pKa using the half-volume method: A laboratory experiment. *J. Chem. Educ.* **1977**, *54*, 711

[5] Sommer, S.; Weikart, N. D.; Brockmeyer, A.; Janning, P.; Mootz, H. D. Expanded Click Conjugation of Recombinant Proteins with Ubiquitin-Like Modifiers Reveals Altered Substrate Preference of SUMO2-Modified Ubc9. *Angew. Chem. Int. Ed.* **2011**, *50*, 9888-9892.

[6] Palà-Pujadas, J.; Albericio, F.; Blanco-Canosa, J. B. Peptide Ligations by Using Aryloxy-carbonyl-*o*-methylaminoanilides: Chemical Synthesis of Palmitoylated Sonic Hedgehog. *Angew. Chem. Int. Ed.* **2018**, *57*, 16120-16125.

[7] Stevens, A. J.; Brown, Z. Z.; Shah, N. H.; Sekar, G.; Cowburn, D.; Muir, T. W.; Design of a Split Intein with Exceptional Protein Splicing Activity. *J. Am Chem Soc.* **2016**, *138*, 2162-2165.
